# Structural properties, polymorphism, and multiscale disorder unravel energy transport limitations in perylene diimide semiconductors

**DOI:** 10.1126/sciadv.aed0037

**Published:** 2026-05-27

**Authors:** Christopher J. H. Smalley, Colan E. Hughes, Tom Willhammar, Raj Pandya, Semion K. Saikin, Duncan N. Johnstone, Jeffrey Gorman, Jooyoung Sung, Gianni Jacucci, Paul A. Midgley, Demie M. Kepaptsoglou, Quentin M. Ramasse, Akshay Rao, Kenneth D. M. Harris, Sean M. Collins

**Affiliations:** ^1^ School of Chemistry, Cardiff University, Park Place, Cardiff CF10 3AT, UK.; ^2^ Department of Chemistry, Stockholm University, Svante Arrhenius väg 16C, 106 91 Stockholm, Sweden.; ^3^ School of Chemistry, Department of Chemistry, University of Warwick, Coventry, CV4 7AL, UK.; ^4^ Cavendish Laboratory, University of Cambridge, J.J. Thomson Avenue, Cambridge, CB3 0HE, UK.; ^5^ Kebotix Inc., 501 Massachusetts Avenue, Cambridge, MA 02139, USA.; ^6^ Department of Materials Science & Metallurgy, University of Cambridge, 27 Charles Babbage Road, Cambridge, CB3 0FS, UK.; ^7^ Department of Chemistry, Durham University, Durham DH1 3LE, UK.; ^8^ Laboratoire de Physique de l’Ecole normale supérieure, ENS, Université PSL, CNRS, Sorbonne Université, Université Paris Cité, F-75005 Paris, France.; ^9^ Department of Physics, University of Calabria, Via Pietro Bucci, 87036 Rende CS, Italy.; ^10^ School of Physics, Engineering and Technology, University of York, Heslington, York YO10 5DD, UK.; ^11^ SuperSTEM, Daresbury WA4 4AD, UK.; ^12^ School of Chemical and Process Engineering, University of Leeds, Woodhouse Lane, Leeds LS2 9JT, UK.; ^13^ School of Physics and Astronomy, University of Leeds, Woodhouse Lane, Leeds LS2 9JT, UK.; ^14^ School of Chemistry, University of Leeds, Woodhouse Lane, Leeds LS2 9JT, UK.; ^15^ Department of Materials, Royal School of Mines, Imperial College London, London, SW7 2AZ, UK.

## Abstract

Organic semiconductors continue to make substantial performance gains from photovoltaics to electronics. However, understanding how differences in solid-state structure give rise to large differences in energy transport properties remains unresolved. We report that microcrystals of two perylene diimide (PDI) derivatives differing only in their terminal groups [cyclohexyl (CH) and 4-heptyl (ST)] have exciton diffusion coefficients differing by more than two orders of magnitude. Applying state-of-the-art techniques for microcrystal structure determination, we report the crystal structures of CH-PDI and two polymorphs of ST-PDI. Scanning electron diffraction reveals a range of crystallographic defects in ST-PDI microcrystals, attributed to polymorph intergrowths, while electron energy loss spectroscopy links these defects to nanoscale electronic structure changes. Computational modeling demonstrates that rotational disorder explains the difference in exciton diffusion coefficients. Our observations establish the importance of defect-induced orientational disorder as a source of extrinsic energetic disorder, highlighting the need for defect management in organic semiconductor technologies.

## Teaser

Polymorph intergrowths in organic semiconductors are detrimental to exciton diffusion.

## INTRODUCTION

Organic semiconductors hold substantial promise for scalable and processable optoelectronics from organic photovoltaics to light-emitting diodes and organic thin-film transistors. Efficient transport of energy in the form of singlet excitons is central to this effort. However, most organic semiconductors show singlet exciton diffusion lengths between 5 and 50 nm (*1*, *2*), with only a handful of reported systems exhibiting longer diffusion lengths (*3*–*8*). The excitonic states arise from both the molecular structure and intermolecular interactions in the solid state, which are often dominated by π−π interactions for aromatic molecules. Intrinsic energetic disorder is believed to be a key source of reduced charge and energy transport (*9*), including contributions from the electronic structure as well as dynamical effects, i.e., phonon modes (*10*–*14*). However, disorder in the molecular packing, such as crystal defects including grain boundaries, dislocations, stacking faults, and noncrystalline domains, provides a further source of trap states (*15*–*17*) or precludes long-distance energy transport mechanisms available in ordered crystals (*18*, *19*).

Linking energy transport measurements to detailed characterization of structural and electronic properties across length scales is therefore crucial for identifying the ways in which structural defects determine optoelectronic properties. Nanometer-precision energy transport measurements have become possible using femtosecond transient absorption microscopy (fs-TAM), which enables energy transport to be visualized on sub–10 nm length scales with a sub–10 fs temporal resolution (*19*–*21*) by using a pump-probe optical setup to measure the spatial profile of the excited population. High precision in profile localization and shape allows spatiotemporal dynamics to be monitored well below the optical diffraction limit. However, structural characterization from unit cell descriptions of the average crystal structure to microscopic examination of individual nanoscale crystals or defects faces several hurdles, including difficulties in obtaining high-quality single crystals of sufficient size for single-crystal x-ray diffraction, anisotropic crystal growth resulting in considerable preferred orientation in powder x-ray diffraction (PXRD), and extensive polymorphism. Moreover, in electron microscopy–based diffraction and spectroscopy required to access the nanometer length scales, organic crystals are highly susceptible to radiolysis and structural degradation, termed electron beam damage (*22*).

Combining multiple techniques across length scales together with ab initio calculations offers a key route to circumvent these challenges (*23*). Three-dimensional electron diffraction (3D-ED) enables structure determination from nanoscale single crystals but suffers from beam damage as well as nonlinearities in dynamical electron scattering, which often constrain the precision of structures determined from 3D-ED data. However, together with the analysis of PXRD data, the crystal structures can be refined and evaluated further through multitechnique strategies (*23*), including the use of dispersion-corrected density functional theory (DFT-D) calculations and the evaluation of both experimental and calculated solid-state nuclear magnetic resonance (NMR) spectroscopy data. Alternatively, knowledge of the molecular structure enables direct-space structure solution [for example, using the direct-space genetic algorithm (GA) technique (*24*)] to be carried out directly from PXRD and/or 3D-ED data, and the resulting structure can be further validated using periodic DFT-D calculations and/or solid-state NMR data (*23*, *25*).

While these approaches provide knowledge of the unit cell structure and the average atomic positions within the crystal, they are unable to probe individual, nanoscale defects and local disorder that represent a minor fraction of the macroscopic solid but can nevertheless modify its optoelectronic response substantially. Two further techniques are required for (i) nanoscale crystallographic analysis and (ii) correlated nanoscale electronic structure characterization. Recent developments in scanning electron diffraction (SED) (*18*, *26*, *27*) have introduced this variant of four-dimensional scanning transmission electron microscopy (4D-STEM) for low-dose structural analysis in organic solids at a <10-nm spatial resolution. In parallel, electronic structure probes for optical transitions in organic semiconductors using electron energy loss spectroscopy (EELS) have been demonstrated in promising examples with a ~10- to 100-nm spatial resolution (*28*, *29*). In particular, progress in aloof-beam monochromated EELS (*30*–*32*) presents a way to improve the spatially resolved signals with a high energy resolution in spectroscopy at optical transition energies while preserving the unexposed internal structure of the crystal. In aloof EELS, the electron beam is placed outside the crystal to record spectra with reduced radiolytic damage. Consequently, the aloof EELS approach enables diffraction-contrast imaging from the crystal to be acquired subsequently for the purpose of linking crystal structure information with local variations in the valence electronic structure.

**Fig. 1.**
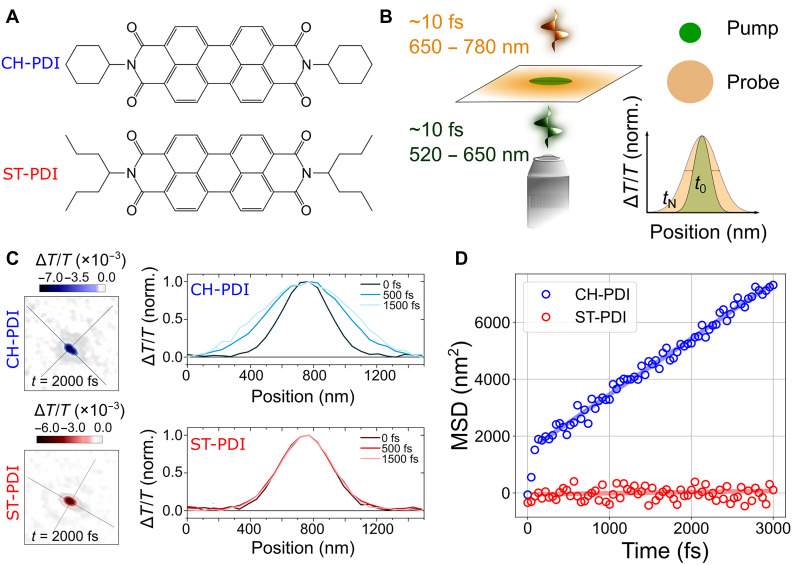
Exciton diffusion measurements for two PDI derivatives. (**A**) Molecular structures of CH-PDI and ST-PDI, in which the PDI molecule is substituted at the imide positions with cyclohexyl (CH) and 4-heptyl [swallow tail (ST)] groups, respectively. (**B**) Schematic of the fs-TAM setup. (**C**) Experimental fs-TAM results for CH-PDI and ST-PDI single nanobelts, depicted as (left) two-dimensional profiles highlighting the relative change in the spatial extent of the signal at a single time delay *t* = 2000 fs and as (right) 1D profiles at a series of time delays along the major axis (long axis of the nanobelts). (**D**) MSD as a function of probe delay time, showing two regions. At short delays, a rapid change is observed for CH-PDI (*19*). The diffusion coefficient characterizing transport is extracted from the longer-time regime. Linear fits are superimposed.

Here, we apply a multitechnique strategy combining energy transport measurements of individual crystals, crystal structure determination, ab initio calculations, and nanoscale diffraction and spectroscopy to understand sizable differences observed in derivatives of perylene diimide (PDI), a major class of n-type organic semiconductors. PDI typically forms X-aggregates (*33*) with a “nanobelt” morphology [defined in the “Femtosecond transient absorption microscopy (fs-TAM)” section] characteristic of anisotropic growth along the π−π stacking direction. We use the term “X-aggregate” to refer to those PDI systems in which the transition dipoles along the long molecular axis of adjacent molecules are distinguishable from the perfectly parallel and cofacial case that is typical for H-aggregates. Accordingly, the molecular transition dipoles interact somewhat less strongly than if they were nearly or exactly parallel. The interaction is, however, far from a null aggregate with transition dipoles perpendicular, sometimes distinguished as a Greek cross (+) aggregate (*34*). In PDI semiconductors, the PDI molecule is often functionalized symmetrically or asymmetrically to modify the crystal structure and optoelectronic properties (*35*). In this work, we focus on two derivatives of PDI that are symmetrically substituted in the imide positions, rather than at positions on the aromatic ring system (sometimes referred to as bay positions). We turn our attention to these symmetrically substituted derivatives as the imide positions are expected to be nodes in the highest occupied molecular orbital and lowest unoccupied molecular orbital, and substitution at these positions generally has little effect on the electronic properties of the isolated molecule (*35*). The two specific derivatives of PDI studied here (Fig. 1A) are as follows: (i) CH-PDI, which contains cyclohexyl (CH) substituents (imide terminal group: N─C_6_H_11_), and (ii) ST-PDI, which contains 4-heptyl [denoted as “swallow-tail” (ST)] substituents [imide terminal group: N─CH(CH_2_CH_2_CH_3_)_2_]. Nanobelts of CH-PDI have been shown previously to exhibit long-range energy transport (*19*). For single-component materials, we expect the primary differences in exciton transport between different derivatives of PDI (such as CH-PDI and ST-PDI) to be related to differences arising in the solid form, specifically the nature of the crystal structure (including the possibility that different polymorphs may exist) and crystal defects or disorder.

We first report nanoscale fs-TAM measurements to establish the differences in energy transport between nanoscale crystals (“nanobelts”) containing CH-PDI and ST-PDI. We then present detailed structure determination of these materials using combined PXRD, 3D-ED, solid-state NMR, and DFT-D, revealing polymorphism in the case of ST-PDI. SED and EELS measurements are then discussed to unravel further nanoscale defects. Last, we apply calculations of optoelectronic properties to understand the role of disordered intermolecular packing and to explain the differences in energy transport between the CH-PDI and ST-PDI materials. In bringing these multiple approaches together, our results reveal insights into the structural determinants of exciton diffusion and, in particular, the role of polymorphic intergrowths and disorder in regulating these properties. The experimental strategies applied in this work to establish the links between polymorphism, structural disorder, and functional properties are anticipated to have much broader applicability to explain poorly understood structure-function relationships for other organic semiconductors, as well as a wide range of other classes of molecular crystals.

## RESULTS

### Femtosecond transient absorption microscopy (fs-TAM)

Nanobelts of CH-PDI and ST-PDI were dispersed by drop-casting from dilute solution for fs-TAM measurements. Because of their characteristic growth anisotropy favoring extension along the π−π stacking direction, both samples exhibit nanobelt morphologies (fig. S1), with lengths on the order of 100 μm (*19*) and widths of ~100 to 150 nm in the case of CH-PDI and ~300 to 500 nm in the case of ST-PDI. The nanobelt thicknesses were estimated from EELS as ~60 nm in the case of CH-PDI and ~120 nm in the case of ST-PDI [fig. S2, table S1, and the “Electron energy loss spectroscopy (EELS)” section in Materials and Methods]. The exciton radius (~5 to 10 nm) is expected to be much smaller than the width and thickness of the nanobelts, and so we do not expect shape or surface effects to dominate the optical response. While recent work has shown that at short times (sub–100 fs), energy transport in the CH-PDI nanobelts exhibits polariton-like exciton transport (*19*), we focus here on the difference in exciton transport characteristics at longer times. Our focus is on how these differences at longer timescales arise from changes in imide functionalization and the resulting differences in crystallographic packing and defects through a comparison between nanobelts prepared from either CH-PDI or ST-PDI.

By applying fs-TAM (*19*), we are able to directly visualize energy transport along the PDI nanobelts with a sub–10 fs time resolution and sub–10 nm spatial localization precision. The principle of this experiment is illustrated in Fig. 1B. A diffraction-limited [~300-nm full width at half maximum (FWHM)] pump pulse of ~10 fs (green in Fig. 1B) is focused using a high–numerical aperture objective onto a single nanobelt, and a wide-field counterpropagating probe pulse of ~10 fs (yellow in Fig. 1B) then tracks the change in size of the spatial transient absorption signal. Subtracting the spatial profile at a given time (*t*_N_) from the spatial profile when there is no delay between the pump and the probe (*t*_0_) allows direct readout of population transport. While recent work has demonstrated that a mismatch in the transient change in the real (*n*) and imaginary (*k*) components of the refractive index can result in an overestimate of the population transport using this method (*36*), by imaging the pump-probe dynamics at 780 nm (the photoinduced absorption of CH-PDI and ST-PDI), where Δ*n*/Δ*k* is small (<1), we are confident that such effects do not play a role here (see also section S2 and fig. S3 for further discussion).

Snapshots of fs-TAM images for the CH-PDI and ST-PDI nanobelts are shown in Fig. 1C at a series of time delays following photoexcitation. Orthogonal solid lines show principal transport axes along the nanobelts. Expansion in the spatial profile, primarily along the long axis of the nanobelt, is observed between *t* = 0 fs and *t* = 2000 fs for the CH-PDI nanobelt, whereas there is essentially no change in the spatial dimensions of the signal for the ST-PDI nanobelt. This expansion is more clearly visualized in plots of the mean-square displacement (MSD) along the long axis of the nanobelts (Fig. 1D). For each pump-probe delay, the FWHM of the spatial pump probe signal can be calculated along the long axis of the nanobelt and converted to a Gaussian standard deviation [σ(*t*)], shown as a function of time in Fig. 1D. In the case of ST-PDI nanobelts, there are very small changes in σ(*t*) from the initial excitation diffraction limit (~300 nm), whereas in CH-PDI nanobelts, σ(*t*) grows from 300 to 340 nm at ~3500 fs following photoexcitation. The rate of σ(*t*) expansion in CH-PDI exhibits two distinct regimes. The behavior in the first region, *t* < 100 fs, has been described by Pandya *et al.* (*19*) as polariton-like, resulting from a species with mixed photonic and excitonic character. Here, we focus only on the second region *t* > 100 fs (Fig. 1D) where the population transport is considered to be purely diffusive and excitonic in nature.

An exciton diffusion coefficient (*D*) is extracted by fitting a straight line to the slope of the MSD-versus-time plot. We carried out measurements for 20 locations on dispersed nanobelts to assess the heterogeneity in the sample (fig. S4). We note that these fs-TAM results were obtained for ST-PDI nanobelts prepared in the same way as for CH-PDI nanobelts (see the “Nanobelt fabrication” section). As we show in the “Structure determination of polymorphs I and II of ST-PDI” section, the ST-PDI nanobelts comprise a mixture of polymorphs I and II in unknown proportions, which we have not found to be distinguishable in optical or electron microscopy without accompanying detailed diffraction analysis. While we have performed microabsorption measurements across more than 200 nanobelts [see also the “Electron energy loss spectroscopy (EELS)” section in Results and the “Femtosecond transient absorption microscopy (fs-TAM)” section in Materials and Methods], these measurements showed only very small changes in the shape of the absorption spectra across the ST-PDI nanobelt sample. Moreover, such measurements are sensitive to factors like interference effects, sample orientation, and polarization, which we were unable to correct for to the level of precision that would be required to separate any such subpopulations on the basis of their absorption spectra. Accordingly, these fs-TAM results represent a population of single-phase and variably intergrown materials, which may explain the skew distribution (fig. S4). Data on the distribution of responses for CH-PDI have been reported previously (*19*). The exciton diffusion coefficient (*D*) is measured here as <0.1 cm^2^ s^−1^ for ST-PDI nanobelts (close to the measurement resolution of our system based on the signal-to-noise ratio) and ~22 cm^2^ s^−1^ for CH-PDI nanobelts (±5 cm^2^ s^−1^ based on sample locations). We therefore establish, through the application of fs-TAM, that ST-PDI and CH-PDI nanobelts have substantially different excitonic transport properties, both in terms of the biphasic response that is observed in CH-PDI but not observed in ST-PDI and in terms of the variation in the exciton diffusion rate by two orders of magnitude. These differences occur despite chemical and morphological similarity between the two systems. These observations have motivated a detailed evaluation of the underpinning differences in crystal structure.

### Structure determination

As the CH-PDI and ST-PDI nanobelts are not suitable for structural characterization using single-crystal x-ray diffraction, crystal structure determination was carried out using a multitechnique strategy involving the analysis of 3D-ED data and PXRD data, augmented by periodic DFT-D calculations and solid-state NMR studies.

#### Structure determination of CH-PDI

From preliminary analysis of 3D-ED data, the crystal structure of CH-PDI was assigned as orthorhombic (*Pna*2_1_; *a* = 22.88 Å, *b* = 7.24 Å, and *c* = 37.61 Å). Starting from these approximate unit cell parameters, profile fitting [using the Le Bail method (*37*)] and unit cell refinement from PXRD data gave a good quality of fit (fig. S5), confirming that the powder sample contained only one crystalline phase [refined unit cell parameters: *a* = 21.1664(26) Å, *b* = 7.07647(23) Å, and *c* = 36.542(4) Å]. From the unit cell volume and consideration of density, it was deduced that there are eight molecules of CH-PDI in the unit cell, with two molecules in the asymmetric unit for space group *Pna*2_1_.

As the PXRD data for CH-PDI are influenced considerably by preferred orientation, structure solution was carried out from 3D-ED data rather than PXRD data (section S4.2 and fig. S6) using the direct-space GA technique (*24*) implemented in the program EAGER [this program was originally developed for structure solution from PXRD data (*38*–*44*) and has recently been adapted for structure solution from 3D-ED data (*23*, *25*, *45*, *46*) using electron scattering factors from Lobato and Van Dyck (*47*)]. The best structure solution from 3D-ED data was used as the initial structural model for Rietveld refinement from PXRD data (section S4.3), which included refinement of parameters to account for preferred orientation (*48*, *49*). Following initial Rietveld refinement, DFT-D geometry optimization was used to establish the optimal orientations of the cyclohexyl substituents relative to the aromatic ring system in the CH-PDI molecules. This optimized structural model was then used in the final Rietveld refinement, which gave a good quality of fit (*R*_p_ = 0.81%, *R*_wp_ = 1.24%; fig. S8) comparable to the quality of fit obtained in profile fitting of the same PXRD dataset (*R*_p_ = 0.53%, *R*_wp_ = 0.74%; fig. S8), with the following final refined unit cell: *a* = 21.168(4) Å, *b* = 7.08303(30) Å, and *c* = 36.661(11) Å. Subjecting the refined structure to DFT-D geometry optimization (with a fixed unit cell) resulted in only minor atomic displacements [fig. S10; root mean square deviation (RMSD) = 0.32 Å for non-H atoms], confirming that the refined structure is very close to a minimum on the energy landscape.

**Fig. 2.**
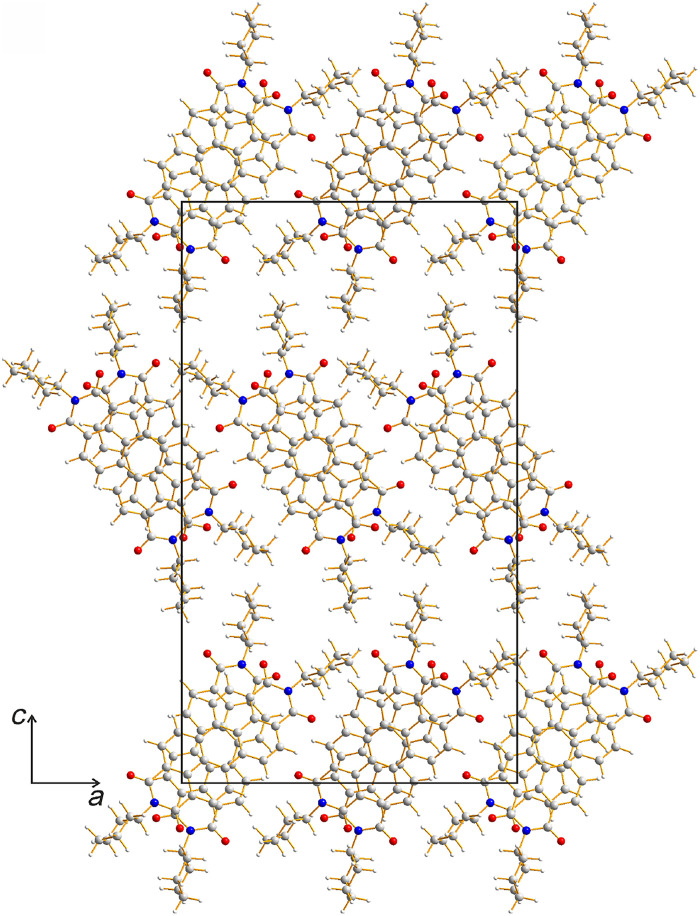
Crystal structure of CH-PDI determined by Rietveld refinement from PXRD data. The structure is viewed along the stacking axis (*b* axis). Carbon atoms are shown in gray, hydrogen atoms are shown in white, nitrogen atoms are shown in blue, and oxygen atoms are shown in red.

The refined structure of CH-PDI (Fig. 2) comprises stacks of CH-PDI molecules (along the *b* axis). The two independent molecules, which alternate along the stack, have very similar conformations (fig. S11) in which the mean plane of each cyclohexyl substituent is close to perpendicular to the aromatic ring system. The molecules are tilted relative to the stacking axis (by 16.8° and 16.9° for the two independent molecules) and alternate between two orientations on moving along the stack. The orientation of the long molecular axis (the intramolecular N···N vector) differs by 38.2° between adjacent molecules. This alternation of molecular orientations allows the aromatic ring systems to form favorable π−π stacking interactions while avoiding unfavorable steric interactions between the cyclohexyl substituents. The perpendicular distance between the planes of the aromatic ring systems of adjacent molecules along the stacks in the refined structure is ~3.39 Å (this distance becomes ~3.37 Å after DFT-D geometry optimization), corresponding to typical values for π−π stacking interactions (*50*). The arrangement of stacks relative to each other is shown in Fig. 2 (see also section S4.4 and figs. S12 and S13). The predominant interactions between adjacent stacks are van der Waals interactions, and substantial electronic interactions exist only within each stack.

#### Structure determination of polymorphs I and II of ST-PDI

In our 3D-ED studies, we observed that different crystallites in the powder sample of ST-PDI gave different 3D-ED patterns, leading to the assignment that the sample contains two polymorphs (denoted as I and II), with triclinic metric symmetry for polymorph I (*a* = 7.58 Å, *b* = 11.65 Å, *c* = 20.08 Å, α = 93.90°, β = 102.50°, and γ = 105.60°) and monoclinic metric symmetry for polymorph II (*a* = 21.22 Å, *b* = 20.50 Å, *c* = 7.61 Å, α = 90°, β = 91.70°, and γ = 90°). This assignment was fully supported by PXRD data for the same sample. Specifically, profile fitting of the PXRD data using the Le Bail method (section S5.1 and fig. S14) based on two phases with initial unit cells corresponding to those determined from the 3D-ED data gave a good quality of fit to the PXRD data (*R*_p_ = 1.04%, *R*_wp_ = 1.36%). The refined unit cells from the PXRD data (at 294 K; table S2) for polymorph I [*a* = 7.4817(5) Å, *b* = 11.3565(7) Å, *c* = 19.3050(16) Å, α = 93.846(6)°, β = 98.650(5)°, and γ = 107.659(6)°] and polymorph II [*a* = 20.7572(18) Å, *b* = 19.6919(12) Å, *c* = 7.6277(3) Å, α = 90°, β = 93.245(6)°, and γ = 90°] are considered more accurate than those determined from the 3D-ED data and were used in subsequent structure solution and refinement calculations from the 3D-ED data.

The 3D-ED data were recorded at cryogenic temperatures (~100 K) to minimize the effects of radiation damage (*22*) to record the highest-quality diffraction data. However, it is recognized that unit cells determined directly from 3D-ED data are less accurate than those determined from PXRD data, and so, it is common practice in structure determination from 3D-ED data to use the unit cell determined for the same material from PXRD data (*23*, *46*, *51*, *52*). As our primary objective here is to apply structural insights to rationalize electronic properties at ambient temperature, we considered that carrying out structure determination from the 3D-ED data while using the unit cell determined from PXRD data at ambient temperature (294 K) would provide the best representation of the crystal structures. We note that this issue only arises in the case of polymorphs I and II of ST-PDI; for CH-PDI, the final structure refinement was carried out from PXRD data recorded at ambient temperature. The validity of this approach is further confirmed by (i) the fact that there is no evidence for the occurrence of any phase transitions in polymorphs I and II of ST-PDI between ambient temperature and 100 K, and (ii) our SED studies [see the “Scanning electron diffraction (SED)” section] demonstrate that the electron diffraction patterns of nanobelts of polymorphs I and II of ST-PDI at ambient temperature are fully consistent with the crystal structures determined using this approach.

Direct-space structure solution was carried out from the 3D-ED data for each polymorph of ST-PDI using EAGER (section S5.2 and fig. S16). For polymorph I, the best structure solution was described by space group $P\bar{1}$, with two half-molecules in the asymmetric unit (each located on a crystallographic inversion center). For polymorph II, the best structure solution (space group *P*2_1_/*c*) has one molecule in the asymmetric unit. Structure refinement from the 3D-ED data was carried out on the basis of the kinematic approximation using SHELXL. The refinements converged with R1 residuals of 0.243 for polymorph I and 0.280 for polymorph II (more details are given in tables S3 and S4).

**Fig. 3.**
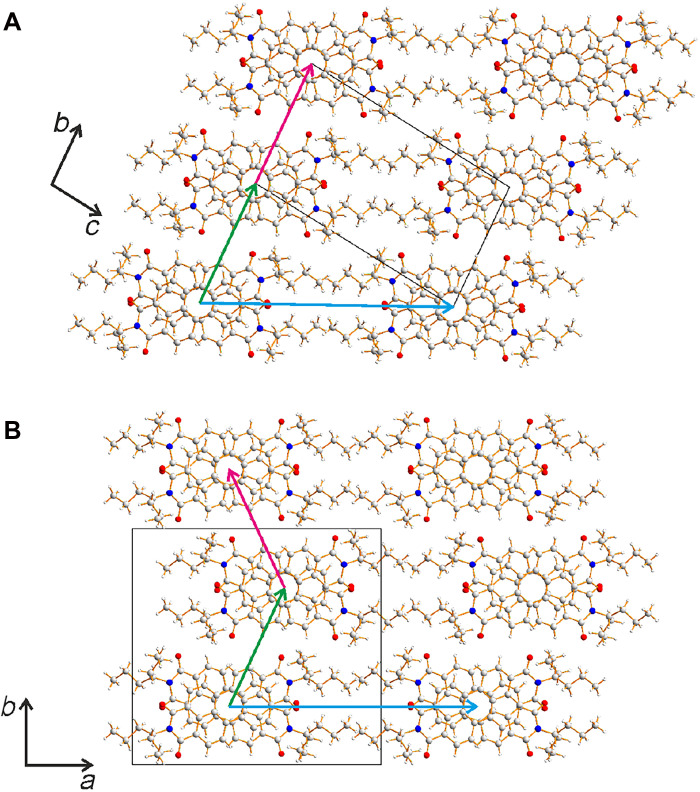
Crystal structures of polymorphs I and II of ST-PDI. Crystal structures of (**A**) polymorph I and (**B**) polymorph II of ST-PDI viewed along the stacking axis in each case. Carbon atoms are shown in gray, hydrogen atoms are shown in white, nitrogen atoms are shown in blue, and oxygen atoms are shown in red. The arrows indicate vectors (in the plane perpendicular to the stacking axes) between neighboring stacks, as discussed in section S5.7.

Figure 3 shows the final refined structures of polymorphs I and II of ST-PDI. DFT-D geometry optimization (with fixed unit cell) leads to only minor atomic displacements for each polymorph (RMSD = 0.11 Å for non-H atoms in each case), confirming that the refined structures are very close to minima on the energy landscape. From these DFT-D calculations, polymorph I is lower in energy than polymorph II by 2.71 kJ mol^−1^ per molecule. After further DFT-D geometry optimization with relaxation of the unit cell (section S5.5 and table S5), polymorph I is still lower in energy but by only 0.35 kJ mol^−1^ per molecule. These differences in energies are within the typical ranges observed for other polymorphic molecular crystals (*53*, *54*).

ST-PDI nanobelts observed in 3D-ED experiments showed a range of features including nanobelts that give high-quality single-crystal electron diffraction data suitable for structure determination, indicating that there are at least some nanobelts with a high degree of ordering (i.e., crystals that are a single polymorph, at least locally). In a subset of the 3D-ED data sets obtained from ST-PDI, lines of diffuse scattering can be observed (fig. S17), suggesting possible disorder in molecular packing in the form of alternating domains such as observed in the intergrowth of polymorphs (*55*). We examine disorder further in the “Scanning electron diffraction (SED)” section.

Experimental solid-state ^13^C NMR data recorded for the powder sample comprising a mixture of polymorphs I and II of ST-PDI are shown in fig. S18 and discussed in more detail in section S5.6. To assist the process of structure validation, solid-state ^13^C NMR spectra calculated using the DFT gauge including projector augmented wave (DFT-GIPAW) methodology (*56*–*59*) in CASTEP (version 21.1.1) (*56*) for the crystal structures of polymorphs I and II are in good agreement (see fig. S19) with the experimental solid-state ^13^C NMR spectrum, which provides additional support for the correctness of the crystal structures. Solid-state ^13^C NMR spectra (fig. S20) were also recorded using the dipolar-dephasing technique (*60*, *61*), which can provide qualitative insights into the occurrence of dynamic processes in organic material (*62*–*67*); the results (see section S5.6) suggest that no substantial dynamic processes (e.g., conformational dynamics of the 4-heptyl groups) occur in polymorph I or polymorph II of ST-PDI at ambient temperature.

**Fig. 4.**
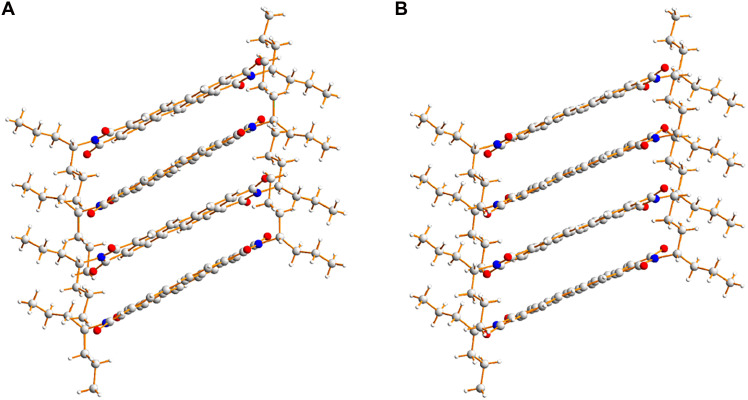
Stacks of ST-PDI molecules, viewed perpendicular to the stacking axis. The molecular stacks in the structures of (**A**) polymorph I and (**B**) polymorph II of ST-PDI. The RMSD values in atomic positions between the stacks in polymorphs I and II are 0.17 Å for all non-H atoms in the molecule (including the 4-heptyl substituents) and 0.10 Å for all non-H atoms in the aromatic ring system. Carbon atoms are shown in gray, hydrogen atoms are shown in white, nitrogen atoms are shown in blue, and oxygen atoms are shown in red.

The crystal structures of polymorphs I and II of ST-PDI (Fig. 3 and fig. S21) are both based on π−π stacking of molecules. In polymorph I, the two independent molecules alternate along the stack (parallel to the *a* axis), with the center of each molecule located on a crystallographic inversion center. Polymorph II has one molecule in the asymmetric unit, with adjacent molecules along the stack (parallel to the *c* axis) related by the *c*-glide operation. While polymorphs I and II have different symmetry properties, the stacks of ST-PDI molecules are essentially isostructural (Fig. 4 and figs. S22 to S24). The ST-PDI molecules are tilted relative to the stacking axis by 22.6° and 23.5° in polymorph I and by 23.3° in polymorph II, and the perpendicular distances between the planes of the aromatic ring systems of adjacent molecules are ~3.44 Å for polymorph I and ~3.50 Å for polymorph II. The 4-heptyl substituents in each polymorph have very similar conformations (fig. S25) in which the two propyl “branches” are oriented above and below the plane of the aromatic ring system, with one branch conformationally more extended than the other.

In each polymorph, the molecules alternate between two orientations along the stack (figs. S23 and S24), with the long molecular axis (the intramolecular N···N vector) differing in orientation by 40.5° for polymorph I and 39.2° for polymorph II. As also observed for CH-PDI, this alternation of molecular orientations allows favorable π−π stacking interactions of the aromatic ring systems of adjacent molecules while avoiding unfavorable steric interactions between the 4-heptyl substituents.

While the individual stacks in polymorphs I and II are essentially isostructural, the arrangement of stacks relative to each other is different, as shown in Fig. 3 (see also section S5.7). Each structure is described in terms of rows of stacks aligned parallel to the long axis of the cross-sectional shape of the stack (horizontal in Fig. 3; corresponding to the projection of unit cell vector **b** + **c** in polymorph I and the projection of unit cell vector **a** in polymorph II). Relative to a given row, the position of the adjacent row (moving vertically upward in Fig. 3) is essentially identical in each polymorph. However, the position of the next row differs for polymorphs I and II, with the displacement relative to the row below continuing in the same direction in polymorph I but in the opposite direction in polymorph II. As a result, the positional relationship between adjacent rows of stacks propagates in the same direction throughout the structure of polymorph I but alternates in a zigzag manner in polymorph II.

Notably, one of the few previously reported crystal structures of a PDI derivative, *N*,*N*′-bis(3-pentyl) PDI (*68*), which contains a shorter branched alkyl chain at the imide positions, exhibits similar unidirectional row-like packing of stacks as in polymorph I of ST-PDI. Another *N*,*N*′-bis(3-pentyl) derivative of PDI, which also contains a nitro substituent on the aromatic ring system (i.e., on a bay position), denoted as nitro-PDI (*69*), instead shows zigzag packing of stacks as in polymorph II of ST-PDI. In contrast, the CH-PDI structure does not exhibit analogous end-to-end rows of stacks (Fig. 2). However, in the CH-PDI structure and in the reported *N*,*N*′-bis(3-pentyl) PDI structure (*68*), the direction of tilting of the molecular planes relative to the stacking axis alternates between adjacent stacks (fig. S26, A and D). In contrast, in both polymorphs I and II of ST-PDI, the direction of tilting of the molecular planes relative to the stacking axis is identical for all stacks (fig. S26, B and C). For the reported nitro-PDI structure (*69*), on the other hand, the direction of tilting of the molecular planes is identical for adjacent stacks in one direction but alternates between adjacent stacks in a different direction (fig. S26E). Clearly, these comparisons highlight the diverse packing possibilities between the π−π stacks in the crystal structures of otherwise similar derivatives of the PDI molecule. Nevertheless, as a defining feature of the crystal structures of these PDI derivatives, the main interactions between adjacent stacks in polymorphs I and II of ST-PDI are van der Waals interactions, and substantial electronic interactions exist only within each stack.

### Scanning electron diffraction (SED)

**Fig. 5.**
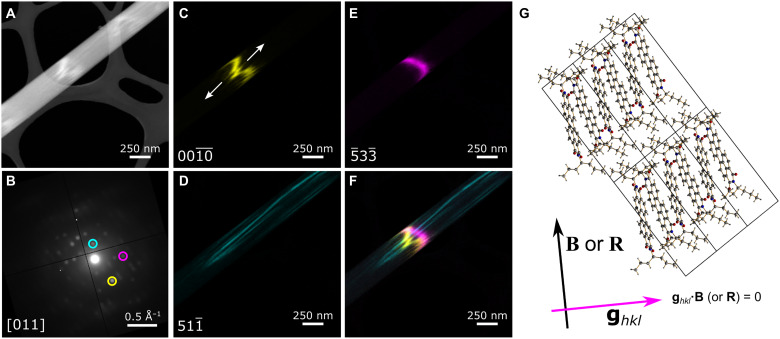
Linear defects observed along the long axis of an ST-PDI nanobelt by SED. (**A**) Annular dark field STEM overview showing bright diffraction contrast at positions where crystal bending causes the exact Bragg condition to be satisfied (bend contour contrast). (**B**) Average diffraction pattern corresponding to the field of view in (A). The diffraction pattern was indexed to the [011] zone axis of polymorph II of ST-PDI. Three diffraction spots selected for VDF imaging are marked with circles. (**C** to **E**) VDF images corresponding to the selected diffraction spots. The intensity recorded at the virtual aperture positions is plotted at each probe position in the scan. The Miller indices *hkl* are given in the lower left corner for each selected diffraction spot. White arrows in (C) mark opposing tilts on either side of the defect. (**F**) Color overlay showing all three single-spot VDF images. (**G**) Crystal structure of polymorph II of ST-PDI viewed in the same orientation as the diffraction pattern in (B). Carbon atoms are shown in gray, hydrogen atoms are shown in white, nitrogen atoms are shown in blue, and oxygen atoms are shown in red. A single layer of molecules is shown for visual clarity. The Burgers vector determined from the bend contour with no break (E) corresponds to a direction approximately aligned to the long molecular axis.

To examine local deviations from perfect crystalline packing, we carried out low-dose SED (fluence <16 e^−^ Å^−2^), in which a two-dimensional electron diffraction pattern is recorded at every position in a scanned area of the sample, offering spatially resolved diffraction measurements (<10-nm spatial resolution). Such measurements support the analysis of dislocations (*70*), defect domains (*71*), and other disorder modes (*72*) in beam-sensitive crystals. SED observations revealed a variety of linear defects in ST-PDI nanobelts. Figure 5 presents an analysis of such linear defects aligned along the long axis of an ST-PDI nanobelt. The average diffraction pattern for the nanobelt (Fig. 5B) confirms that the nanobelt is a single crystal with the π−π stacking direction along the long axis of the nanobelt, as observed for all PDI nanobelts. Figure S27 presents additional examples of indexed SED patterns for nanobelts of both polymorphs I and II of ST-PDI.

The nanobelt in Fig. 5 was indexed to the [011] zone axis of polymorph II of ST-PDI. Diffraction contrast (i.e., intensity variation due to varying diffraction conditions across a crystal) primarily arises from bend contours in the nanobelt. When a crystal bends, planes located at a particular position along the bent crystal will be brought to the exact Bragg condition; as such, bend contours can be considered as markers of where on the sample (in space) a set of planes *hkl* satisfy the Bragg condition. These planes in turn correspond to a spot in the SED pattern, taken as a diffraction vector **g***_hkl_* from the center of the pattern. In SED, diffraction intensity recorded at a particular spot can be extracted to reconstruct a so-called virtual dark field (VDF) image for a selected **g***_hkl_* to capture its corresponding bend contour or other diffraction contrast. In this case, the bend contour for the planes $\left(00\bar{10}\right)$ shows an abruptly varying intensity distribution (Fig. 5C), which exhibits displacements on crossing linear features running along the long axis of the nanobelt visible in the VDF image formed from the planes $\left(51\bar{1}\right)$ (Fig. 5, D and F).

Dislocations, a key type of linear defect, are expected to induce abrupt, local changes in orientation for in-plane **g***_hkl_* that do not satisfy the condition **g***_hkl_*·**B** = 0 for a Burgers vector **B** denoting the single, characteristic displacement direction for the defect. For dislocations with a screw component, the tilting of planes near the dislocation core produces a characteristic feathering of the bend contour on crossing the dislocation line (*70*), as observed in Fig. 5C. Planar defects such as stacking faults can also exhibit oscillatory diffraction contrast with an analogous characteristic displacement vector **R** (*73*), likewise with visibility decreasing at **g***_hkl_*·**R** = 0. The feathering observed (Fig. 5C, white arrows) arises from planes tilting in opposite directions on either side of the defect and is a hallmark of dislocations with a screw component, indicating that dislocations contribute to the observed diffraction contrast. Dislocations and stacking faults can also readily coexist in molecular crystalline materials (*74*). Here, the planes $\left(\bar{5}3\bar{3}\right)$ show no break in the corresponding bend contour (Fig. 5, E and F), enabling the determination of the Burgers or displacement vector along the long axis of the ST-PDI molecules (Fig. 5G). This vector is neither aligned parallel to nor aligned perpendicular to the linear contrast features along the nanobelt, suggesting mixed dislocation character (i.e., one with both screw and edge components and consistent with the observed feathering).

This defect analysis agrees with the geometric requirements for whole-molecule displacements, specifically, to match the inclination of the molecules relative to the stack axis while also preserving the terminal group interactions and the resulting relative alignment between stacks at locations in the crystal far away from the defect (such as away from a dislocation core). In a dislocation, such a displacement necessarily results in an edge component displacement perpendicular to the stack (to introduce an additional stack of molecules) and a screw component displacement parallel to the stack axis (to offset the displaced stacks along the stack axis to retain the interstack arrangement of interacting terminal groups, an alignment that would be altered with a displacement purely perpendicular to the stack axis resulting from the inclination of the molecular plane relative to the stack axis). Local, transient mis-stacking during growth (between unidirectional packing of stacks in polymorph I and the zigzag arrangement of stacks in polymorph II) would produce defects consistent with these observations. Figure S28 depicts a schematic overview of how a mixed dislocation would accommodate a small number of unidirectional stacks as in polymorph I within a predominant zigzag packing sequence typical of polymorph II. Notably, the resulting mis-stacking layer appears along the b* axis of the polymorph II crystal structure, consistent also with the diffuse scattering lines observed in some 3D-ED datasets for polymorph II of ST-PDI (fig. S17). Crystals containing polymorph intergrowths exhibit diffuse streaking in electron diffraction parallel to, but not along, the stacking direction (*55*, *75*). Together, these diffraction analyses (Fig. 5, fig. S17 and fig. S28) point strongly to defects arising from mis-stacking in ST-PDI nanobelts.

**Fig. 6.**
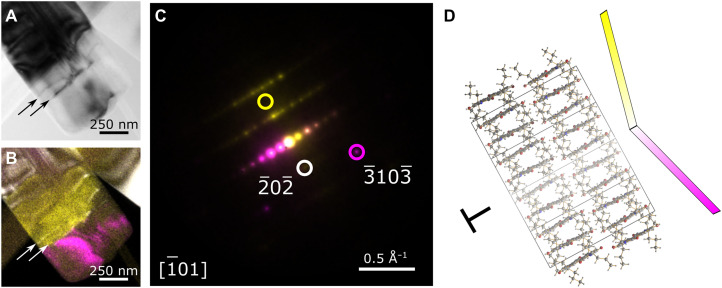
Linear defects across ST-PDI nanobelts. (**A**) Low-angle diffraction contrast image extracted from a SED dataset showing bright-field diffraction contrast (linear defects are dark on a white background). The arrows mark two linear defect bands. (**B**) Color overlay of VDF images constructed from the diffraction spots marked in the corresponding diffraction patterns shown in (C). White arrows mark the bright defect lines corresponding to the dark defect bands in (A). (**C**) Two overlaid electron diffraction patterns taken from (yellow) the left and (magenta) right of the defect bands. Circles mark three selected diffraction spots selected for VDF imaging. The diffraction pattern was indexed to the unit cell of polymorph II of ST-PDI viewed along $\left[\bar{1}01\right]$. (**D**) Crystal structure of polymorph II of ST-PDI viewed in the same orientation as the diffraction pattern in (C), showing a schematic illustration of the abrupt orientation change at the linear defects, characteristic of an edge dislocation. Carbon atoms are shown in gray, hydrogen atoms are shown in white, nitrogen atoms are shown in blue, and oxygen atoms are shown in red.

Further linear defect contrast extending partly or completely across the ST-PDI nanobelts is also observed. Figure 6 presents an example with a high density of these defects visible in a virtual bright-field image (Fig. 6A) as well as in VDF images from selected spots in the average diffraction pattern (Fig. 6, B and C). The defect line nearest the end of this nanobelt coincides with an abrupt change in orientation of the crystal [marked by yellow and magenta in Fig. 6 (B and C)]. This type of abrupt bend (illustrated in Fig. 6D), as outlined in two constituent diffraction patterns slightly misoriented but indexable to the $\left[\bar{1}01\right]$ zone axis orientation for polymorph II of ST-PDI, is typical of edge dislocation characteristics (*76*). Figure S29 presents a further example of a short linear defect at the edge of a nanobelt exhibiting diffuse diffraction at the defect while preserving π−π stacking, suggesting local loss of coherent ordering within the stacks. Together, the along-axis and cross-axis linear defects suggest that a variety of linear defects can be accommodated in the ST-PDI structures.

These defects require considerable deviations from ordered molecular packing, and rotated and misoriented molecules are expected to arise in the vicinity of dislocations in molecular crystals (*77*, *78*). Because of the altered intermolecular spacings produced by the insertion or removal of molecules at the dislocation core, the strained local arrangement of molecules extending into the surrounding volume around the core will necessarily undergo a reconstruction relative to the average crystal structure (the unit cell). Moreover, the polymorphic intergrowth–induced effects, contributing to diffuse scattering (fig. S17) and diffraction contrast along and across the full width of the nanobelts (Fig. 5), are of sufficient density to overlap with the length scales of exciton transport (such as the exciton diffusion signatures across many tens of nanometers observed in CH-PDI; Fig. 1). Notably, defect-associated intergrowths and disorder can be understood to occur stochastically rather than uniformly during growth, giving rise not only to a variety of defects (Figs. 5 and 6) but also to a range of defect densities within the population of nanobelts. The observation of these crystallographic structural changes in ST-PDI accordingly invites further examination of the local modification of the electronic structure by such defects given the importance of intermolecular interactions in the solid in determining excitonic properties.

### Electron energy loss spectroscopy (EELS)

To probe the nanoscale valence electronic structure, we turned to EELS, as high-energy electrons can record valence excitations with a nanometer spatial resolution (*28*). For the PDI nanobelts, we explored a variety of scan strategies to determine suitable electron fluence and signal-to-noise characteristics for acquiring EELS from PDI nanobelts at optical and vibrational transition energies. Scans across the width of the nanobelts were found to result in notable changes in the EEL spectra after just one exposure (figs. S30 to S32), showing notably reduced intensity of the C─H stretch (~0.4 eV) and reduced intensity at 2 to 3 eV and at 6 eV. Line profiles recorded in an aloof beam geometry with the beam immediately outside the nanobelt, however, enabled spectra to be recorded continuously over long distances in a single direction. This direction, along the nanobelt, coincides with the exciton diffusion direction. These spectra did not show signs of substantial damage accumulation during the scan, as evidenced by the enhanced signal-to-noise ratio in average spectra relative to the spectra from individual positions in such scans along the nanobelt edge. Similar scans were also able to recover vibrational transitions (fig. S33).

**Fig. 7.**
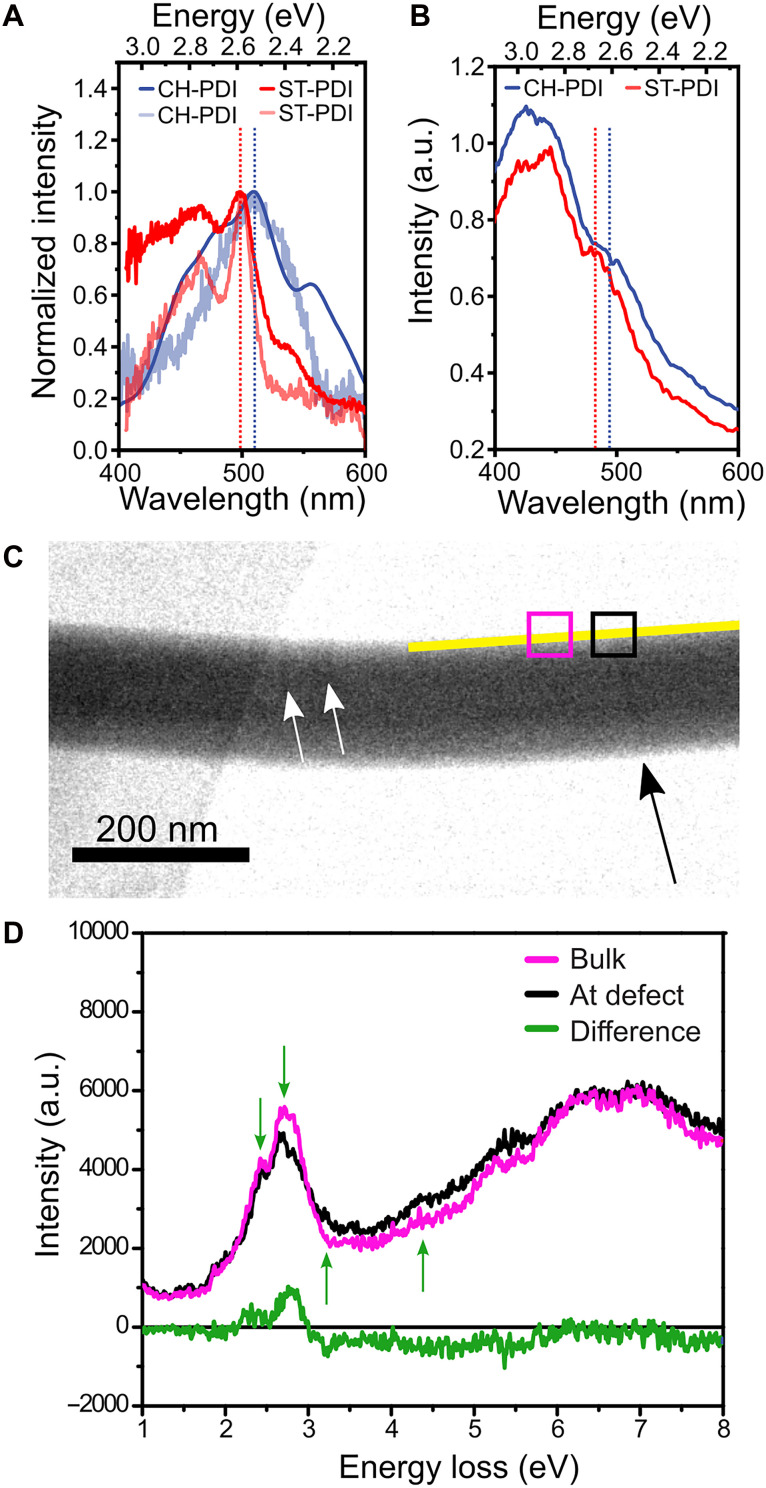
Microabsorption and energy loss spectra at optical energies. (**A**) Single-nanobelt optical spectra acquired for CH-PDI and ST-PDI. The solid and light red lines show two different spectra from different ST-PDI nanobelts. The light blue spectrum shows a single-nanobelt spectrum from a CH-PDI nanobelt. The solid blue line shows an average over many single-nanobelt spectra from CH-PDI. While features at wavelengths greater than 500 nm were inconsistently recorded, the consistent peaks at ~500 nm are marked with dotted blue and red lines for CH-PDI and ST-PDI, respectively. (**B**) EEL spectra from single CH-PDI and ST-PDI nanobelts, with features at ~500 nm marked with dotted blue and red lines for CH-PDI and ST-PDI, respectively. a.u., arbitrary units. (**C**) BF-STEM micrograph of a single ST-PDI nanobelt. The yellow line marks the line profile acquired before the BF-STEM image. EELS data were acquired before slow-scan BF-STEM images to ensure that EELS probed the nanobelts without additional damage from electron beam exposure. The black arrow marks a linear defect band. The white arrows show two further bands analyzed in fig. S34. Selected area spectra are shown for two positions: (magenta) away from the defect and (black) at the defect. (**D**) Corresponding spectra and difference (green) highlighting changes in the spectra away from and at the defect band. Green arrows mark energies showing decreasing or increasing intensity at the defect.

To establish whether the EEL spectra reliably capture optical transition information, Fig. 7A first shows a comparison of microabsorption spectra from single nanobelts, with EEL spectra from CH-PDI and ST-PDI shown in Fig. 7B. The microabsorption spectra show a dominant peak at ~500 nm for both CH-PDI and ST-PDI as well as additional features at lower and higher energies. These additional features are not identically reproduced between different nanobelts of the same material because of their weaker signal (i.e., these weaker spectral features vary between measurements from several different CH-PDI nanobelts and also vary between different ST-PDI nanobelts). Critically, there is a small difference in energy of the most intense peak in the spectra for CH-PDI and ST-PDI. This peak shift is reproduced in the EEL spectra for CH-PDI and ST-PDI (with higher absolute energies observed in EELS, a characteristic of the loss function probed in EELS), providing further confirmation that the EEL spectra here probe similar processes to absorption spectroscopy of the singlet exciton transitions.

Similar line defects to those imaged using SED are also visible in bright-field STEM (BF-STEM) images of the ST-PDI nanobelts (Fig. 7C). BF-STEM (as in SED VDFs) likewise shows diffraction contrast. BF-STEM imaging records fluctuations in the intensity of the set of electrons at near-zero deflection from the incident beam direction (referred to as the direct beam disk in STEM) as a result of intensity scattered to higher angles, predominantly due to diffraction in crystalline materials. Crucially, while the BF-STEM images still exhibit considerable noise, these required slow beam scanning. As such, BF-STEM images were acquired after STEM-EELS to avoid introducing any extensive alteration or damage to the sample from the BF-STEM acquisition ahead of EELS acquisitions at the nanobelt edges. The retention of diffraction contrast in BF-STEM after EELS offered further corroboration that the EELS just outside the nanobelts (aloof EELS) did not lead to widespread damage in the unscanned regions of the nanobelt.

We were therefore able to correlate EELS signals and BF-STEM images from identical nanobelts. Changes in the EEL spectra in the immediate vicinity of the line defects were first identified using independent component analysis (ICA) applied to the spectrum images. This multivariate statistical or machine learning approach reduces the subjectivity associated with manual inspection of the data. We used ICA to highlight regions where changes in the signal are pronounced (fig. S34) without attempting to interpret further the spectral features produced alongside the spatial features. Instead, we returned to the as-acquired EELS data and integrated the spectra from regions at, and away from, the areas identified by ICA (Fig. 7D) to examine the changes in the spectral signature. Across multiple examples (Fig. 7D and fig. S34), we observed a similar local change at the defect lines in the peaks at the exciton energies. At the defects, as highlighted in the difference spectra in Fig. 7D, the EEL spectra show reduced intensity at the singlet exciton transitions at ~2.5 eV and a simultaneously increased probability of inelastic scattering at ultraviolet energies (3.5 to 5 eV). These modifications of the histogram of singlet exciton transitions recorded in EELS serve to link the observed nanoscale structural defects to changes in the local electronic structure.

### Ab initio modeling of electronic excitation spectra and exciton diffusion

To unravel the role of crystal packing, particularly packing disorder at the molecular scale, in the energy diffusion within the nanobelts, we constructed a tight-binding exciton transport model. This model assumes the localization of electron wave functions on individual molecules and the interaction between the excitonic states through Förster coupling (*79*). The structures of isolated CH-PDI and ST-PDI molecules were first optimized, and the lowest 10 singlet electronic transitions, the corresponding transition densities, the transition dipoles, and the oscillator strengths were computed using the time-dependent DFT (TD-DFT) framework. Table S6 summarizes the computed electronic transitions and the corresponding oscillator strengths. The relative differences in energy and oscillator strength for the lowest electronic excitation in CH-PDI and ST-PDI molecules are both below 1%. These variations were therefore neglected in the exciton transport model. The energy gap between the lowest electronic excitation and the next higher excitation in both molecules is ~0.75 eV, which is considerably larger than the Förster intermolecular interaction. Consequently, the excitonic model was constrained to the lowest-energy singlet electronic excitations.

We next constructed an excitonic Hamiltonian for molecular slabs, representing a section of the crystal structure comprising *n_x_* × *n_y_* stacks with *m* molecules along the π−π stacking direction. The slabs were constructed from the DFT-D geometry–optimized crystal structures. For intermolecular exciton transfer, we primarily focused on Förster coupling while qualitatively estimating the contribution of charge transfer (CT)–mediated terms (*80*). While the former interaction is of long range and involves couplings between more distant molecules, the latter is limited to nearest neighbors resulting from direct electronic overlap of molecular orbitals and is highly sensitive to the electronic structure. The Förster interaction between the nearest-neighbor molecules was computed using molecular transition densities, while interactions involving more distant molecules were approximated using the extended dipole model (*79*). Within this framework, the differences between the Hamiltonians of CH-PDI slabs and ST-PDI slabs arise from two main factors: (i) variations in the crystal packing and (ii) the degree of structural disorder.

In all three crystal structures, i.e., CH-PDI and polymorphs I and II of ST-PDI, the strongest Förster interactions occur between adjacent molecules in a given stack defined by π−π interactions. The interaction strength between the nearest molecules in different stacks is ~10 to 20% of the intermolecular coupling within a single stack (table S7). The nearest-neighbor coupling in CH-PDI is about 20% stronger than in the ST-PDI polymorphs owing to the smaller intermolecular distance; specifically, for slabs based on the DFT-D geometry–optimized structures, the perpendicular distances between the aromatic ring systems of adjacent molecules along each stack are 3.37 Å for CH-PDI, 3.42 Å for polymorph I of ST-PDI, and 3.46 Å for polymorph II of ST-PDI. These distances follow the same trend as the values determined from the refined crystal structures of 3.39 Å for CH-PDI, 3.44 Å for polymorph I of ST-PDI, and 3.50 Å for polymorph II of ST-PDI.

For the CT-mediated coupling, we adopted the model developed previously (*80*) assuming a large gap between the CT state and the Frenkel exciton states. The hole transfer integrals, $t_{h}$, have opposite signs for CH-PDI compared to polymorphs I and II of ST-PDI (table S8 and fig. S35), which we associated with a slightly different relative orientation of neighboring molecules along the stacks. Then, the coupling terms are determined by the splitting between the CT and the Frenkel exciton states, $\Delta E=E_{\text{CT}}-E_{F}$, which is difficult to compute using ab initio models (*81*). Although it is generally accepted that the CT state lies within a few hundred milli–electron volts of the Frenkel exciton state, the sign of the shift is debated: While DFT calculations for dimers frequently predict negative splitting (*82*), placing the CT state below the Frenkel exciton state, direct fits to experimental vibronic spectra using microscopic models with ΔE as an adjustable parameter typically yield positive values (*80*, *83*).

We confirmed that DFT calculations of dimer exciton states at the B3LYP/def2-SVP level yield a relatively large negative value of ΔE=−390 meV (section S9.1). In this case, the CT-mediated coupling amplifies the difference between the coupling strengths of CH-PDI and ST-PDI and leads to a larger difference in the diffusion coefficients. However, the contribution remains relatively small, ~5 to 10% of the nearest-neighbor Förster coupling term. Direct fitting of the vibronic spectra in our case is complicated by substantial sample-to-sample spectral variation. Therefore, to obtain an order-of-magnitude estimate of the coupling, we used ΔE=150 meV (*80*). In this case, the CT-mediated contribution to exciton coupling is on the order of 15 to 20% of the nearest-neighbor term and partially compensates the difference between the coupling terms (and diffusion coefficients) of CH-PDI and ST-PDI, keeping the Förster interaction as a leading term controlling the exciton transport. These interactions shape the nanobelt spectra: Oblique aggregation of molecules along the π−π stacks leads to the formation of a stronger, blue-shifted feature (H-aggregate–like behavior), while weaker, red-shifted features remain optically allowed. Additional differences in the spectra arise from differences in molecular packing in the crystal structures (both within and between stacks).

**Fig. 8.**
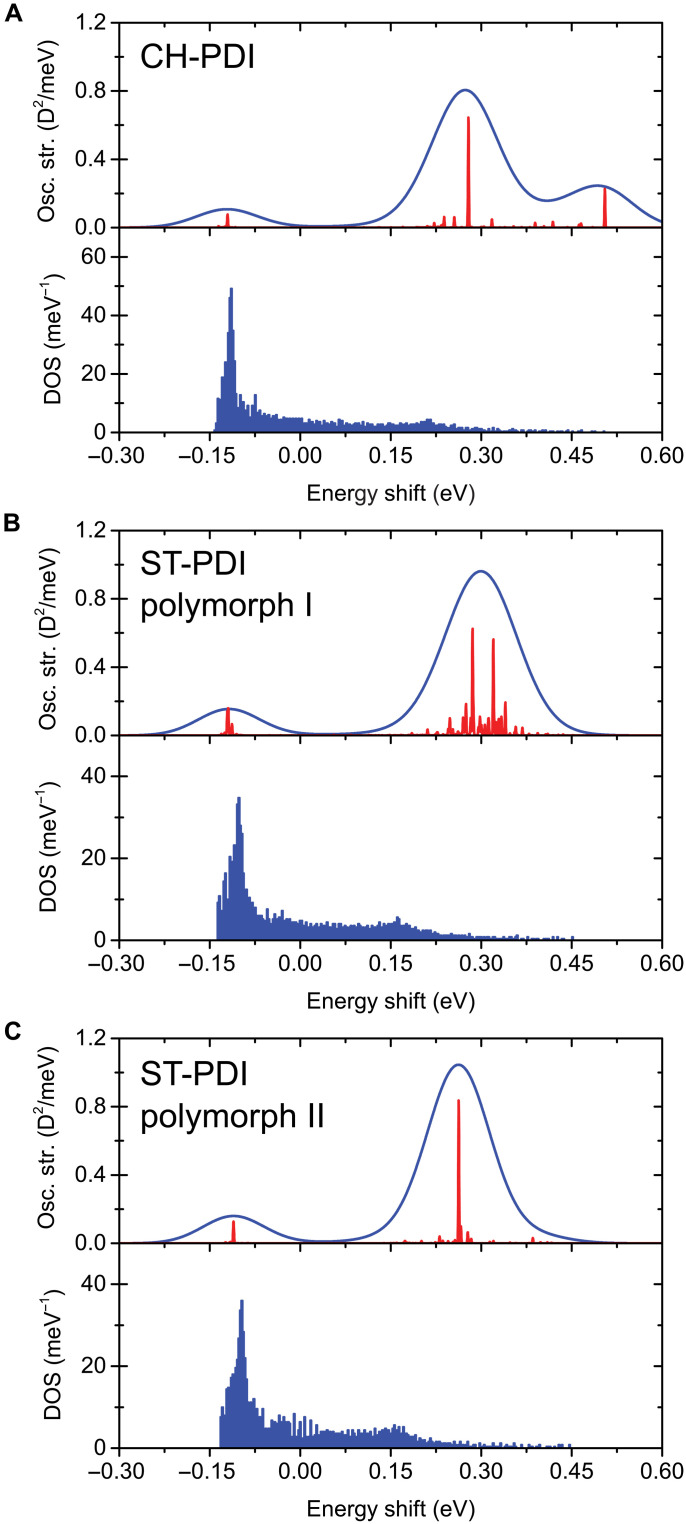
Computed electronic excitation spectra. The spectra are represented by the oscillator strength density and excitonic densities of states of slabs composed of 2160 molecules (6 by 6 stacks with 60 molecules along the π−π stacking direction): (**A**) CH-PDI, (**B**) polymorph I of ST-PDI, and (**C**) polymorph II of ST-PDI. DOS, density of states. The blue envelope line in the excitation spectra corresponds to a 50-meV Gaussian broadening (added to approximate experimental spectra), and the red peaks correspond to transition frequencies. The energy shifts are defined relative to the excitation frequency of an isolated molecule.

Figure 8 shows computed electronic excitation spectra (see fig. S36 for computed vibronic spectra) and the corresponding excitonic densities of states of 3D slabs composed of 2160 molecules (6 by 6 stacks with each stack comprising 60 molecules along the π−π stacking direction). As expected for X-aggregation, two groups of transitions can be identified consistently. Red-shifted transitions are shown as negative energy shifts, and blue-shifted transitions are shown as positive energy shifts. In all three structures (the slabs based on CH-PDI and polymorphs I and II of ST-PDI), the highest density of excitonic states is concentrated near the lower-energy edge. While some of these states are optically bright, the lowest-energy states remain dark. The interaction between the stacks substantially influences the structure of the blue-shifted peak. Notably, in the CH-PDI slab, the highest energy level is optically bright.

Previously, we have shown that the microscopic exciton transport model can reasonably describe diffusion of excitons in CH-PDI (*19*), assuming a perfect crystal packing and moderate thermal fluctuation noise. Here, we assess whether the disorder in molecular orientation, specifically disorder within the π−π stacks, can slow down exciton diffusion. Because of the strong intermolecular coupling within the π−π stacks, excitons in nanobelts propagate along the stacks ~10 times faster, compared to spreading between different stacks. The relative orientation of molecules in the stacks modulates the coupling and, consequently, the diffusion coefficient.

**Fig. 9.**
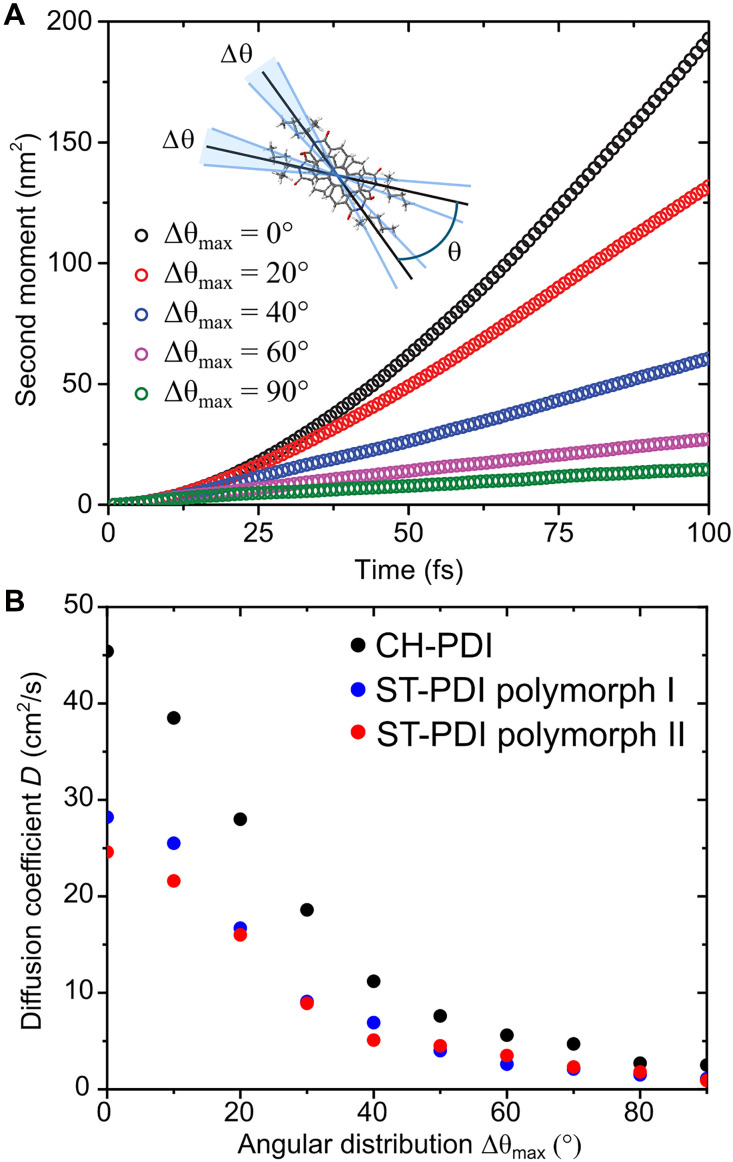
Computational modeling of effects of disorder. (**A**) Computed exciton second moment along the π−π stacks for polymorph I of ST-PDI as a function of time and (**B**) exciton diffusion coefficients along the stacks of CH-PDI and polymorphs I and II of ST-PDI for different levels of angular disorder.

To evaluate the effect of angular disorder, exciton transport was computed for quasi-1D chains on the basis of the structures of CH-PDI and polymorphs I and II of ST-PDI, composed of 1200 molecules (2 by 2 stacks with 300 molecules along the π−π stacking direction). Figure 9 shows the computed second moments and diffusion coefficients for different values of the angular distribution Δθ. Here, Δθ is drawn from a random distribution up to a maximum value Δθ_max_, with values of Δθ_max_ in the range of 0° to 90° (see the inset of Fig. 9A for the definition; see also table S9). The molecules were kept at a fixed intermolecular distance along the π−π stacking axis and were rotated in the plane of the aromatic ring system. While unphysical rotations (for example, involving steric clashes of cyclohexyl or 4-heptyl substituents on adjacent molecules) can occur within this model, the approach nevertheless provides insights into the role and consequences of angular disorder. Our model does not account for the polariton formation on short timescales; therefore, the initial quadratic time dependence of the second moment is due to exciton ballistic propagation. The ballistic transport is converted into diffusion on tens of femtoseconds because of thermal fluctuations.

For the ideal crystal packing with an angular distribution of Δθ_max_ = 0°, exciton diffusion in ST-PDI nanobelts is slower than in CH-PDI nanobelts by only a factor of ~2. However, disorder in the relative orientations of molecules in the π−π stacks (i.e., a distribution of relative orientations of π−π stacked molecules) that is modulated by the functional groups (CH versus ST) can substantially alter transport. Specifically, the results indicate that the angular variation in molecular packing within stacks alone can modulate the diffusion coefficient by up to two orders of magnitude. Angular variations in anthracene (*78*) and paraffin (*77*) molecules have been modeled to exceed 10 to 20° at dislocation cores, and helical arrangements of PDI molecules (sequential rather than alternating orientations of the intramolecular N···N vectors) have been identified in PDI packing in solvated aggregates of other derivatives of PDI (*84*, *85*). Such deviations (i.e., helical motifs rather than the alternating orientations in the crystal structures of polymorphs I and II of ST-PDI) would amount to 40° to 80° misrotations for ST-PDI molecules. We posit that such disorder, and the concomitant reduction in exciton diffusion, arises in ST-PDI crystals resulting from the greater flexibility of the terminal 4-heptyl substituents; accessible polymorphism and associated local, short-range intergrowth of the typical packing of π−π stacks of the two polymorphs during growth (fig. S28); or a combination thereof. Molecular displacements (and associated strain) will be minimized in such dislocation core structures through orientational and conformational disordering to fill gaps arising from whole-molecule displacements or to avoid steric clashes. These findings prompt substantial advances beyond current capabilities for modeling of dislocation core structures and of exciton diffusion characteristics at such extended defects.

## DISCUSSION

By combining fs-TAM measurements of exciton diffusion characteristics in CH-PDI and ST-PDI nanobelts with a battery of structural and spectroscopic probes and ab initio modeling, we have identified that extrinsic molecular packing disorder leads to a reduction by up to two orders of magnitude in the exciton diffusion in ST-PDI compared to CH-PDI. We first determined the crystal structures of these nanocrystalline materials to high precision through the application of a multitechnique strategy, involving structure determination by analysis of both PXRD data and 3D-ED data, together with the use of periodic DFT-D calculations and solid-state NMR data to facilitate the process of crystal structure validation.

With SED analysis, the crystal structures revealed dislocations along and across the ST-PDI nanobelts, in contrast to the defect-free characteristics of CH-PDI nanobelts. EELS at optical transition energies at, and away from, these defects in ST-PDI nanobelts indicates that they modulate the valence electronic structure in their immediate (nanoscale) vicinity. Whereas in TD-DFT calculations, the intrinsic differences arising from the molecular packing in the CH-PDI and ST-PDI crystal structures can explain only a change in the exciton diffusion characteristics by a factor of 2, rotational disorder within stacks of molecules coupled by π−π interactions offers an explanation for the reduction by two orders of magnitude in the exciton diffusion coefficient for ST-PDI nanobelts. The elevated defect density in ST-PDI nanobelts, characterized by preserved π−π stacking interactions while deviating from the molecular packing arrangements in the average crystal structures, outlines a microscopic structural disorder mechanism deleterious to exciton diffusion.

It should be noted that the CT-mediated coupling, which we qualitatively analyzed in the present study, is highly sensitive to the relative orientation of the molecules and can even change sign because of a small relative rotation between neighboring molecules. Recent studies have suggested that the gap between the CT and Frenkel exciton states could be relatively small (*86*, *87*). We believe that this regime warrants a separate, more detailed study.

While we have not yet explored the formation of ST-PDI nanobelts under specific polymorph control, this work raises the prospect of engineering polymorph formation in future studies, for example, by systematic variation of temperature (*88*) and/or crystallization solvent to alter the kinetics of crystal growth, which may selectively direct the formation of ST-PDI nanobelts with enhanced polymorph control. Moreover, the work also invites further exploration of modified crystallization conditions for CH-PDI and other terminally substituted PDI derivatives as a basis for the discovery of new polymorphs of these materials, as well as to examine whether the polymorphic intergrowths observed for ST-PDI are a special case or a more general phenomenon for PDI materials. These findings invite the renewed exploration, driven by multiscale characterization, of the role of crystallographic defects in organic semiconductors and molecular crystals more widely.

## MATERIALS AND METHODS

### Synthesis

Solvents and reagents were purchased from commercial suppliers and used as received. Flash chromatography was carried out using Acros ultrapure silica gel (40 to 60 μm). Analytical thin-layer chromatography (TLC) was carried out on cut aluminum-backed silica gel 60 F254 plates (Merck) and followed using ultraviolet irradiation (λ_max_ = 254 or 365 nm) or permanganate staining. Solution phase ^1^H NMR spectra were recorded on a Bruker Ultrashield 400-MHz Smart Probe Spectrometer or a Bruker Avance 500-MHz Cryo Ultrashield. Proton chemical shifts (δ) were measured in parts per million (ppm) and referenced to the appropriate solvent peaks. Shifts are reported to the nearest 0.01 ppm, and coupling constants (*J*) are reported to the nearest 0.1 Hz. Multiplicities are reported using singlet (s), doublet (d), triplet (t), quartet (q), multiplet (m), and combinations thereof.

#### *Synthesis of* N,N*′-dicyclohexyl PDI (CH-PDI)*

Perylene-3,4,9,10-tetracarboxylic dianhydride (390 mg, 1 mmol, 1 equiv), Zn(OAc)_2_·2H_2_O (196 mg, 4 mmol, 4 equiv), cyclohexylamine (343 μl, 3 mmol, 3 equiv), and imidazole (4 g) were mixed under argon at 160°C for 2 hours. The reaction was cooled, and the crude product was dispersed in EtOH (150 ml), 2 M HCl (100 ml), and water (100 ml) and left stirring overnight in air at room temperature. The precipitate was collected by filtration, washed with water, and dried. The solid was purified by column chromatography (silica gel; CHCl_3_/EtOAc, 9:1) to yield CH-PDI as a dark red solid (283 mg, 0.51 mmol, 51%). ^1^H NMR (500 MHz, CDCl_3_) δ 8.67 (d, *J* = 7.7 Hz, 4H), 8.62 (d, *J* = 7.7 Hz, 4H), 5.05 (tt, *J* = 3.6 Hz, *J* = 3.6 Hz, 2H), 2.58 (qd, *J* = 12.3 Hz, *J* = 3.5 Hz, 4H), 1.94 to 1.90 (m, 4H), 1.80 to 1.73 (m, 6H), 1.48 to 1.32 (m, 6H). The NMR data were in accordance with those reported previously (*89*).

#### *Synthesis of* N,N*′-bis(4-heptyl) PDI (ST-PDI)*

Perylene-3,4,9,10-tetracarboxylic dianhydride (810 mg, 2 mmol), 4-heptylamine (717 μl, 4.8 mmol), and imidazole (5.5 g) were mixed under argon at 160°C for 3 hours. The reaction was cooled, and the crude product was dissolved in chloroform, separated with water and 2 M HCl, dried over magnesium sulfate, filtered, and reduced in vacuo. The solution was purified by column chromatography (silica gel; CHCl_3_/MeOH, 100:1). The first red fraction was collected and dried in vacuo, yielding ST-PDI as a red solid (960 mg, 91%). ^1^H NMR (400 MHz, CDCl_3_) δ 8.68 to 8.63 (m, 8H), 5.24 to 5.28 (m, 2H), 2.61 to 2.22 (m, 4H), 1.86 to 1.77 (m, 4H), 1.37 to 1.28 (m, 8H), 0.92 (t, *J* = 7.3 Hz, 12H). The NMR data were in accordance with those reported previously (*90*).

### Nanobelt fabrication

Nanobelts were produced by self-assembly of PDI molecules using a phase transfer method, as outlined previously (*89*). A concentrated solution of either CH-PDI or ST-PDI (0.5 ml, 0.3 mM) was prepared in CHCl_3_ in a glass vial. This solution was then injected to the bottom of a vial containing ethanol (~11:3 volume ratio, EtOH:CHCl_3_). The solution was allowed to sit in the dark at room temperature for 24 hours, and nanobelts were formed at the interface of the solvents. The solution was not shaken during the self-assembly process to prevent the formation of nanobelts with sharp ends. After mixing the two solvents, the nanobelts diffused into the whole solution phase and were transferred onto glass coverslips by pipetting. The samples were then allowed to dry in a N_2_ glove box.

### Femtosecond transient absorption microscopy (fs-TAM)

The fs-TAM setup used in this work was described in detail previously (*19*–*21*). Briefly, a broadband sub–10 fs pump pulse (520 to 650 nm) is focused to the diffraction limit (FWHM ~270 nm) by a high–numerical aperture (~1.1) objective onto the sample. A wide-field, counterpropagating probe pulse (8 fs, 680 to 790 nm, FWHM ~15 μm) focused with a concave mirror is used to monitor the normalized change in image transmission with pump on (*T*_on_) and off (*T*_off_) as a function of the time delay between the pulses [Δ*T*/*T* = (*T*_on_ − *T*_off_)/*T*_on_]. By subtracting the extent of the spatial profile at a time *t* from the profile when there is no time delay between the pump and the probe (*t*_0_), the propagation of the carrier population can be monitored. The only limit to localization precision is how well the different spatial profiles can be resolved. On the basis of the signal-to-noise ratio of the measurement, the localization precision is typically ~10 nm, enabling the observation of spatiotemporal dynamics well below the diffraction limit.

Pulses were delivered by a Yb:KGW amplifier (Pharos, LightConversion) that seeded two broadband white light (WL) stages at a repetition rate of 200 kHz. The probe WL was generated in a 3-mm yttrium-aluminum-garnet crystal and adjusted to cover the wavelength range from 650 to 950 nm by a fused-silica prism–based spectral filter. In contrast, the pump WL was generated in a 3-mm sapphire crystal to extend the WL at high frequency to 500 nm and short-pass filtered at 650 nm (Thorlabs). A set of chirped mirrors (pump, Layertec; probe, Venteon) and a pair of fused silica wedges (Layertec) compressed the pulses to sub–10 fs, as verified by second-harmonic generation frequency–resolved optical gating. The pump pulse was focused onto the sample using a 1.1–numerical aperture oil immersion objective (100×, Nikon). Using the full bandwidth of the pump pulse centered at 600 nm, we achieved a diffraction-limited spatial resolution of ~270 nm (FWHM). The probe pulse was projected onto the sample in the wide field by a concave mirror (FWHM ~15 μm).

Transmitted light was collected by the same objective used to focus the pump and sent to an imaging system based on an electron multiplying charge-coupled device (CCD) camera (QuantEM:512SC, Photonmetrics). Pump light was suppressed by a 650-nm long-pass filter (Thorlabs) inserted in front of the camera. A bandpass filter (Semrock/Thorlabs) was additionally placed in front of the camera to image the desired probe wavelength. Differential imaging was achieved by modulating the pump beam at 45 Hz by a mechanical chopper. An additional autofocus line based on the total internal reflection of a 405-nm continuous wave laser beam was used to maintain axial focus on the sample throughout measurements.

Microabsorption spectra were calculated from combined microreflection and microtransmission measurements. Specifically, the reflection spectrum R(λ) of an individual nanobelt was measured with respect to a silver (Ag) mirror as the reference, while the transmission spectrum T(λ) was obtained by normalizing the transmitted signal to that of the bare substrate. The absorption A(λ) was then calculated using the relation

A(λ)=1−R(λ)−T(λ) (1)

This approach allows extraction of the intrinsic absorption characteristics of the nanobelt while minimizing substrate- and system-related effects.

### PXRD data collection

PXRD data were recorded at 294 K on a Bruker D8 Diffractometer operating in transmission mode (Ge-monochromated Cu Kα_1_ radiation; λ = 1.54056 Å; Våntec detector covering 3° in 2θ). For CH-PDI, two PXRD datasets were used in this study, referred to as dataset 1 (2θ range, 4° to 70°; 2θ step size, 0.016°; time per data point, 13 s; data collection time, 15.53 hours) and dataset 2 (2θ range, 3.6° to 50°; 2θ step size, 0.016°; time per data point, 70 s; data collection time, 59.95 hours). For the sample comprising a mixture of polymorphs I and II of ST-PDI, PXRD data were recorded as follows: 2θ range, 3.5° to 30°; 2θ step size, 0.016°; time per data point, 40 s; data collection time, 20.4 hours.

### PXRD data analysis: Profile fitting and Rietveld refinement

Profile fitting and unit cell refinement from the PXRD data recorded for CH-PDI and from the PXRD data recorded for the sample comprising a mixture of polymorphs I and II of ST-PDI were carried out using the Le Bail method (*37*) in the GSAS program (*91*). For CH-PDI, structure refinement was carried out from PXRD data using the Rietveld profile refinement technique in the GSAS program (*91*), involving refinement of atomic positions, isotropic atomic displacement parameters (*U*_iso_), unit cell parameters, zero-point shift parameter, and profile parameters. In general, a common value of *U*_iso_ was refined for all nonhydrogen atoms (or for specific groups of nonhydrogen atoms). The value of *U*_iso_ for hydrogen atoms was set as 1.2 times the refined value of *U*_iso_ for the nonhydrogen atoms to which they are bonded. To ensure that the geometries of the CH-PDI molecules remained reasonable in the Rietveld refinement, restraints were applied to bond lengths and bond angles. The restraints were based on the molecular geometries in the crystal structure generated by applying DFT-D geometry optimization to the crystal structure obtained in the structure solution calculations for CH-PDI (see the “Structure solution and refinement from 3D-ED data” section). Planar restraints were also applied to maintain the planarity of the aromatic ring system. The restraints on molecular geometry were progressively relaxed toward the end of the Rietveld refinement. Preferred orientation correction was carried out using the March-Dollase method (*48*, *49*).

### 3D-ED data collection

3D-ED data were recorded using the continuous rotation electron diffraction method on a JEOL JEM-2100 TEM operated at an accelerating voltage of 200 kV. The PDI nanobelts were deposited on standard holey carbon–coated 200-mesh Cu TEM grids (EM Resolutions Ltd.) mounted in a Gatan cryo-transfer tomography holder (no. 914). The sample was cooled to a temperature of ~100 K. During data collection, the goniometer was rotated continuously with speed values of 0.276°/s for the CH-PDI sample and 0.23°/s for the ST-PDI sample. During tilting, the crystal was tracked by sequential defocusing of the intermediate lens using the software Instamatic (*92*). The diffraction patterns were collected using the high-speed hybrid detection camera Timepix Quad (ASI). The datasets were processed using XDS (X-ray Detector Software) (*93*) to extract intensities for structure solution and refinement. The CH-PDI and ST-PDI nanobelts were typically single crystals, and selected electron diffraction patterns acquired near crystallographic zone axes during this tilt series are shown in fig. S37.

### Structure solution and refinement from 3D-ED data

Structure solution of CH-PDI and polymorphs I and II of ST-PDI was carried out from 3D-ED data using the direct-space GA strategy implemented in the program EAGER. This program was originally developed for structure solution from PXRD data (*38*–*44*, *94*) and has recently been adapted for 3D-ED data (*23*, *25*, *45*, *46*) [using electron scattering factors from Lobato and Van Dyck (*47*)]. Before the structure solution calculations, the 3D-ED intensity data were processed to remove reflections with negative intensities and reflections corresponding to systematic absences in the assigned space group, and the intensities of symmetry-equivalent reflections were averaged. For CH-PDI, the 3D-ED data had a maximum 2θ of 1.52276° (λ = 0.0251 Å), corresponding to a resolution of 0.94 Å. For polymorphs I and II of ST-PDI, the 3D-ED data had at a maximum 2θ of 1.4° (λ = 0.0251 Å), corresponding to a resolution of 1.03 Å. In each case, the unit cell used in the structure solution calculations was taken from the results of profile fitting and unit cell refinement from PXRD data (rather than the unit cell determined from the 3D-ED data). Specific details of the direct-space GA structure-solution calculations (including the method to define the trial structural models) are given for CH-PDI in section S4.2 and for ST-PDI in section S5.2.

In the direct-space GA structure-solution calculations, the quality of each trial structure was assessed from the agreement between the 3D-ED data calculated for the trial structure and the experimental 3D-ED data using the figure-of-merit *R*_F_

$$R_{F}=\frac{\sum_{\mathrm{hkl}}||F_{\text{calc}}|-|F_{\text{exp}}||}{\sum_{\mathrm{hkl}}|F_{\text{exp}}|}$$ (2)

where |*F*_calc_| and |*F*_exp_| are the calculated and experimental structure factor amplitudes, respectively. The experimental structure factor amplitudes were determined from the experimentally measured intensities (*I*_exp_) assuming |*F*_exp_| ∝ (*I*_exp_)^1/2^.

Structure refinement of polymorphs I and II of ST-PDI was carried out from 3D-ED data within the kinematical approximation using atomic scattering factors for electrons in the program SHELXL-97 (*95*). Details of the 3D-ED data collections and results from the structure refinement of polymorphs I and II of ST-PDI are given in section S5.4 (see also tables S3 and S4). Structure refinement of CH-PDI was carried out by Rietveld refinement from PXRD data, as discussed in the “PXRD data analysis: Profile fitting and Rietveld refinement” section.

Crystallographic information files (CIFs) of the structures of CH-PDI and polymorphs I and II of ST-PDI determined in this work have been deposited in the Cambridge Structural Database. We note that for these CIFs, various alerts arise in checkCIF reports as a consequence of the fact that the structure refinements were carried out from PXRD data (for CH-PDI) and 3D-ED data (for polymorphs I and II of ST-PDI). In this regard, it is important to highlight that the thresholds for alerts in checkCIF are based on the expectation and assumption that high-quality single-crystal XRD data were used for structure determination, and these thresholds are generally not realistic in the case of PXRD data and 3D-ED data. The CIFs that we have deposited in the Cambridge Structural Database contain comments that we have added to explain the reasons that A-level alerts arise in checkCIF reports in the case of structure determination from PXRD data and 3D-ED data. We emphasize that the quality of the PXRD data and 3D-ED data used in the current work was sufficiently high to allow successful structure determination of CH-PDI and polymorphs I and II of ST-PDI, leading in each case to a structure that is clearly correct based on structural and chemical considerations, and fully validated from our DFT-D studies, as discussed in the “Structure determination” section and sections S4.3 and S5.5. To facilitate inspection of the final refined crystal structures, we also include as the Supplementary Materials (section S11 and files S1 to S3) the CIFs for the crystal structures generated after subjecting the final refined crystal structures to periodic DFT-D geometry optimization.

### Solid-state ^13^C NMR spectroscopy

For the sample comprising a mixture of polymorphs I and II of ST-PDI, high-resolution solid-state ^13^C NMR spectra were recorded on a Bruker AVANCE III spectrometer at the UK High-Field (850 MHz) Solid-State NMR Facility (magnetic field, 20.0 T; ^13^C Larmor frequency, 213.81 MHz; ^1^H Larmor frequency, 850.23 MHz). The spectra were recorded using ramped ^1^H → ^13^C cross polarization, together with magic-angle sample spinning at 12 kHz and high-power ^1^H decoupling. High-resolution solid-state ^13^C NMR spectra were also recorded using ^13^C rotor-synchronized dipolar dephasing, with dephasing times of 100 and 200 μs.

### DFT-D calculations

#### Geometry optimization of crystal structures

At various stages of the structure determination procedures, periodic DFT-D geometry optimization calculations were carried out using CASTEP (academic release version 21.1.1) (*56*). These calculations used ultrasoft pseudopotentials (*96*), the PBE functional (*97*), semiempirical dispersion correction using the TS correction scheme (*98*), preserved space group symmetry, periodic boundary conditions, a basis set cutoff energy of 700 eV, and a Monkhorst-Pack grid (*99*) of minimum sample spacing (0.05 × 2π) Å^−1^. The convergence criteria for geometry optimization were 0.01 eV Å^−1^ for the maximum atomic force, 0.00001 eV per atom on the total energy, and 0.001 Å for atomic displacements. The DFT-D geometry optimization calculations were carried out with preservation of space group symmetry and either with fixed unit cell or with relaxation of the unit cell.

#### DFT-GIPAW calculations of solid-state ^13^C NMR data

Calculations of solid-state ^13^C NMR data for the crystal structures of polymorphs I and II of ST-PDI were carried out using the DFT-GIPAW methodology in the program CASTEP (academic release version 21.1.1) (*56*) to allow comparison with experimental solid-state ^13^C NMR data. Periodic DFT-D calculations to determine the isotropic ^13^C NMR chemical shifts for the crystal structure of each polymorph were based on the DFT methodology described in the “Geometry optimization of crystal structures” section. The isotropic ^13^C NMR chemical shielding values were calculated using the GIPAW approach (*56*–*59*), with a cutoff energy of 700 eV. From the isotropic ^13^C NMR shielding value (σ_calc_) calculated for each ^13^C environment in the crystal structure, the corresponding calculated isotropic ^13^C NMR chemical shift (δ_calc_) is determined from the equation (*100*)

$$\delta_{\text{calc}}=\delta_{\text{ref}}-\sigma_{\text{calc}}$$ (3)

where δ_ref_ denotes the chemical shift reference. In the present work, the calculated ^13^C chemical shifts were referenced separately for the region of the spectrum with δ > 46 ppm, corresponding to the ^13^C environments in the aromatic ring system and the N─^13^CH environment in the 4-heptyl substituents, and for the region of the spectrum with δ < 46 ppm, corresponding to the ^13^CH_2_ and ^13^CH_3_ environments in the 4-heptyl substituents. Specifically, δ_ref_ = 170 ppm for the region δ > 46 ppm, and δ_ref_ = 173 ppm for the region δ < 46 ppm. These values of δ_ref_ were selected to give the best match (based on visual assessment) between the experimental and calculated solid-state ^13^C NMR spectra in each region.

### Scanning electron diffraction

SED data were acquired using a JEOL GrandARM 300F (S)TEM operated at an accelerating voltage of 300 kV (λ_e_ = 1.97 pm) with a probe current of 1.8 pA and a convergence semiangle α of ~0.8 mrad. Assuming a diffraction-limited probe diameter (the diameter marked by the first zeros in an Airy function, *d*_diff_ = 1.22 λ_e_/α) of ~3 nm and taking the illuminated area as a disk corresponding to the diffraction-limited diameter, the fluence was estimated at 15.9 e^−^ Å^−2^. Electron diffraction patterns were recorded using a Merlin/Medipix detector (Quantum Detectors) with 256 by 256 pixels at a camera length of 20 cm, giving a calibrated pixel size of 0.010 Å^−1^/px. Scans were performed with a calibrated step size of 7.73 nm/px over 256 by 256 probe positions. VDF images were formed by plotting the summed intensity in the diffraction plane in the scattering vector magnitude range of 0.75 to 1 Å^−1^ as a function of probe position using the pyXem Python library (*101*).

### Electron energy loss spectroscopy (EELS)

EELS and BF-STEM were performed using a Hermes UltraSTEM 100MC (Nion) electron microscope. The microscope was operated at 60 kV, while the electron optics were adjusted to a convergence angle of ∼32 mrad and an electron probe <1 Å. For measurements of the optical states (1 to 10 eV), the energy resolution was set to ~25 meV [FWHM of the zero loss peak (ZLP)]. For measurements of vibrational states (<0.5 eV), the energy resolution was set to ~15 meV. The energy resolution was controlled by adjusting the width of the energy-selecting slit in the monochromator. The microscope was equipped with a postcolumn Enfinium ERS dedicated spectrometer (Gatan) and coupling module to control collection for EELS, which was set to a collection of a semiangle of 44 mrad. EELS data were acquired in “dualEELS” mode with near-simultaneous acquisition of the ZLP and a higher-energy window with the ZLP shifted off the CCD camera, which was particularly necessary for exposure times sufficient to record EEL spectra at infrared and visible energies with a suitable signal-to-noise ratio. In all cases, bright-field micrographs were acquired after all EEL spectrum images for the nanobelt. For the nanobelt shown in Fig. 5 and fig. S31, the bright-field micrograph was acquired after both EELS datasets. This sequence of experiments was necessary to minimize deleterious beam damage. Separate measurements indicated that EELS acquired under comparable dose conditions could be obtained from the edge of PDI nanobelts (as evidenced by no loss in signal for line profiles along the nanobelt) but that scanning across the nanobelts resulted in substantial loss of intensity at visible and ultraviolet energies. Diffraction contrast features in BF-STEM were likewise lost after the first BF-STEM acquisition. The BF-STEM diffraction contrast, however, was not substantially modified by the EELS data acquired first, indicating that any damage accompanying the EELS acquisition was not extensively delocalized.

Additional EELS data for nanobelt thickness estimation were acquired on a Thermo Fisher Scientific Osiris microscope equipped with a high-brightness “X-FEG” electron source and a Gatan Enfinium spectrometer. The microscope was operated at 80 kV, and the beam convergence semiangle was set to 11.0 mrad. The thickness was determined from analysis of the low-loss EELS, where, under assumptions of Poissonian scattering, the relative thickness *t* can be estimated according to an expression of the form *t*/λ = ln(*I*_tot_/*I*_0_), where λ is the inelastic mean free path, *I*_tot_ is the estimated total scattering (well estimated in low-loss EELS as the bulk plasmon dominates the total inelastic contribution), and *I*_0_ is the elastic contribution (ZLP) (*102*). To determine the thickness in absolute terms, a value of λ is required, calculated here according to the methods reported by Iakoubovskii *et al.* (*103*).

EELS data were processed in HyperSpy (*104*), an open-source software coded in Python. The spectra were first aligned using the ZLP. Initially, the spectral shifts were determined approximately by the maximum pixel intensity followed by a subpixel cross-correlation–based routine. X-ray spikes on the CCD detector were removed by a routine that automatically identified outlying high-intensity pixels and then performed interpolation in the spectral region after the removal of the x-ray spike. An additional energy calibration correction was applied by acquiring dualEELS data with the ZLP recorded near-simultaneously to account for a systematic offset between the “low”- and “high”-energy windows. Uncertainties associated with this correction as well as additional sources of error in the calibration of the spectrometer dispersion produce uncertainties in the absolute energies in the EEL spectra, and absolute energies are more precisely captured in the optical absorption spectra.

ICAs were carried out as reported previously (*105*–*107*). First, principal components analysis decomposition was performed, and a scree plot was used to identify the approximate number of components required to account for the variance in the data, generally ≤6 for these datasets. Components included features attributed to the ZLP tail, detector offset, and thickness-dependent signal variation in spectrum images as well as bulk and spatially localized features. ICA maps were not directly interpreted as physically meaningful spectra and maps. Instead, maps showing isolated features were used as a guide to extract minimally processed selected area spectra. To avoid artifacts arising from background subtraction, difference spectra were calculated following methods for examining subtle spectral changes in vibrational EELS (*108*). The selected area windows were selected to include sample regions of the same thickness and same relative area of sample and vacuum in the selected window at the edge of the nanobelts. To correct for fluctuations in the incident beam current, spectra were further normalized to the ZLP tail intensity. The relative intensity recorded at the π-plasmon region (5 to 8 eV), although not perfectly matched in all nanobelts, showed further consistency in the spectra used to calculate the difference spectra. The oscillations above and below zero in the difference spectra highlighted spectral windows where the EELS signal was both stronger and weaker at the area associated with the defect relative to the area associated with the bulk. In all cases, the defect spectrum was subtracted from the bulk spectrum to give the difference spectrum. These spectra were then lastly correlated with BF-STEM micrographs after processing the selected area spectra.

### Computation of electronic excitation spectra and exciton diffusion

Electronic structures of single CH-PDI and ST-PDI molecules were optimized using DFT as implemented in Turbomole 7.8 (*109*). Then, the transition frequencies and corresponding transition dipoles were computed for the 10 lowest singlet electronic excitations using TD-DFT. All computations were done with the triple-ζ def2-TZVP basis set (*110*) and the B3-LYP hybrid functional (*111*). The choice of the basis set and the functional was guided by the balance between the accuracy and the computational cost. The tight-binding excitonic Hamiltonian of molecular aggregates was constructed using the model described in detail in (*79*). Specifically, molecular packing was taken from the DFT-D–optimized crystal structures, and the coupling between the lowest molecular excitations was described using the Förster interaction term (*112*). The excitonic spectra of aggregates were computed by diagonalizing the constructed Hamiltonians. Two types of disorder were included in the model Hamiltonian. First, a random variation of intramolecular transition frequencies, a static frequency disorder, was introduced to account for fluctuations in the local environment of the molecules (*113*). Second, to include disorder in molecular packing, a random rotation drawn from a uniform distribution of rotations from 0° up to a maximum value of Δθ_max_ (with values of Δθ_max_ in the range of 0° to 90°) was applied to the molecules around the vector normal to the molecular plane. This rotational disorder preserves the π−π stacking of the molecules.

Exciton dynamics in stacks of PDI molecules were simulated using the quantum stochastic equation with a random phase fluctuation, that represents dynamic disorder in the system (*79*). The exciton wave function was propagated for 100 fs and averaged over 100 trajectories corresponding to different realizations of static and dynamic disorder. Then, the diffusion coefficient was computed from a linear fitting of the last 20 fs of the wave function second moment.

## Supplementary Materials

### The PDF file includes:

Supplementary Text Figs. S1 to S37 Tables S1 to S9 Legends for files S1 to S3 References
