# Supplementary material for: Structural properties, polymorphism, and multiscale disorder unravel energy transport limitations in perylene diimide semiconductors
Source: Sci Adv. 2026 May 27;12(22):eaed0037. doi: 10.1126/sciadv.aed0037 (PMC13215177; doi:10.1126/sciadv.aed0037)
Supplement: Supplementary file 1 — Supplementary Text Figs. S1 to S37 Tables S1 to S9 Legends for files S1 to S3 References [file sciadv.aed0037_sm.pdf]

Science Advances
AAAS

Supplementary Materials for

Structural properties, polymorphism, and multiscale disorder unravel energy transport limitations in perylene diimide semiconductors

Christopher J. H. Smalley *et al.*

Corresponding author: Kenneth D. M. Harris, harriskdm@cardiff.ac.uk;
Sean M. Collins, s.m.collins@imperial.ac.uk

Sci. Adv. **12**, eaed0037 (2026)
DOI: 10.1126/sciadv.aed0037

The PDF file includes:

Supplementary Text
Figs. S1 to S37
Tables S1 to S9
Legends for files S1 to S3
References

Other Supplementary Material for this manuscript includes the following:

Files S1 to S3

S1. Sample Overview

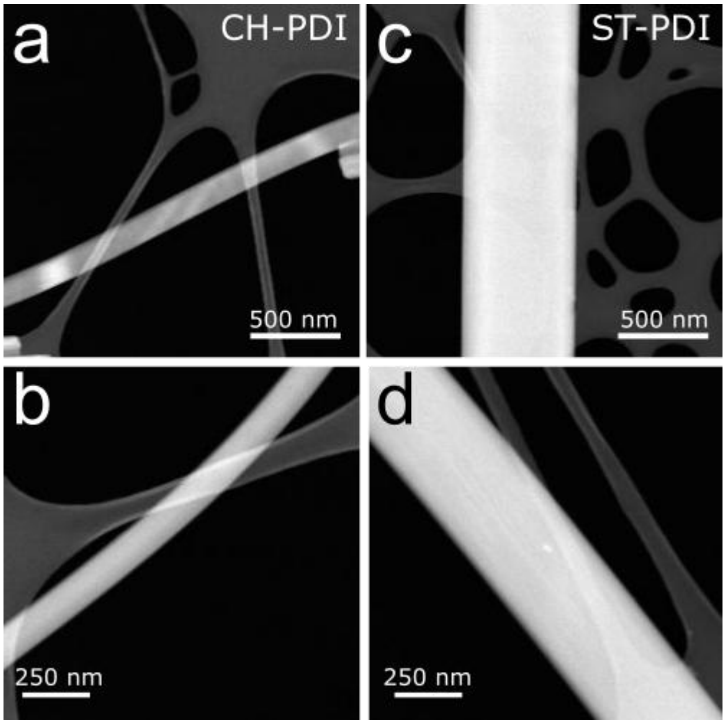

Figure S1. Additional examples of CH-PDI and ST-PDI nanobelts. (a,b) CH-PDI and (c,d) ST-PDI nanobelts recorded by scanning electron diffraction (SED).

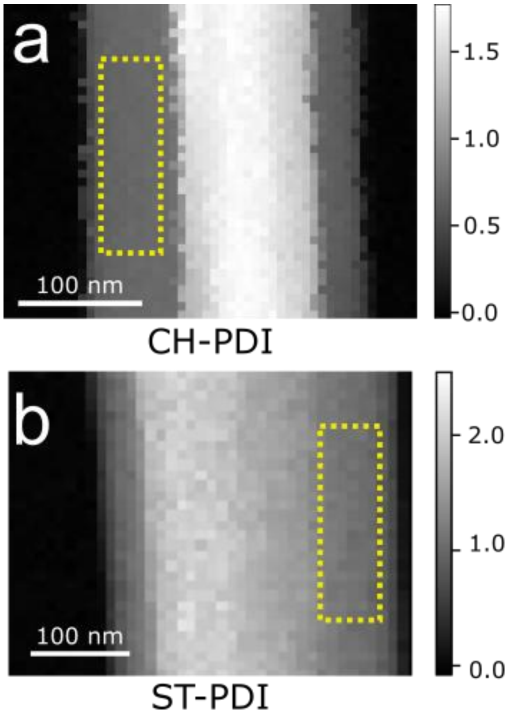

Figure S2. Thickness mapping of CH-PDI and ST-PDI nanobelts. Thickness maps determined by EELS for additional areas showing overlapping (a) CH-PDI and (b) ST-PDI nanobelts. The intensities are in thickness units of the inelastic mean free path (t/λ). The yellow dashed rectangles indicate regions identified as single (non-overlapped) nanobelts used to determine the average thickness in **Table S1**.

Table S1. Absolute thickness of CH-PDI and ST-PDI nanobelts. Absolute thickness estimation from EELS following the method proposed by Iakoubovksii et al. (*103*). Densities (ρ) were taken from experimentally determined crystal structures. The convergence semiangle (α) and collection semiangle (β) are given in each case alongside the mean *t/λ* extracted from the areas indicated in **Figure S2**.

Sample	Instrument	Beam energy (keV)	ρ (g/cm^3)	α (mrad)	β (mrad)	*t/λ* $\bar{x} \pm 1\sigma$	t (nm)
CH-PDI (Fig. S2a)	Nion UltraSTEM100MC	60	1.34	31	44	0.70 ± 0.02	59
ST-PDI (Fig. S2b)	FEI Osiris	80	1.26	11	24.5	1.1± 0.1	120

S2. Extracting Population Propagation from Pump-Probe Spectroscopy

The spatial pump–probe signal in transient absorption microscopy will be related to changes in the real (Δn) and imaginary (Δk) parts of the refractive index, respectively. As highlighted by Ashoka *et al.* (*36*) extracting population propagation by 2D Gaussian fitting of the pump–probe microscopy signals, as we do here, is only appropriate in the limit of Δn and Δk changing to similar extents. Taking the pump probe spectra of CH-PDI and ST-PDI (see Ref. (*19*) and Ref. (*114*), respectively) and applying the model from Ashoka et al. (*115*) to extract of Δn(t) and Δk(t) following a photoexcitation, we find that for both systems Δn(t)/Δk(t) is constant when probing at 780 nm (within 5 ps of photoexcitation), whereas at 650 nm (at the edge of the absorption of both molecules) it varies significantly (see **Figure S3**). As such we extract the mean-square displacement and transport information when probing at 780 nm.

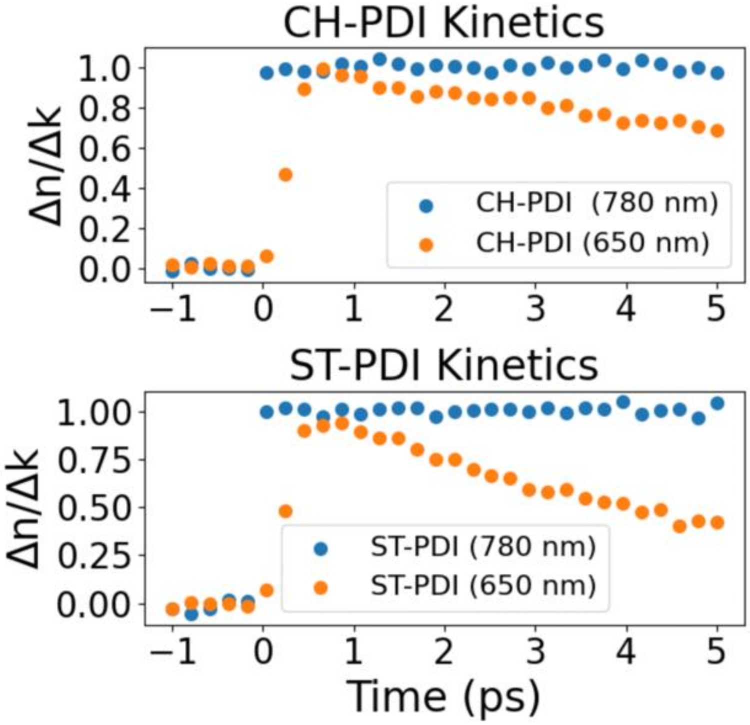

Figure S3. Evaluation of the suitability of pump-probe microscopy signals for extracting population propagation. $\Delta n(\mathrm{t})/\Delta k(\mathrm{t})$ extracted from transient absorption spectroscopy measurements of (upper panel) CH-PDI and (lower panel) ST-PDI at 780 nm and 650 nm.

S3. Distribution of Diffusion Coefficients in ST-PDI Nanobelts

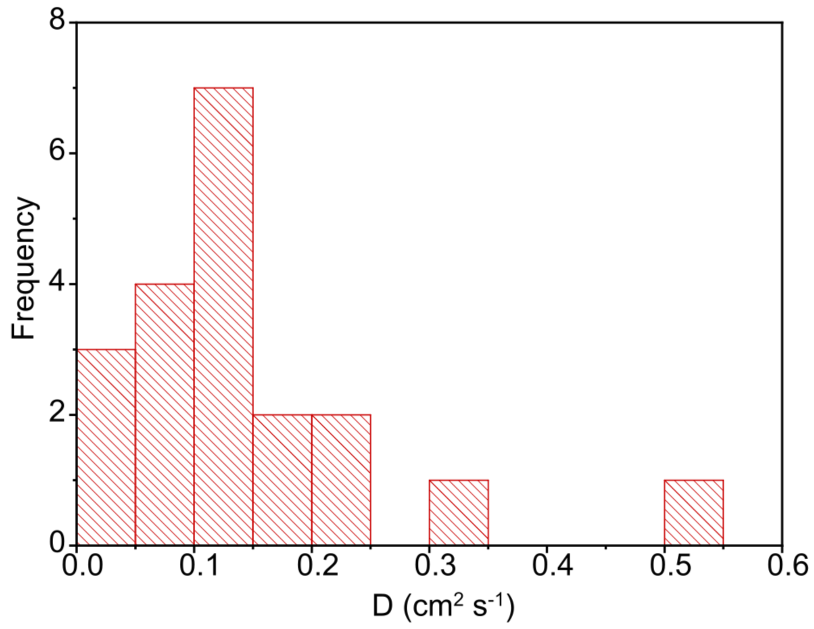

Figure S4. Spread of diffusion coefficients determined from ST-PDI nanobelts. Histogram of diffusion coefficients determined from 20 locations on dispersed nanobelts of ST-PDI by femtosecond transient absorption microscopy.

S4. Structure Determination of CH-PDI

S4.1. Unit Cell Determination from PXRD Data

Profile-fitting and unit cell refinement was carried out for PXRD dataset 1 of CH-PDI using the Le Bail method. The initial unit cell parameters and space group were based on those determined from 3D-ED data ($Pna2_1$; a = 22.88 Å, b = 7.24 Å, c = 37.61 Å). A good-quality fit to the PXRD data was obtained (**Figure S5**; R_p = 0.93%, R_{wp} = 1.22%), with the following refined unit cell parameters: a = 21.1664(26) Å, b = 7.07647(23) Å, c = 36.542(4) Å. This result confirmed that the powder sample of CH-PDI contains only one crystalline phase and confirmed that the structure is orthorhombic with space group $Pna2_1$.

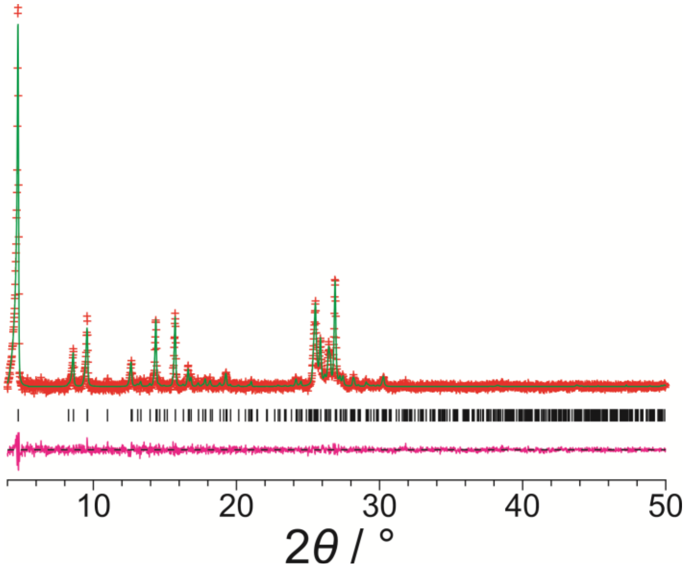

Figure S5. Profile fitting PXRD data for CH-PDI. Le Bail fitting of PXRD dataset 1 (baseline subtracted) for CH-PDI (red + marks, experimental data; green line, calculated data; magenta line, difference plot; black tick marks, peak positions).

S4.2. Direct-Space Structure Solution from 3D-ED Data

As the PXRD data for CH-PDI are influenced by significant preferred orientation (see **Section S4.3**), it was considered to be more reliable to carry out structure solution of CH-PDI using the 3D-ED data. Structure solution was carried out using the direct-space genetic-algorithm (GA) technique implemented in the program EAGER (see **Materials and Methods Section 4.7**). The unit cell determined from Le Bail fitting of the PXRD data was used, as this unit cell is considered to be more accurate than the unit cell determined directly from the 3D-ED data. Given the unit cell volume, density considerations indicate that there are eight molecules of CH-PDI in the unit cell, and hence two molecules of CH-PDI in the asymmetric unit for space group $Pna2_1$. The molecular geometry used in the direct-space structure-solution calculations was taken from DFT geometry optimization of an isolated molecule, with the two cyclohexyl substituents in the chair conformation. In the direct-space GA calculation, one of the two independent molecules in the asymmetric unit was defined by seven structural variables: two positional variables (for space group $Pna2_1$, the position of one molecule along the *z*-axis can be fixed arbitrarily), three orientational variables and two torsional variables (corresponding to rotation around each of the two N–C bonds that link the cyclohexyl substituents to the aromatic ring system). The other molecule in the asymmetric unit was defined by eight structural variables: three positional variables, three orientational variables and two torsional variables (as defined above). Thus, the total number of structural variables in the direct-space GA calculation was 15. In total, 40 independent GA structure solution calculations were carried out, with a population of 100 trial structures in each case. Each independent GA calculation started from a different randomly generated initial population, which was allowed to evolve for 500 generations with 10 mating events and 50 mutation events per generation. The quality of agreement between the 3D-ED data calculated for each trial structure and the experimental 3D-ED data was assessed using the figure of merit R_F, which was used in the definition of the fitness of each trial structure in the GA

calculation. The evolutionary progress in each of the 40 independent GA structure-solution calculations is shown as a function of generation number in **Figure S6**.

The structure solution with the lowest R_F in the final population in each of the 40 independent GA calculations was then examined. Among these 40 structure solutions, 38 structures were essentially identical in terms of the stacking of CH-PDI molecules along the *b*-axis, with the two independent molecules alternating along the stack. However, these 38 structure solutions differed in terms of the orientations of the cyclohexyl rings relative to the aromatic ring system in the two independent molecules. In this regard, we define the conformation of the CH-PDI molecule in terms of whether the two cyclohexyl groups are in a *syn* orientation or an *anti* orientation with respect to each other (defined in **Figure S7**). We note that, in the best structure solutions, the mean plane of each cyclohexyl group is approximately perpendicular to the aromatic ring system, which corresponds to the C–H bonds highlighted in **Figure S7** (i.e., for each cyclohexyl group, the C–H bond for the C atom that is bonded to the N atom the aromatic ring system) lying close to the plane of the aromatic ring system. In 37 of the structure solutions (including the 35 structure solutions with lowest R_F), the asymmetric unit contained one molecule in the *syn* conformation and one molecule in the *anti* conformation. However, in one structure solution (ranked 37th lowest in R_F), the two independent molecules in the asymmetric unit were both in the *anti* conformation. The structure solution with lowest R_F (containing one *syn* molecule and one *anti* molecule in the asymmetric unit) was used as the starting structural model for Rietveld refinement from the PXRD data.

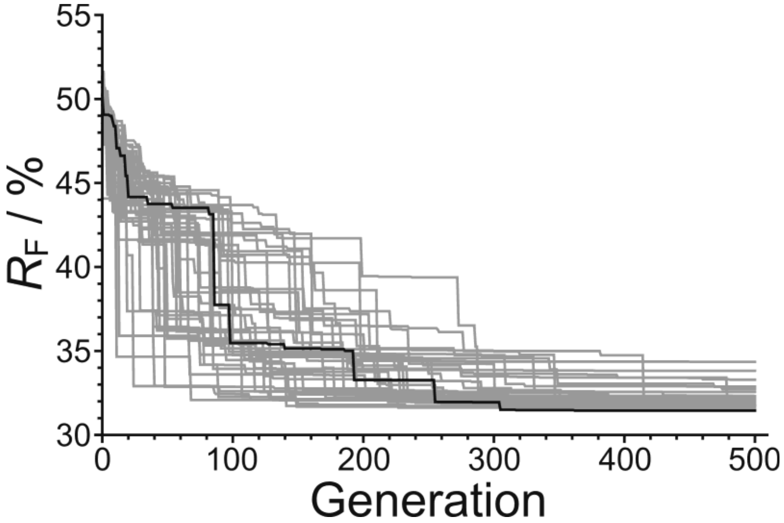

Figure S6. Evolutionary progress in the GA structure solution calculations from the 3D-ED data for CH-PDI. The evolution of each of the 40 independent GA calculations (each starting from a different random initial population of trial structures) is represented by a continuous grey line showing the lowest value of R_F among all 100 trial structures in the population as a function of generation number. The continuous line shown in black corresponds to the GA calculation that generates the structure with lowest R_F after 500 generations.

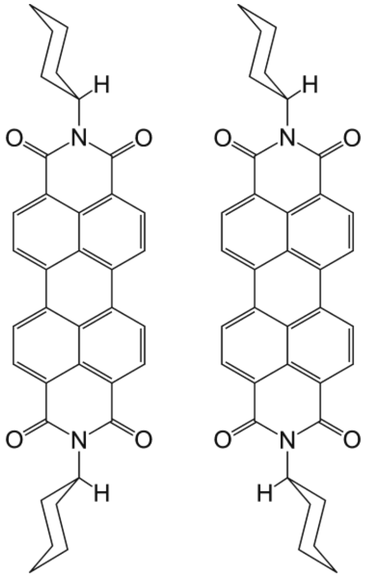

Figure S7. Definition of the *syn* and *anti* conformations of the CH-PDI molecule. The conformations depend on the relative orientations of the two cyclohexyl substituents. In all structures, the mean planes of the two cyclohexyl substituents in the CH-PDI molecule are approximately perpendicular to the aromatic ring system, with the C–H bond highlighted for each cyclohexyl substituent lying close to the plane of the aromatic ring system (i.e., with the C–N–C–H torsion angle close to 0° or 180°).

S4.3. Structure Refinement from PXRD Data

Rietveld refinement was initially carried out using PXRD dataset 1 of CH-PDI following the general method described in **Materials and Methods Section 4.5** (except where stated otherwise below), with a preferred orientation correction required in order to achieve an acceptable quality of fit between the experimental and calculated PXRD data. The refinement resulted in a good quality of fit (R_p = 1.37%; R_{wp} = 1.87%). The preferred orientation correction used the March-Dollase method with (101) as the preferred orientation plane and with a refined March factor of 1.82.

In the refined structure, the orientations of the cyclohexyl rings in the two crystallographically independent molecules were the same as those in the starting structural model (taken from the structure solution discussed in **Section S4.2**), corresponding to one *syn* molecule and one *anti* molecule in the asymmetric unit. The structure from this initial Rietveld refinement was subjected to DFT-D geometry optimization with fixed unit cell to determine the energetically favored conformation of the two independent CH-PDI molecules in the crystal structure. For these calculations, four variants of the structure were generated by interconverting one of the two independent molecules between the *syn* and *anti* conformations (specifically by taking the structure from the initial Rietveld refinement and flipping one cyclohexyl ring in one molecule by changing the torsion angle around the relevant N–C bond by 180°). The four variants of the structure generated in this way were then subjected to DFT-D geometry optimization with fixed unit cell. Following DFT-D geometry optimization, one variant of the structure containing both molecules in the *anti* conformation had a lower energy [by 2.28 kJ mol^{-1} (per mole of CH-PDI molecules)] than the DFT-D geometry-optimized structure from the Rietveld refinement. In contrast, the other three variants of the structure following DFT-D geometry optimization had higher energies [by 6.87, 7.91 and 14.51 kJ mol^{-1} (per mole of CH-PDI molecules)] than the DFT-D geometry-optimized structure from the Rietveld refinement.

The structure of lowest energy (in which both molecules in the asymmetric unit have the *anti* conformation) was then used as the starting structure for a final Rietveld refinement using PXRD dataset 2. In this refinement, a common value of U_{iso} was refined for all non-hydrogen atoms of the PDI ring system and a different common value of U_{iso} was refined for all non-hydrogen atoms of the cyclohexyl substituents. Restraints on molecular geometry were applied (as defined in **Materials and Methods Section 4.5**) and were progressively relaxed (although not removed entirely) towards the end of the refinement. This Rietveld refinement gave a good-quality fit to the PXRD data (**Figure S8b**; R_p = 0.81%, R_{wp} = 1.24%), with the following final refined unit cell parameters: a = 21.168(4) Å, b = 7.08303(30) Å, c = 36.661(11) Å. Importantly, the quality of fit obtained in the Rietveld refinement is comparable to the quality of fit obtained in profile-fitting of the same PXRD dataset (dataset 2) using the Le Bail method (**Figure S8a**; R_p = 0.53%, R_{wp} = 0.74%). The final refined value of the March parameter was 1.90, which is indicative of a significant extent of preferred orientation in the powder sample. The final refined crystal structure is shown in **Figure S9** and discussed in **Section S4.4**. Subjecting this structure to DFT-D geometry optimization (with fixed unit cell) resulted in only minor atomic displacements (**Figure S10**), with a root-mean-squared deviation (RMSD) in the positions of non-H atoms of 0.32 Å, confirming that the structure is close to a minimum on the energy landscape.

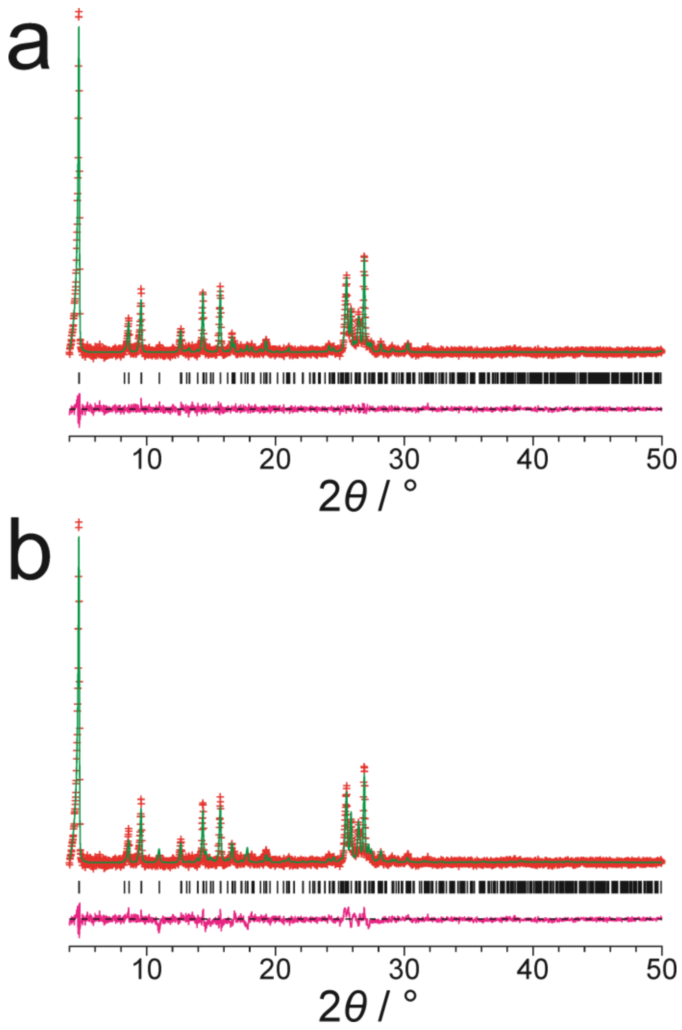

Figure S8. Profile fitting and Rietveld refinement of PXRD for CH-PDI. Results from (a) Le Bail fitting and (b) Rietveld refinement of PXRD dataset 2 (baseline subtracted) for CH-PDI (red + marks, experimental data; green line, calculated data; magenta line, difference plot; black tick marks, peak positions).

S4.4. Discussion of the Crystal Structure of CH-PDI

The crystal structure of CH-PDI (**Figure S9**) comprises stacking of CH-PDI molecules along the *b*-axis, with the two independent molecules in the asymmetric unit representing adjacent molecules in the stack. The conformations of the two independent molecules are very similar, as shown by the overlay of the two molecules in **Figure S11** (the RMSD in atomic positions is 0.18 Å for non-H atoms), with each molecule adopting the *anti* conformation defined in **Figure S7**. The mean plane of each cyclohexyl substituent is close to perpendicular to the plane of the aromatic ring system; thus, for the two cyclohexyl substituents in each molecule, the C–H bond (highlighted in **Figure S7**) of the C atom bonded to the N atom in the aromatic ring system lies close to the plane of the aromatic ring [for one molecule, the C–N–C–H torsion angles for the two cyclohexyl groups are 11.01° and 0.07°; for the other molecule, the C–N–C–H torsion angles are 12.81° and 14.57°].

Each stack of CH-PDI molecules is identical (related by crystal symmetry). The molecules are tilted relative to the stacking axis (**Figure S12**), and the normal to the plane of the aromatic ring system forms angles of 16.8° and 16.9° with respect to the stacking axis for the two independent molecules. As shown in **Figure S13**, the molecules alternate between two orientations in moving along the stack, with the long molecular axis (defined by the intramolecular N···N vector) differing in orientation by *ca*. 38.2° between adjacent molecules. Alternation of the orientations of adjacent molecules in this manner allows the aromatic ring systems to form a favorable π-stacking arrangement, while avoiding unfavorable steric interactions between the cyclohexyl substituents of adjacent molecules. As shown in **Figure S13a**, each stack of CH-PDI molecules has an approximately rectangular cross-sectional shape (when projected on to the plane perpendicular to the stacking axis), and one axis of the rectangular cross-sectional shape is significantly longer than the other axis. In the discussion below, it is convenient to discuss the relative positions and orientations of adjacent stacks in the crystal structure in terms of the packing of the rectangular cross-sectional shapes relative to each other in two dimensions.

The distance between the centers of adjacent molecules along the stack alternates between 3.61 Å and 3.49 Å in the structure determined from Rietveld refinement; however, after subjecting this structure to DFT-D geometry optimization (with fixed unit cell), these distances become 3.54 Å. Thus, the slight alternation in the distance between adjacent molecules along the stack in the structure from Rietveld refinement probably reflects experimental uncertainties (for example, arising from the effects of preferred orientation in the PXRD data and approximations in the methodology to correct for preferred orientation in the Rietveld refinement) rather than a genuine structural feature.

In the structure from Rietveld refinement, the perpendicular distance between the planes of the aromatic ring systems of adjacent molecules in the stacks is *ca*. 3.39 Å; after subjecting the structure to DFT-D geometry optimization with fixed unit cell, this distance becomes *ca*. 3.37 Å. We note that these distances are within the range typically observed for stacked arrangements of aromatic molecules based on $\pi - \pi$ interactions.(*50*)

In the crystal structure, stacks of CH-PDI molecules are aligned parallel to the *b*-axis, and adjacent stacks are arranged relative to each other in rows parallel to the *a*-axis, as shown in the projection of the structure on to the plane perpendicular to the stacking axis in **Figure S9**. For a given row of stacks along the *a*-axis, adjacent stacks are related by the *a*-glide operation; thus, for all stacks within the row, the long axis of the rectangular cross-sectional shape is aligned in the same direction.

Adjacent rows of stacks are related to each other by the 2_1-screw operation parallel to the *c*-axis (vertical in **Figure S9**). As a consequence, for adjacent rows of stacks along the *c*-axis, the alignment of the long axis of the rectangular cross-sectional shape alternates between different directions. The interface between adjacent rows of CH-PDI molecules is parallel to the (001) plane (horizontal in **Figure S9**) and comprises interactions between cyclohexyl substituents of neighboring molecules. In the projection of the structure shown in **Figure S9**, the arrangement of

the cyclohexyl substituents at this interface may be described in terms of the interdigitation of cyclohexyl substituents from CH-PDI molecules in adjacent rows.

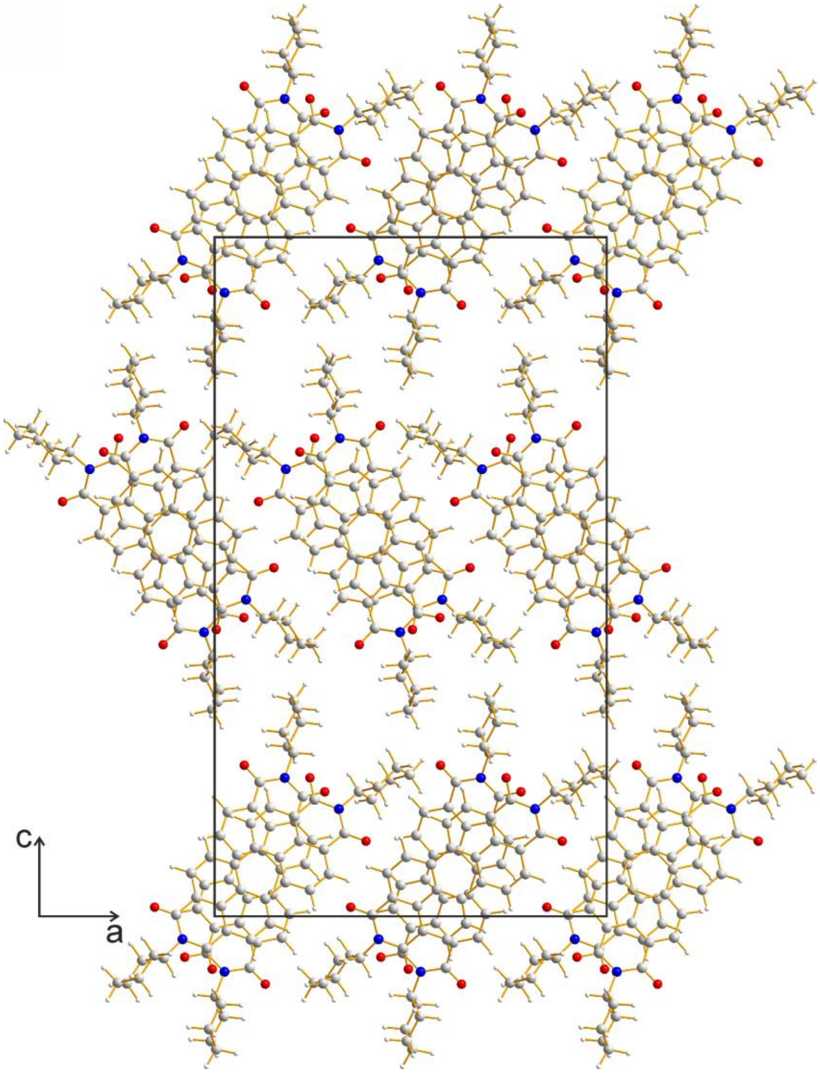

Figure S9. Crystal structure of CH-PDI determined by Rietveld refinement from PXRD data. The structure is viewed along the stacking axis (*b*-axis). Carbon atoms are shown in gray, hydrogen atoms are shown in white, nitrogen atoms are shown in blue, and oxygen atoms are shown in red.

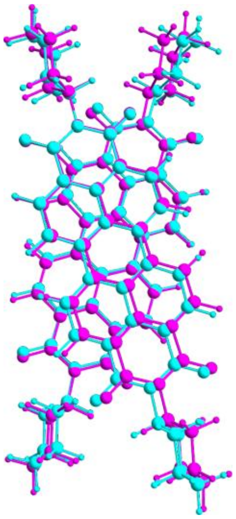

Figure S10. Comparison of the refined crystal structure of CH-PDI before and after DFT-D geometry optimization. Overlay of the asymmetric unit (comprising two crystallographically independent molecules) in the final refined crystal structure of CH-PDI (cyan) and after subjecting this crystal structure to DFT-D geometry optimization with fixed unit cell (magenta).

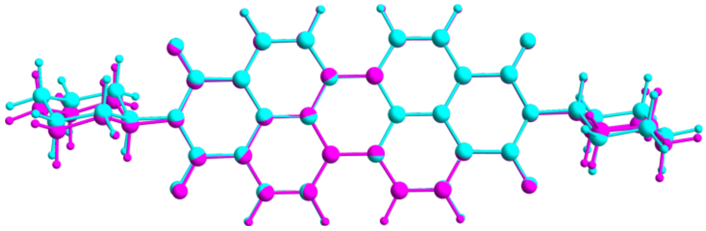

Figure S11. Comparison of the two crystallographically independent molecules in the crystal structure of CH-PDI. Overlay of the two crystallographically independent molecules (shown in cyan and magenta) in the final refined crystal structure of CH-PDI, showing the similarity of the molecular conformations.

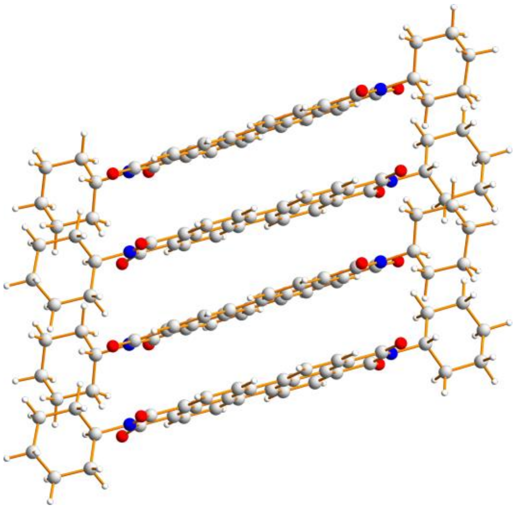

Figure S12. The molecular stacking arrangement in the crystal structure of CH-PDI. A single stack in the final refined crystal structure of CH-PDI viewed perpendicular to the stacking axis, which is vertical. Carbon atoms are shown in gray, hydrogen atoms are shown in white, nitrogen atoms are shown in blue, and oxygen atoms are shown in red.

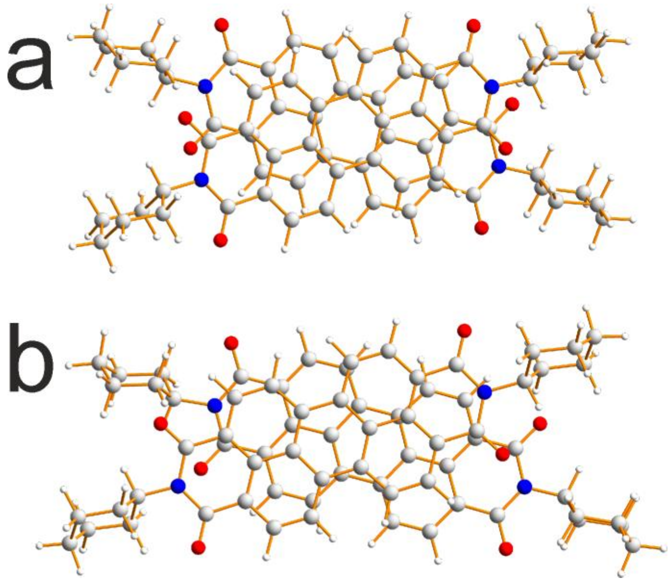

Figure S13. Structural relationship between the two crystallographically independent molecules in the crystal structure of CH-PDI. The two crystallographically independent molecules in the final refined crystal structure of CH-PDI viewed (a) along the stacking axis (*b*-axis) and (b) perpendicular to the plane of the aromatic ring system. The two crystallographically independent molecules are adjacent molecules along the stack. Carbon atoms are shown in gray, hydrogen atoms are shown in white, nitrogen atoms are shown in blue, and oxygen atoms are shown in red.

S5. Structure Determination of Polymorphs I and II of ST-PDI

S5.1. Unit Cell Determination from PXRD Data

Profile-fitting and unit cell refinement of the PXRD data for the ST-PDI sample comprising a mixture of polymorphs I and II (**Figure S14**) was carried out using the Le Bail method. For each polymorph, the initial unit cell used in the Le Bail fitting was the unit cell determined from 3D-ED data. The Le Bail fitting carried out over the range $2\theta = 5° – 30°$ [using the space group for polymorph II ($P2_1/c$) deduced from 3D-ED data] gave a good-quality fit to the PXRD data (**Figure S14**; $R_p = 1.04\%$, $R_{wp} = 1.36\%$). The final refined unit cells from the Le Bail fitting for polymorphs I and II are given in **Table S2** and are considered to be more accurate than those determined from 3D-ED data. In the Le Bail fitting, all peaks in the PXRD data are accounted for by polymorphs I and II of ST-PDI, leading to the conclusion that no other crystalline phase is present in the sample. Attempts to carry out Le Bail fitting over the range $2\theta = 3.5° – 30°$ did not give satisfactory fitting of the peaks with $2\theta < 5°$ as a consequence of the highly-sloped baseline at low 2θ (due to X-ray scattering from air) – see **Figure S15**.

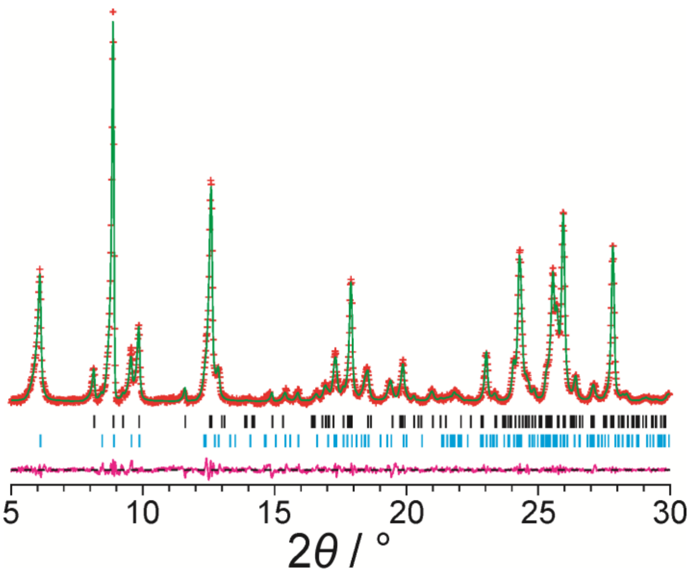

Figure S14. Profile fitting of PXRD data for ST-PDI. Results from Le Bail fitting of the PXRD data (background subtracted) for the sample containing a mixture of polymorphs I and II of ST-PDI (red + marks, experimental data; green line, calculated data; magenta line, difference plot). Tick marks show the peak positions for polymorph I (black) and polymorph II (cyan).

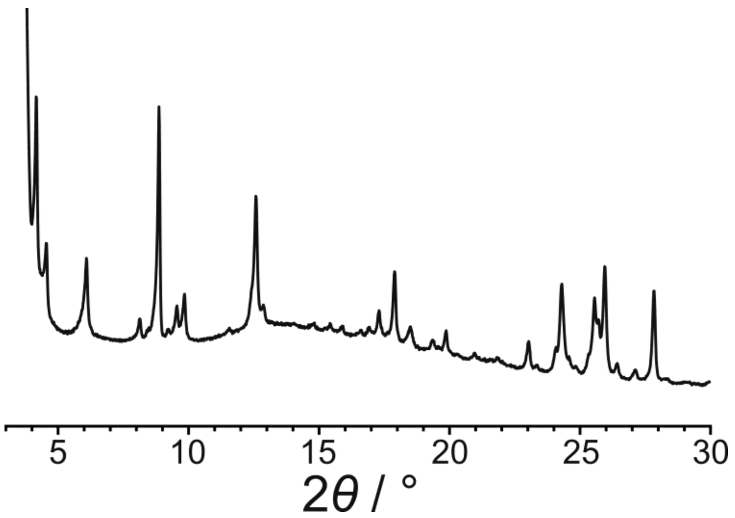

Figure S15. PXRD data for ST-PDI. PXRD data recorded for the sample containing a mixture of polymorphs I and II of ST-PDI.

Table S2. Unit cell parameters for polymorphs I and II of ST-PDI. The parameters were determined at 294 K from Le Bail fitting of PXRD data.

	Polymorph I	Polymorph II
a / Å	7.4817(5)	20.7572(18)
b / Å	11.3565(7)	19.6919(12)
c / Å	19.3050(16)	7.6277(3)
α / °	93.846(6)	90
β / °	98.650(5)	93.245(6)
γ / °	107.659(6)	90
V / Å^3	1534.10(20)	3112.8(4)

S5.2. Direct-Space Structure Solution from 3D-ED Data

Direct-space structure solution of polymorphs I and II of ST-PDI was carried out from 3D-ED data recorded for each polymorph using the direct-space genetic-algorithm (GA) strategy implemented in the program EAGER. In the direct-space GA structure-solution calculations, the geometry of the ST-PDI molecule was taken from an initial DFT-D geometry optimization. For polymorph I, structure solution was initially carried out using space group P1, with two independent molecules of ST-PDI in the unit cell. However, inspection of the best structure solutions indicated that the structure is more correctly described by space group $P\overline{1}$, with the center of each molecule located on a crystallographic inversion center. Structure solution was then carried out in space group $P\overline{1}$ with two independent half-molecules in the asymmetric unit (in each case, the complete molecule is generated by a crystallographic inversion center). Each half-molecule was defined by 8 structural variables, specifically: three rotational variables (representing rotation of the half-molecule around the inversion center) and five torsional variables (defining the conformation of the 4-heptyl substituent in the half-molecule). Thus, the total number of structural variables in the direct-space GA structure-solution calculation was 16.

For polymorph II, the space group was assigned as $P2_1/c$ from 3D-ED data, with one complete molecule of ST-PDI in the asymmetric unit. In this case, the total number of structural variables in the direct-space GA structure-solution calculation was 16, specifically: three positional variables, three rotational variables and 10 torsional variables (corresponding to 5 variable torsion angles in each of the two 4-heptyl substituents).

For each polymorph, 40 independent GA structure-solution calculations were carried out for a population of 100 trial structures. Each independent calculation started from a different randomly generated initial population, which was allowed to evolve for 200 generations with 10 mating events and 50 mutation events per generation.

For each polymorph, the trial structure giving the best fit to the 3D-ED data (i.e., the trial structure with the lowest value of R_F) in the final population of each of the 40 independent GA structure-solution calculations was examined. For polymorph I (**Figure S16a**), the structure with lowest R_F in 9 of the 40 independent GA calculations represented essentially the same structure, with a lower value of R_F than any structure in the other 31 calculations. For polymorph II (**Figure S16b**), the structure with lowest R_F obtained in 38 of the 40 independent GA calculations represented essentially the same structure, with a lower value of R_F than any structure in the other two calculations.

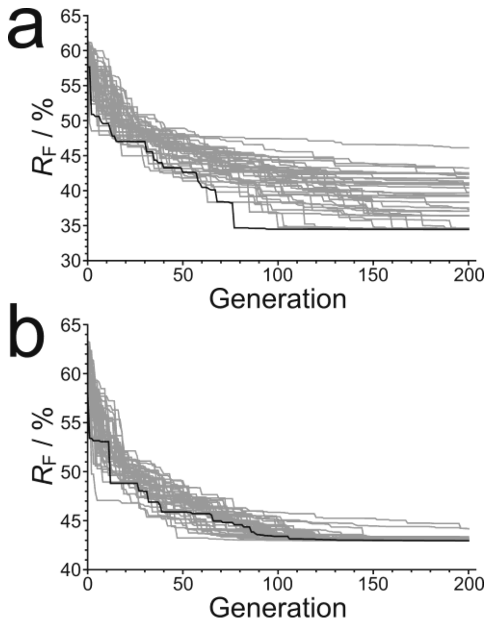

Figure S16. Evolutionary progress in the GA structure solution calculations from the 3D-ED data for polymorphs I and II of ST-PDI. Evolutionary progress in the GA structure solution calculations for (a) polymorph I and (b) polymorph II of ST-PDI. The evolution of each of the 40 independent GA calculations (each starting from a different random initial population of trial structures) is represented by a continuous grey line showing the lowest value of R_F among all 100 trial structures in the population as a function of generation number. The continuous line shown in black corresponds to the GA calculation that generates the structure with lowest R_F after 200 generations.

S5.3. Diffuse scattering observations in 3D-ED data

In a subset of the 3D-ED datasets obtained from ST-PDI, lines of diffuse scattering can be observed. **Figure S17** depicts the *hk0* plane from such a 3D-ED dataset exhibiting streaks of diffuse scattering along the b^* axis of polymorph II of ST-PDI.

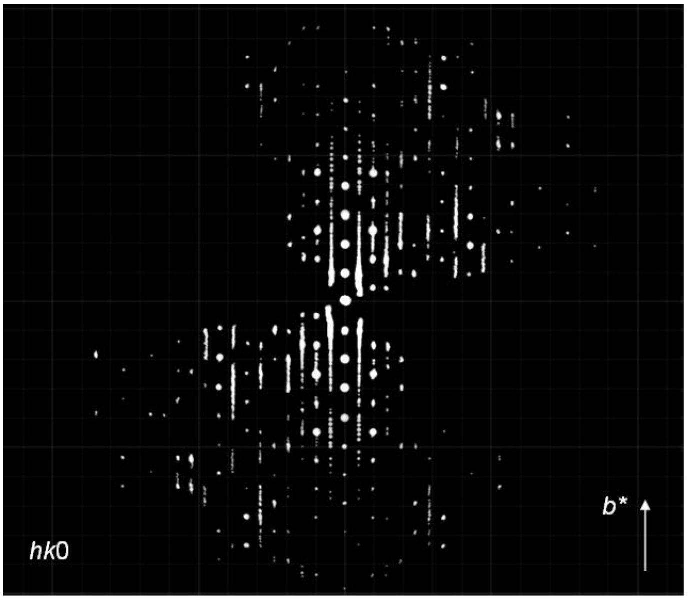

Figure S17. View of the reciprocal lattice of polymorph II of ST-PDI. The reciprocal lattice plane *hk0* extracted from a 3D-ED dataset acquired on polymorph II of ST-PDI.

S5.4. Structure refinement from 3D-ED data

Structure refinement of polymorphs I and II of ST-PDI was carried out from 3D-ED data based on the kinematic approximation using SHELXL. For each polymorph, the least squares refinement started from the best structure solution obtained in the GA structure solution calculations, with space group $P\overline{1}$ for polymorph I and space group $P2_1/c$ for polymorph II. In each case, the unit cell determined from profile fitting of PXRD data was used for the structure refinement from the 3D-ED data (see **Table S2**), as it is considered more reliable than the unit cell determined directly from the 3D-ED data. Geometric restraints based on standard molecular geometries were included in the refinement calculations. Details of the restraints are given in the crystallographic information files (CIFs) for the structures of polymorphs I and II of ST-PDI deposited in the CSD. The refinements converged with R1 residuals of 0.243 for polymorph I and 0.280 for polymorph II of ST-PDI. More details are given in **Tables S3-S4**.

Table S3. Details of the 3D electron diffraction data and structure refinement for polymorph I of ST-PDI. Unit cell parameters were determined from Le Bail fitting of PXRD data at 294 K.

Crystal system	Triclinic
Space group	$P\bar{1}$ (No. 2)
a / Å	7.4817(5)
b / Å	11.3565(7)
c / Å	19.3050(16)
α / °	93.846(6)
β / °	98.650(5)
γ / °	107.659(6)
V / Å^3	1534.1(2)
T / K	100
λ / Å	0.0251
Exposure time per frame / s	0.5
Tilt range	–27.86° to +44.36°
Resolution / Å	0.80
Completeness / %	35.8
R_{int}	0.105
R1	0.243
wR2	0.489
Number of symmetry independent reflections	2207
Number of parameters	177
Number of restraints	50

Table S4. Details of the 3D electron diffraction data and structure refinement for polymorph II of ST-PDI. Unit cell parameters were determined from Le Bail fitting of PXRD data at 294 K.

Crystal system	Monoclinic
Space group	$P2_1/c$ (No. 14)
a / Å	20.7572(18)
b / Å	19.6919(12)
c / Å	7.6277(3)
α / °	90
β / °	93.245(6)
γ / °	90
V / Å^3	3112.8(4)
T / K	100
λ / Å	0.0251
Exposure time per frame / s	0.5
Tilt range	–52.29° to +62.14°
Resolution / Å	0.88
Completeness / %	60.9
R_{int}	0.157
R1	0.280
wR2	0.549
Number of symmetry independent reflections	2925
Number of parameters	177
Number of restraints	51

S5.5. DFT-D Calculations

Periodic DFT-D geometry optimization calculations were carried out (using the methodology described in **Materials and Methods Section 4.9.1**) to assess the relative energies of the crystal structures of polymorphs I and II of ST-PDI obtained in the final refinements from 3D-ED data (using the unit cell determined from PXRD data for each polymorph). DFT-D geometry optimization was carried out initially with fixed unit cell and then with relaxation of the unit cell. For the DFT-D geometry optimizations with fixed unit cell, the root-mean squared deviation (RMSD) in the atomic positions (non-H atoms) between the final refined crystal structure from 3D-ED data and the structure following DFT-D geometry optimization with fixed unit cell is 0.11 Å for both polymorphs I and II. These small values of RMSD confirm that the crystal structures determined from 3D-ED data are very close to minima on the energy landscape. From these calculations, polymorph I is lower in energy than polymorph II by 2.71 kJ mol^{-1} (per mole of ST-PDI molecules).

In the structures obtained following DFT-D geometry optimization with relaxation of the unit cell, the energies of polymorphs I and II become closer, with polymorph I lower in energy than polymorph II by only 0.35 kJ mol^{-1} (per mole of ST-PDI molecules). The unit cells obtained in the DFT-D geometry optimization calculations following unit cell relaxation are shown in **Table S5**. The RMSDs in the atomic positions (non-H atoms) between the final refined structure from the 3D-ED data and the structure following DFT-D geometry optimization with relaxation of the unit cell are 0.31 Å for polymorph I and 0.40 Å for polymorph II.

Table S5. Unit cell parameters for polymorphs I and II of ST-PDI following DFT-D geometry optimization with unit cell relaxation. The unit cell parameters are given for polymorphs I and II of ST-PDI following DFT-D geometry optimization with relaxation of the unit cell.

	Polymorph I	Polymorph II
a / Å	7.3848	20.3955
b / Å	11.0633	19.3059
c / Å	18.6584	7.36319
α / °	91.3729	90
β / °	104.456	92.212
γ / °	100.217	90
V / Å^3	1448.99	2897.11

S5.6. Solid-State ^{13}C NMR Spectroscopy

High-resolution solid-state ^{13}C NMR data were recorded for the same sample of ST-PDI studied by PXRD (comprising a mixture of polymorphs I and II) using ^{1}H→^{13}C cross-polarization (CP) with high-power ^{1}H decoupling and magic-angle sample spinning. More details of the experimental methodology are given in **Materials and Methods Section 4.8**. The high-resolution solid-state ^{1}H→^{13}C CP NMR spectrum (**Figure S18**) contains isotropic peaks in the range *ca*. 100 – 170 ppm corresponding to ^{13}C environments in the aromatic ring system of ST-PDI and peaks in the range *ca*. 15 – 60 ppm corresponding to ^{13}C environments in the 4-heptyl substituents. Given the large number of ^{13}C environments in the aromatic ring system, there is significant peak overlap between 100 ppm and 170 ppm, and the region of the spectrum (*ca*. 10 – 60 ppm) containing isotropic peaks for the 4-heptyl substituents is more informative for elucidating structural information. The isotropic peak for the N–^{13}CH environment in the 4-heptyl substituent at *ca*. 58 ppm shows more than two isotropic peaks, which is fully consistent with the presence of both polymorphs I and II in the sample (we recall that the crystal structure of each polymorph has two independent ^{13}C environments of this type, corresponding to the two independent 4-heptyl substituents in the asymmetric unit).

For the crystal structures of polymorphs I and II of ST-PDI obtained by refinement from the 3D-ED data, calculation of the isotropic ^{13}C NMR chemical shifts was carried out using DFT-GIPAW methodology to allow comparison to the experimental solid-state ^{13}C NMR data. More details of the DFT-GIPAW calculations are given in **Materials and Methods Section 4.9.2**. The DFT-GIPAW calculated solid-state ^{13}C NMR spectra for polymorphs I and II of ST-PDI are shown in **Figure S19** together with the experimental solid-state ^{1}H→^{13}C CP NMR spectrum (also shown in **Figure S18**) of the sample comprising a mixture of polymorphs I and II. From these results, we note firstly that the calculated solid-state ^{13}C NMR spectra for polymorphs I and II of ST-PDI share significant similarity, which is fully consistent with the presence of stacks of ST-PDI that are essentially isostructural in each polymorph. The structural differences between the polymorphs

primarily concern the long-range arrangement of the stacks relative to each other, whereas ^{13}C NMR chemical shifts are sensitive to the local structural properties, which is reflected in the similarity of the calculated values of the isotropic ^{13}C NMR chemical shifts for each polymorph. Secondly, it is clear that there is good agreement between the calculated solid-state ^{13}C NMR data for polymorphs I and II of ST-PDI and the experimental solid-state ^{13}C NMR data, which is fully consistent with the sample comprising a mixture of polymorphs I and II of ST-PDI. In principle, it may be viable to assess the relative amounts of polymorphs I and II of ST-PDI within the sample by comparing the relative intensities of specific features in the experimental solid-state ^{13}C NMR spectrum that may be assigned (from the calculated solid-state ^{13}C NMR data) as due to either polymorph I or polymorph II. However, in the present case, this type of analysis is not justified for two reasons. First, due to the significant similarity of the ^{13}C NMR chemical shifts for polymorphs I and II, individual peaks in the experimental spectrum cannot necessarily be assigned unambiguously to a given polymorph. Second, as the experimental solid-state ^{13}C NMR spectrum was recorded using the ^{1}H→^{13}C CP technique, the relative intensities of individual peaks depend both on the relative amounts of the two polymorphs and on the relative CP efficiencies for different ^{13}C environments in each polymorph; as a consequence (and in the absence of a detailed experimental study to determine the relative CP efficiencies for each ^{13}C environment in each polymorph), analysis of the relative intensities of peaks in the experimental solid-state ^{13}C NMR spectrum cannot be used as a quantitative measure of the relative amounts of the two polymorphs present in the sample.

High-resolution solid-state ^{13}C NMR spectra were also recorded using the dipolar-dephasing technique(*60*, *61*) (**Figure S20**) with different values of the dipolar dephasing delay. This technique for measuring solid-state ^{13}C NMR spectra provides qualitative insights into the occurrence of dynamic processes in organic materials.(*62–67*) Specifically, for ^{13}C nuclei directly bonded to ^{1}H nuclei, a signal is observed in the dipolar-dephasing ^{13}C NMR spectrum only if the $^{13}CH_n$ ($n = 1 – 3$) group is involved in a dynamic process. In addition, signals are also observed in

the dipolar-dephasing ^{13}C NMR spectrum for ^{13}C nuclei that are not directly bonded to ^{1}H nuclei (e.g., for some of the aromatic ^{13}C environments in ST-PDI). For the 4-heptyl substituent, the only peaks observed in the dipolar-dephasing solid-state ^{13}C NMR spectra (**Figure S20**) are those for the CH_3 groups (arising from rapid rotation of CH_3 groups around the C–CH_3 bond). The absence of signals for the other $^{13}CH_n$ environments of the 4-heptyl substituents in the dipolar-dephasing ^{13}C NMR spectra suggests that no significant conformational dynamic processes occur for these substituents.

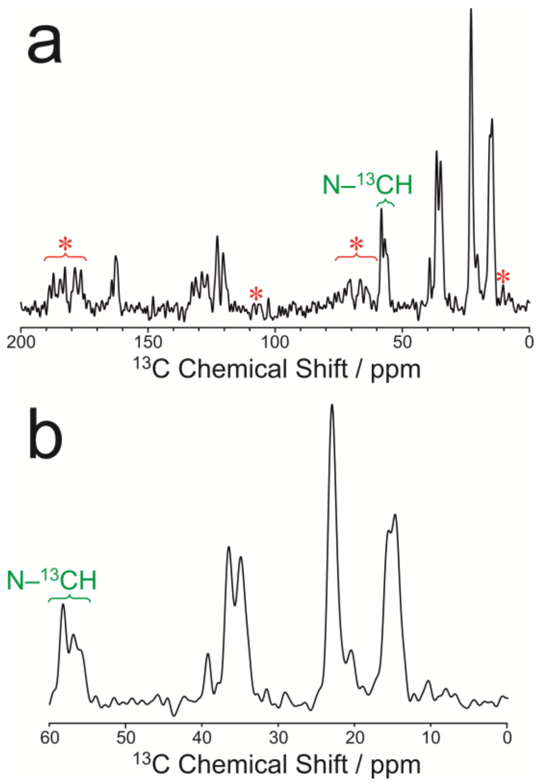

Figure S18. High-resolution solid-state ^{13}C NMR spectra from ST-PDI. (a) Solid-state $^{1}H \rightarrow ^{13}C$ CP NMR spectrum recorded for the sample comprising a mixture of polymorphs I and II of ST-PDI (spinning sidebands are marked with red asterisks). (b) The region of the $^{1}H \rightarrow ^{13}C$ CP NMR spectrum containing isotropic peaks for the ^{13}C environments in the 4-heptyl substituent.

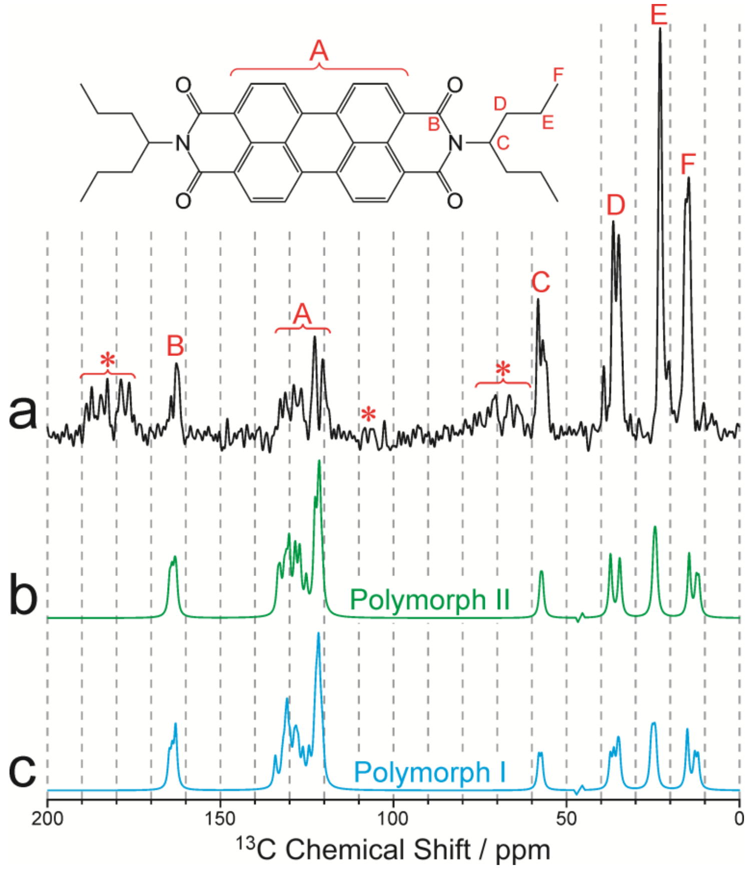

Figure S19. Comparison of experimental and simulated solid-state ^{13}C NMR spectra for ST-PDI. (a) Experimental ^{1}H→^{13}C CP NMR spectrum recorded for the sample comprising a mixture of polymorphs I and II of ST-PDI (black), with spinning sidebands marked with red asterisks. Assignments of peaks to specific ^{13}C environments in the ST-PDI molecule are indicated. (b, c) Simulated solid-state ^{13}C NMR spectra for (b) polymorph I and (c) polymorph II, based on the isotropic ^{13}C chemical shifts determined from the DFT-GIPAW calculations for each polymorph, with the same intensity and linewidth used for each ^{13}C site in the structure (therefore assuming that the efficiency of ^{1}H→^{13}C CP is the same for each ^{13}C site). The method for referencing the DFT-GIPAW calculated values of the isotropic ^{13}C chemical shifts is described in **Materials and Methods Section S4.9.2**.

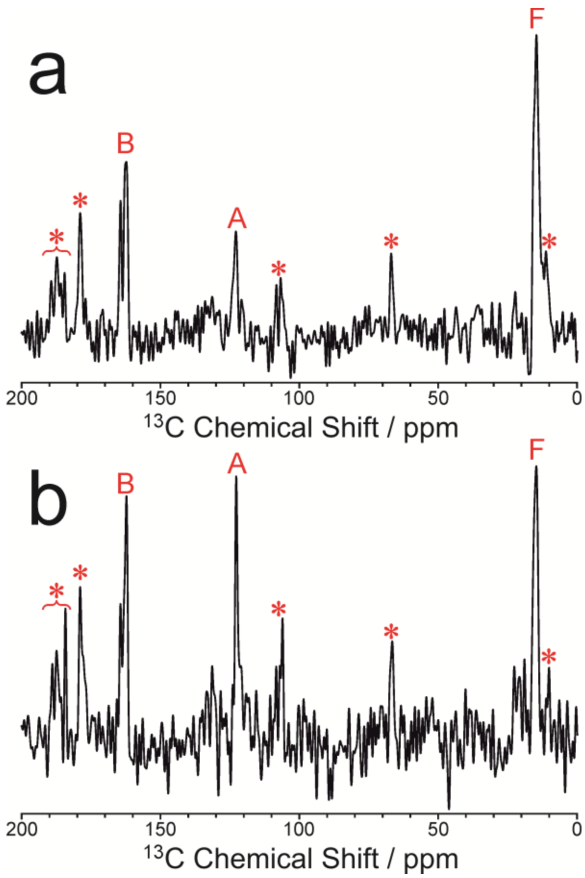

Figure S20. High-resolution solid-state ^{13}C NMR spectra recorded for ST-PDI with different dipolar dephasing delays. Solid-state ^{13}C NMR spectra of the sample comprising a mixture of polymorphs I and II of ST-PDI, recorded using rotor-synchronized dipolar dephasing with dephasing delays of (a) 100 μs and (b) 200 μs. Spinning sidebands are marked with red asterisks. Assignments of peaks to specific ^{13}C environments in the ST-PDI molecule (see **Figure S19**) are indicated.

S5.7. Discussion of the Crystal Structures of Polymorphs I and II of ST-PDI

The crystal structures of polymorphs I and II of ST-PDI (**Figure S21**) both contain stacks of molecules, which are parallel to the *a*-axis in polymorph I and parallel to the *c*-axis in polymorph II. In polymorph I (space group $P\overline{1}$), there are two independent molecules, which represent adjacent molecules along the stack; the center of each molecule coincides with a crystallographic inversion center (thus, the asymmetric unit comprises two half-molecules). In polymorph II (space group $P2_1/c$), there is one molecule in the asymmetric unit and adjacent molecules along the stack are related by the *c*-glide operation.

In spite of the different symmetry properties of polymorphs I and II, the stacks of ST-PDI molecules in each polymorph are essentially isostructural (see **Figures S21-S23**). The RMSD in atomic positions between the stacks in polymorphs I and II is 0.17 Å for all non-H atoms in the molecule (including the 4-heptyl substituents) and 0.10 Å for the non-H atoms of the aromatic ring system. In each polymorph, the ST-PDI molecules are tilted relative to the stacking axis (see **Figure S22**), with an angle between the normal to the plane of the aromatic ring system and the stacking axis of 22.6° and 23.5° for the two independent molecules in polymorph I, and 23.3° for the molecule in polymorph II.

As described above for the crystal structure of CH-PDI, the stacks in both polymorphs I and II of ST-PDI have an approximately rectangular cross-sectional shape (when projected on to the plane perpendicular to the stacking axis), and one axis of the rectangular cross-sectional shape is significantly longer than the other axis. Again, it is convenient to discuss the relative positions and orientations of adjacent stacks in the crystal structure in terms of the packing of the rectangular cross-sectional shapes relative to each other in two dimensions.

For polymorph I, each molecule is located on a crystallographic inversion center, and the distance between the centers of adjacent molecules along the stack (*a*-axis) is 3.74 Å, which corresponds to half the unit cell translation along the *a*-axis; the perpendicular distance between the planes of

the aromatic ring systems of adjacent molecules in the stack is *ca*. 3.44 Å. For polymorph II, adjacent molecules along the stack are related by the *c*-glide symmetry operation, with a distance of 3.81 Å between the centers of adjacent molecules; the perpendicular distance between the planes of the aromatic ring systems of adjacent molecules in the stack is *ca*. 3.50 Å. The distance between the centers of adjacent molecules along the stacks in each polymorph of ST-PDI is slightly higher than in the structure of CH-PDI, and the perpendicular distance between the planes of the aromatic ring systems of adjacent molecules in each polymorph of ST-PDI is also slightly higher than in the structure of CH-PDI.

In both polymorphs I and II of ST-PDI, the 4-heptyl substituents have very similar conformational properties (**Figure S25**) in which the two propyl branches of the substituent are oriented above and below the plane of the aromatic ring system (see **Figures S22** and **S25**). For all the 4-heptyl substituents in the two polymorphs, one branch (denoted Branch 1 in **Figure S25**) is more extended than the other branch (denoted Branch 2 in **Figure S25**) as a result of different values of the N–C–C–C torsion angle, which is in the range 140° to 149° for the more extended branch and in the range 60° to 77° for the less extended branch.

In both polymorphs I and II of ST-PDI, there is an alternation in the orientations of adjacent molecules along the stack (see **Figures S23-S24**), with the long molecular axis (defined by the intramolecular N···N vector) differing in orientation between adjacent molecules by 40.5° for polymorph I and by 39.2° for polymorph II. Alternation of the molecular orientations in this manner allows the aromatic ring systems to form a favorable π-stacking arrangement, while avoiding unfavorable steric interactions between the 4-heptyl substituents of adjacent molecules. We note that similar features of the packing of molecules along the stacks are observed in the structure of CH-PDI (**Section S4.4**).

While the individual stacks of molecules in polymorphs I and II of ST-PDI are essentially isostructural, as discussed above, the arrangement of the stacks relative to each other differs

between the two polymorphs, which is clear from comparison of the two structures viewed along the stacking axis (**Figure S21**). When viewed in projection on to the plane perpendicular to the stacking axis in **Figure S21**, each structure may be described in terms of rows of stacks aligned parallel to the long axis of the rectangular cross-sectional shape of each stack; the direction of these rows corresponds to the projection of the unit cell vector $\boldsymbol{b} + \boldsymbol{c}$ in polymorph I and the projection of the unit cell vector $\boldsymbol{a}$ in polymorph II (in each case, these rows are horizontal in **Figure S21**). Relative to a given row of this type (e.g., the bottom row for each polymorph in **Figure S21**), the position of the adjacent row (moving vertically upwards in **Figure S21**) is essentially identical in each polymorph. To quantify this structural similarity, in the projections of the structures of polymorph I and polymorph II in **Figure S21**, the repeat distance (d_1) along a row of molecules in the horizontal direction (represented by cyan arrows) is 20.78 Å for polymorph I and 20.72 Å for polymorph II. The relative positions of the molecules in the adjacent row (vertically upwards in **Figure S21**) are defined by the distance d_2 (represented by green arrows) and the angle ψ (the angle between the cyan and green arrows), which have the values d_2 = 10.82 Å and ψ = 65.7° for polymorph I, and d_2 = 10.84 Å and ψ = 65.3° for polymorph II.

However, the main structural difference between polymorphs I and II concerns the position of the next row of stacks (again moving vertically upwards in **Figure S21**), for which the displacement relative to the row below (defined by the magenta arrows in **Figure S21**) continues in the <u>same direction</u> in polymorph I but continues in the <u>opposite direction</u> in polymorph II. Thus, in polymorph I, the positional relationship between adjacent rows of stacks propagates in the same direction throughout the structure (as shown by the same direction of the green and magenta arrows in **Figure S21a**). In contrast, for polymorph II, the positional relationship between adjacent rows of stacks alternates between two sets of positions on moving through the structure (as shown by the zig-zag arrangement of the green and magenta arrows in **Figure S21b**). Thus, the positional relationship between any two adjacent rows is essentially identical in polymorphs I and II, but the two polymorphs differ in the position of the next row, either leading to a unidirectional offset

(polymorph I) or a zig-zag offset (polymorph II) in the relative positions of adjacent rows of stacks through the structure.

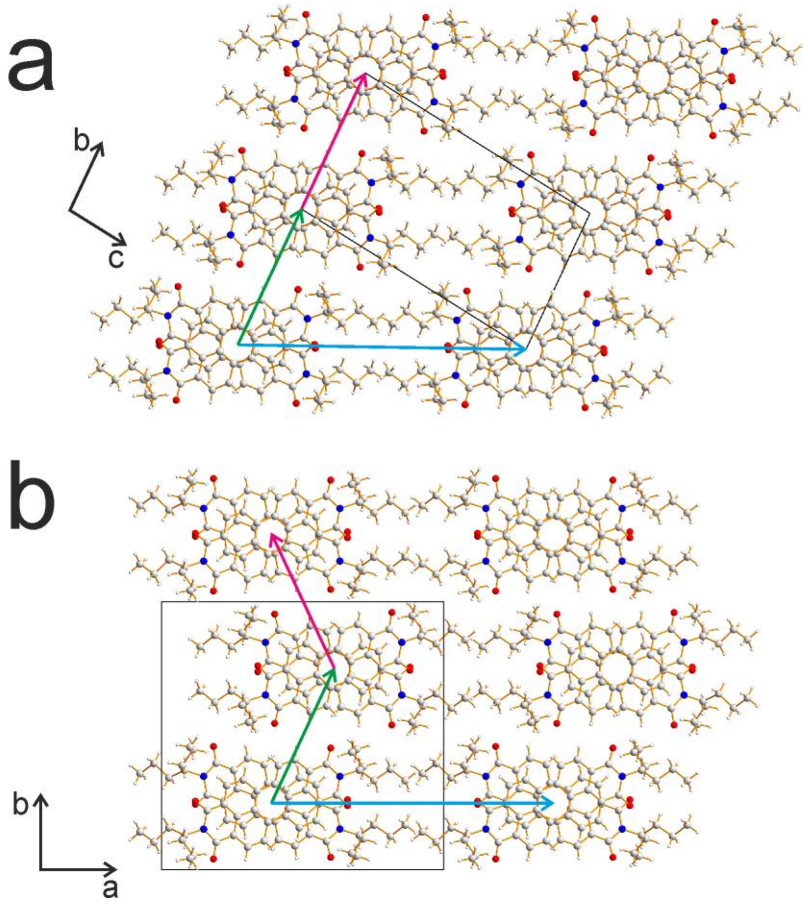

Figure S21. Crystal structures of polymorphs I and II of ST-PDI. The final refined structures of (a) polymorph I and (b) polymorph II of ST-PDI viewed along the stacking axis in each case. The arrows indicate vectors (in the plane perpendicular to the stacking axis) between neighboring stacks, as discussed in the text. Carbon atoms are shown in gray, hydrogen atoms are shown in white, nitrogen atoms are shown in blue, and oxygen atoms are shown in red.

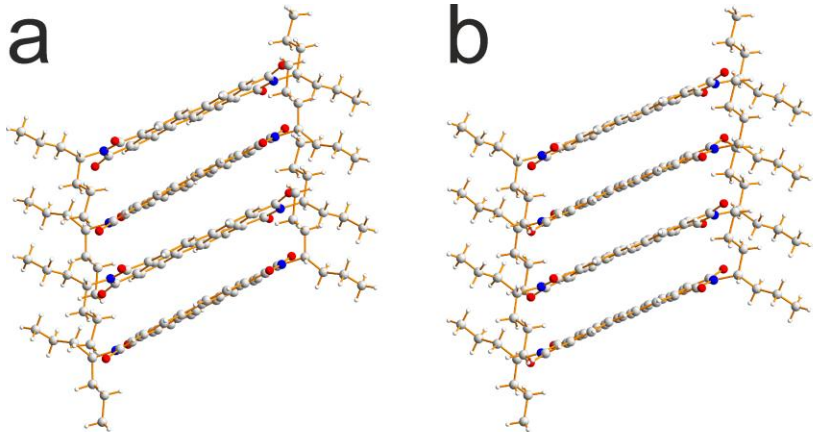

Figure S22. The molecular stacking arrangements in the crystal structures of polymorphs I and II of ST-PDI. The stacks of ST-PDI molecules, viewed perpendicular to the stacking axis, in the structures of (a) polymorph I and (b) polymorph II of ST-PDI. The RMSD in atomic positions between the stacks in polymorphs I and II is 0.10 Å for all non-H atoms in the aromatic ring system and 0.17 Å for all non-H atoms in the molecule. Carbon atoms are shown in gray, hydrogen atoms are shown in white, nitrogen atoms are shown in blue, and oxygen atoms are shown in red.

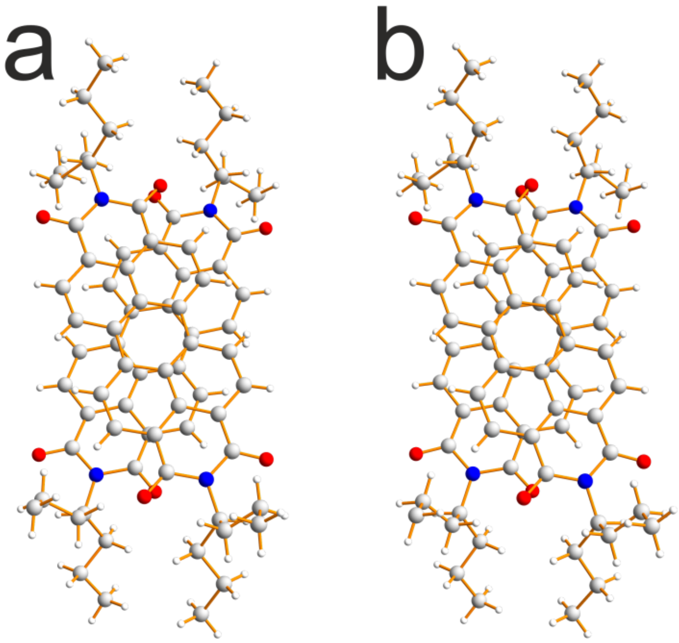

Figure S23. Structural relationship between adjacent molecules along the stacks in the crystal structures of polymorphs I and II of ST-PDI viewed parallel to the stacking axis. The images show the stacks in (a) polymorph I and (b) polymorph II of ST-PDI, viewed along the stacking axis in each case. Carbon atoms are shown in gray, hydrogen atoms are shown in white, nitrogen atoms are shown in blue, and oxygen atoms are shown in red.

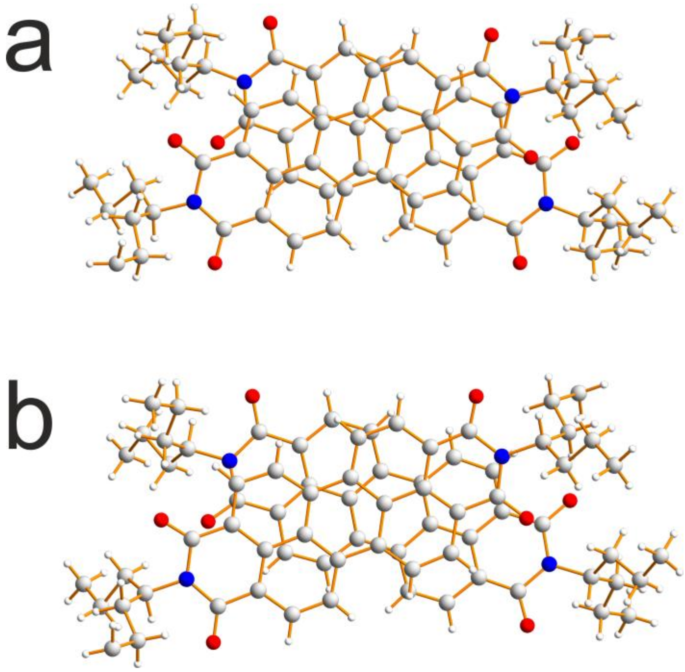

Figure S24. Structural relationships between adjacent molecules along the stacks in the crystal structures of polymorphs I and II of ST-PDI viewed perpendicular to the plane of the aromatic ring systems. The images show the adjacent molecules along the stack of (a) polymorph I and (b) polymorph II of ST-PDI. In each case, the direction of view is perpendicular to the plane of the aromatic ring systems. Given the near-identical structural relationship between adjacent molecules along the stacking axis, the two polymorphs would be expected also to exhibit similar optoelectronic properties. Carbon atoms are shown in gray, hydrogen atoms are shown in white, nitrogen atoms are shown in blue, and oxygen atoms are shown in red.

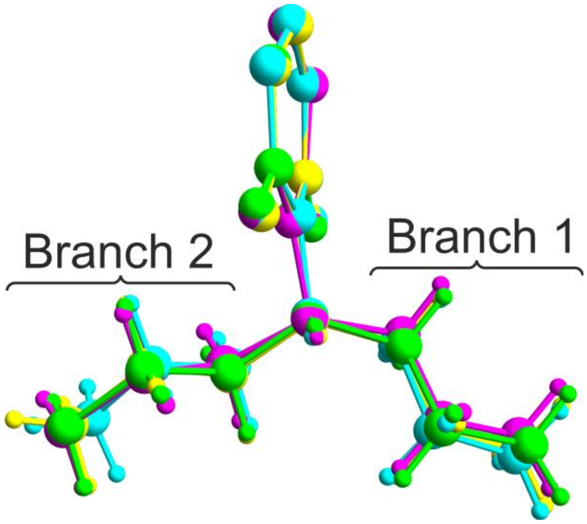

Figure S25. Comparison of the conformations of the 4-heptyl substituents in polymorphs I and II of ST-PDI. Overlay of the four 4-heptyl substituents in the structures of polymorph I (cyan and magenta) and polymorph II (yellow and green) of ST-PDI. In each case, the aromatic ring directly bonded to the 4-heptyl substituent is also shown. Branches 1 and 2 of the 4-heptyl substituent, as discussed in the text, are also indicated.

S5.8. Comparison to Crystal Structures of Other PDI Materials

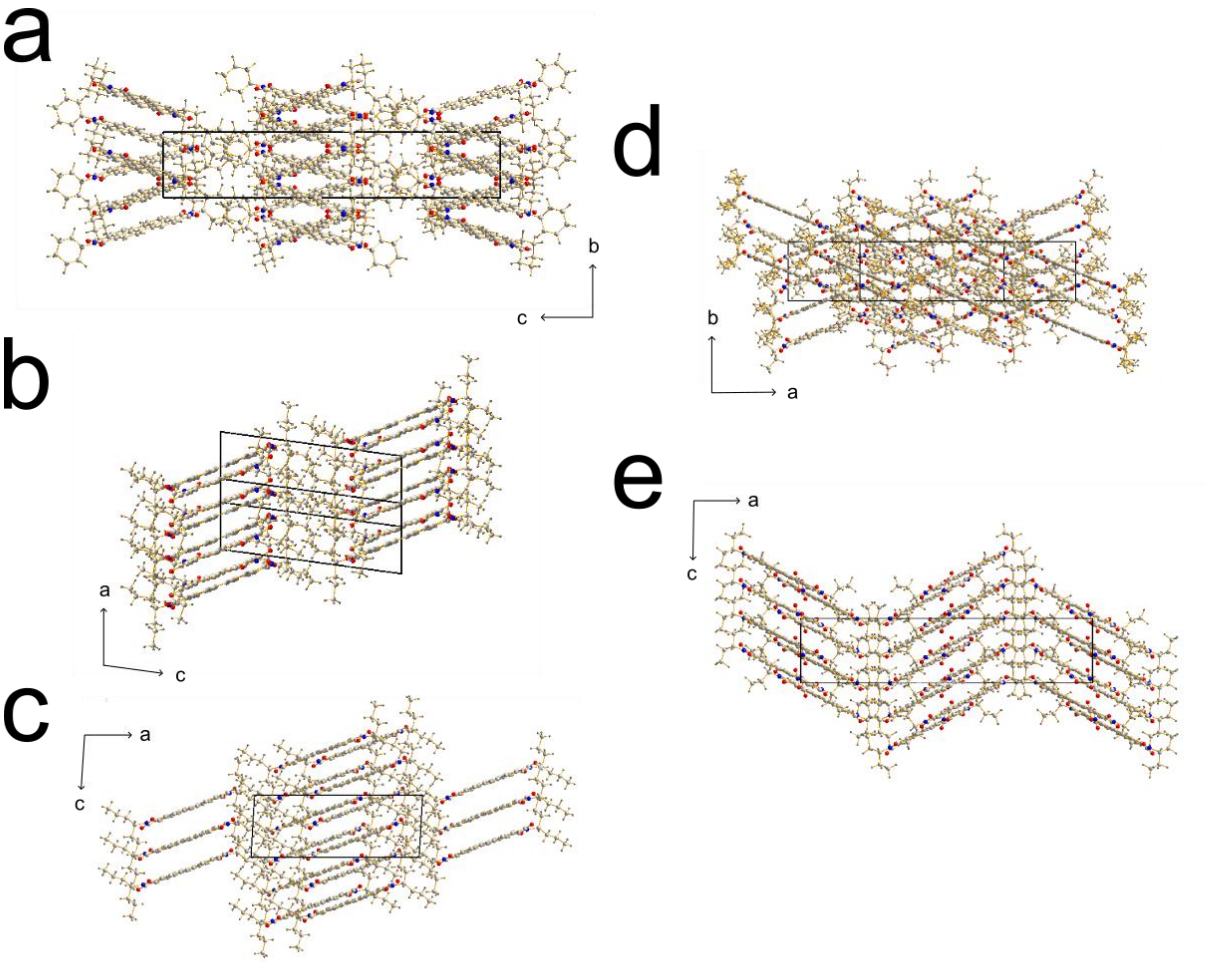

Figure S26. Visualization of the molecular packing arrangements in the crystal structures of different PDI derivatives. (a) Crystal structure of CH-PDI viewed along [100]. (b) Crystal structure of polymorph I of ST-PDI viewed along [230]. (c) Crystal structure of polymorph II of ST-PDI viewed along [010]. (d) Crystal structure of the reported N,N′-bis(3-pentyl) PDI structure (*68*) viewed along [$\bar{1}$03]. (e) Crystal structure of the reported nitro-PDI structure (*69*) viewed along [010]. Carbon atoms are shown in gray, hydrogen atoms are shown in white, nitrogen atoms are shown in blue, and oxygen atoms are shown in red.

S6. SED of Nanobelts Indexed to Polymorphs I and II of ST-PDI

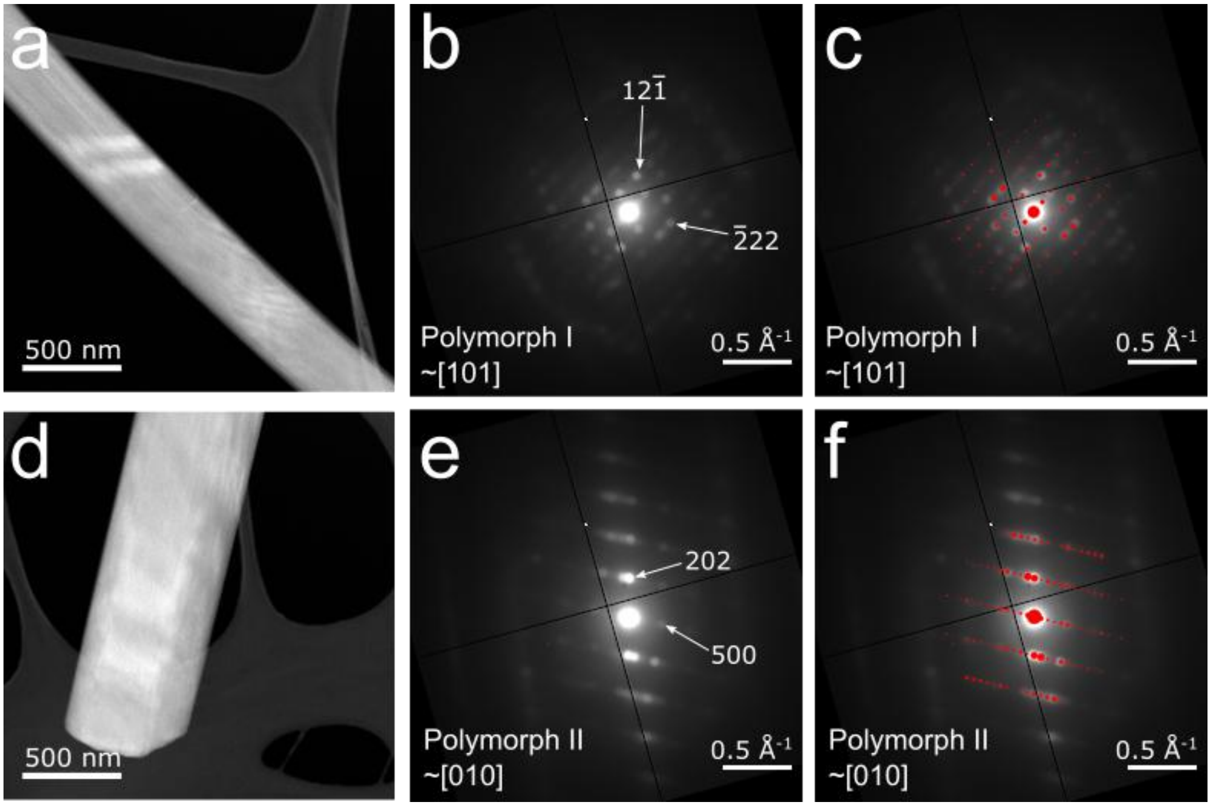

Figure S27. Indexation of SED data to polymorphs I and II of ST-PDI. (a) ADF-STEM and (b) corresponding average diffraction pattern for a nanobelt indexed to polymorph I of ST-PDI near the [101] zone axis orientation. (c) A simulated electron diffraction pattern for polymorph I of ST-PDI along [101] overlaid on the average diffraction pattern showing matched spacings and relative intensities. (d) ADF-STEM and (e) corresponding average diffraction pattern for a nanobelt indexed to polymorph II of ST-PDI near the [010] zone axis orientation. (f) A simulated electron diffraction pattern for polymorph II of ST-PDI along [010] overlaid on the average diffraction pattern showing matched spacings and relative intensities.

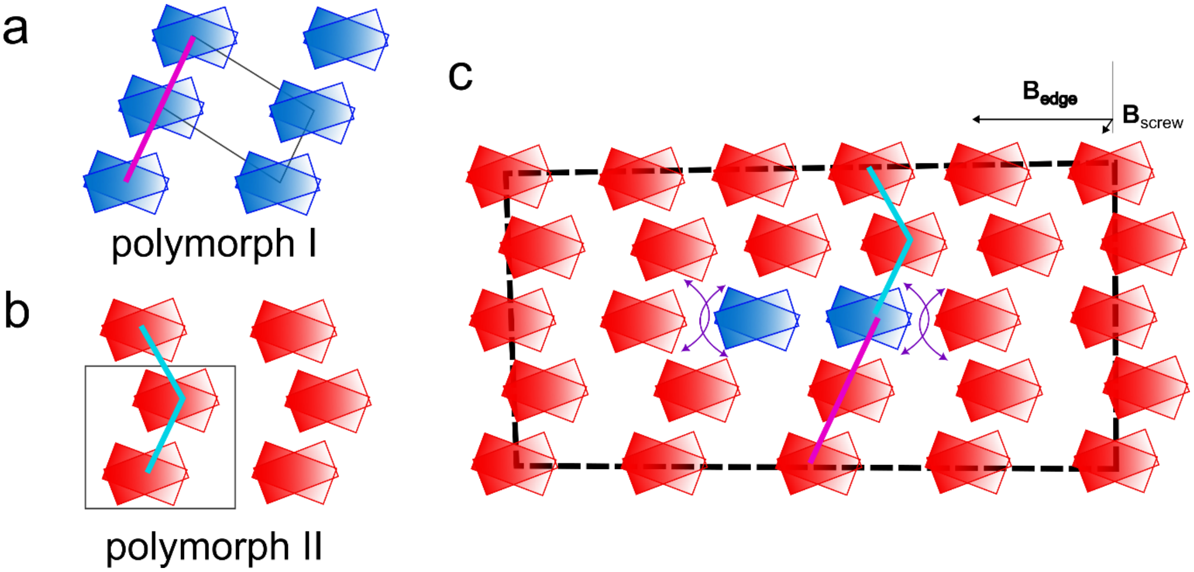

Figure S28. A simplified model of dislocations in ST-PDI arising from polymorph intergrowth. (a) Schematic illustration of the crystal structure of polymorph I of ST-PDI (as also shown in **Figure S21a**). The typical unidirectional arrangement of adjacent rows of stacks in polymorph I is highlighted by the magenta line. (b) Schematic illustration of the crystal structure of polymorph II of ST-PDI (as also shown in **Figure S21b**). The zig-zag arrangement of adjacent rows of stacks in polymorph II is highlighted by the cyan lines. (c) Model of a mixed dislocation arising from local mis-stacking along the b^* axis of polymorph II of ST-PDI. A Burgers circuit is indicated by the black dashed line. Near the core, alternation of the unidirectional and zig-zag arrangements of stacks can be accommodated by local displacements (strain), likely avoiding steric clashes further through rotation of the π-stacked molecules. A screw component is indicated out of the page to ensure the parallel alignment of π-stacked molecules is preserved away from the defect. Displacement perpendicular to the stack axis (edge component) requires displacement also parallel to the stack axis (screw component) to preserve end-to-end interactions three-dimensionally (away from the dislocation core) due to the inclined orientation relative to the stack axis.

S7. Linear Defects Across ST-PDI Nanobelts

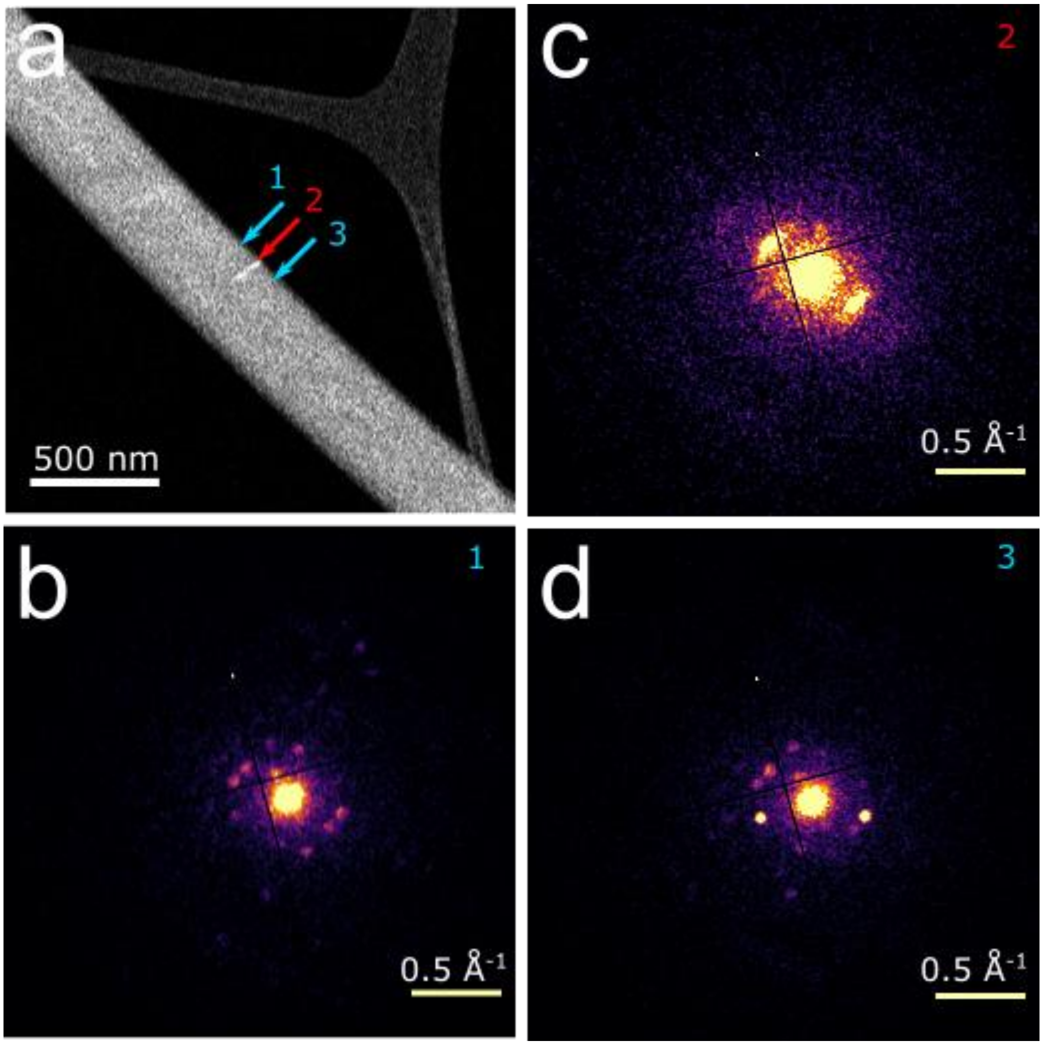

Figure S29. A linear defect at the edge of a nanobelt indexed to polymorph I of ST-PDI. The same nanobelt is also shown in **Figure S27a-c**. (a) A VDF image formed at diffuse scattering observed in (c). The diffuse scattering appears at the defect, labelled as position 2. Diffraction patterns (b) immediately before the linear defect (position 1) and (d) immediately after the linear defect (position 3) visible at position 2 in (a) show a consistent single-crystal orientation as observed by the identical locations of spots in the diffraction pattern.

S8. Electron Energy Loss Spectroscopy (EELS)

S8.1. EELS at Optical Transition Energies

Electron beam irradiation during STEM imaging and EELS is known to cause radiolytic damage in molecular crystals. When acquiring EELS upon scanning across a PDI nanobelt (**Figure S30**, left) peaks between 2-3 eV (optical transition energies) were observed in positions just outside the nanobelt ('aloof'). These were significantly damped after scanning across the nanobelt and repeating a measurement in the same location. Instead, spectra were acquired from aloof probe positions in a line scan parallel to the long axis of the nanobelt. **Figure S30** (middle, right) depicts a sum spectrum from a single line spectrum over 500 nm along a nanobelt with an improved signal-to-noise relative to a single local-area scan, evidence of preserved signal throughout the scan. This procedure was also extended to multi-line scans starting from vacuum and progressing into the material (**Figures S31-S32**). Such spectrum images consisting of sequential line scans exhibited sharp peaks including the first line scan within the nanobelt and deteriorated thereafter, enabling separation of spectra with sufficient signal-to-noise ratio in post-acquisition processing.

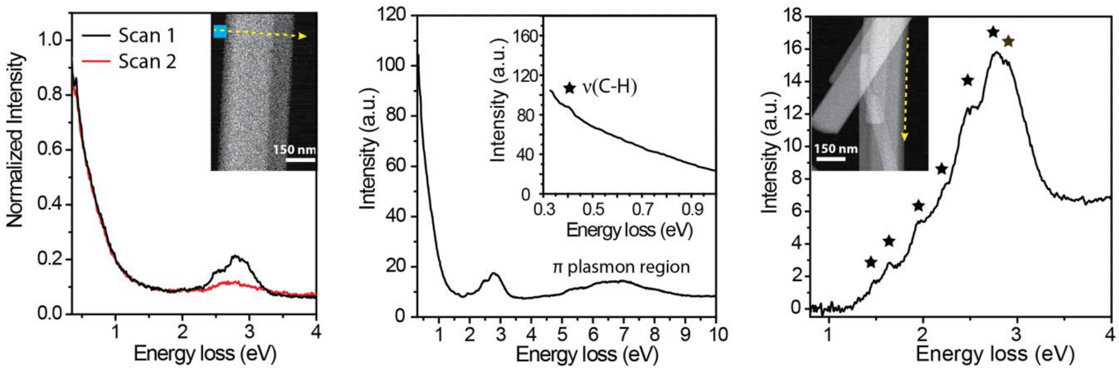

Figure S30. Optimizing data acquisition in EELS at optical energies. (Left) Repeated EELS acquisitions across a PDI nanobelt. The intensity at the optical transition energies decreased significantly after a single scan. (Middle, right) Summed spectrum and background subtracted spectrum from 500 nm length along nanobelt with the electron beam just outside ('aloof') the nanobelt. The resulting spectrum exhibits numerous peak features through signal averaging.

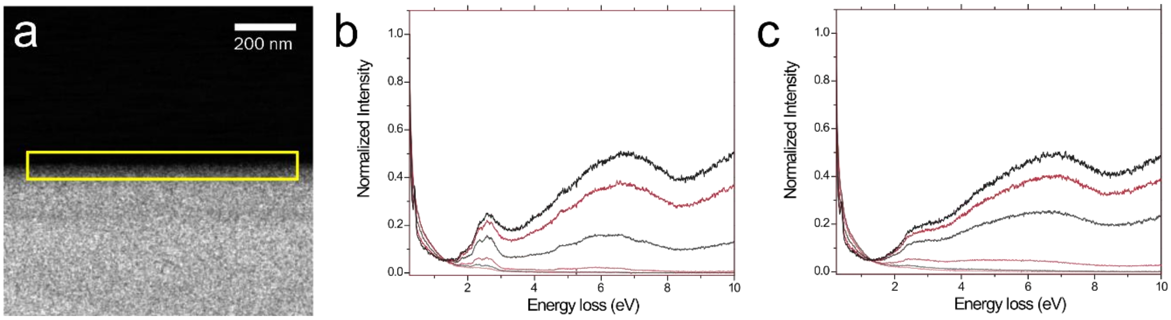

Figure S31. Effects of cumulative electron beam damage on EELS at optical energies. (a) Overview ADF-STEM micrograph showing the location of a spectrum image consisting of line profiles approaching the edge of a PDI nanobelt. The scan was initiated from the region furthest from the nanobelt in vacuum (black intensity). Spectra from (b) the first pass and (c) the second pass showing degradation of the signal after one exposure but retained peak features in the first scan into the edge of the nanobelt.

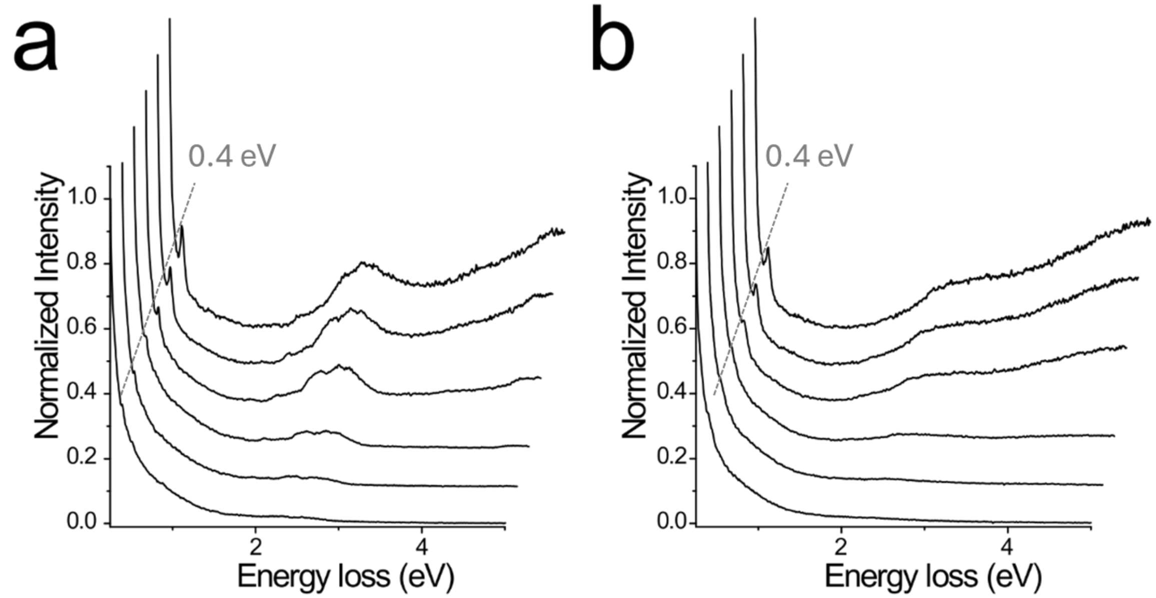

Figure S32. Alternative visualization of the effects of cumulative electron beam damage on EELS at optical energies. The identical spectra to **Figure S31** shown as a 'waterfall' plot (with vertical and horizontal offsets applied between spectra). Spectra from (a) the first pass and (b) the second pass showing degradation in the recorded signal. The first spectrum (nearest the horizontal axis in the waterfall plot) is the spectrum furthest from the surface (edge) of the nanobelt. The sharp peak at approximately 0.4 eV (~3300 cm^{-1}, annotated by gray dashed lines) corresponds to the C–H vibrational stretching modes. The C–H stretching mode peak is still visible, albeit with lower intensity, in the second scan. Scan lines are 15 nm apart.

S8.2. Vibrational Signatures in EELS

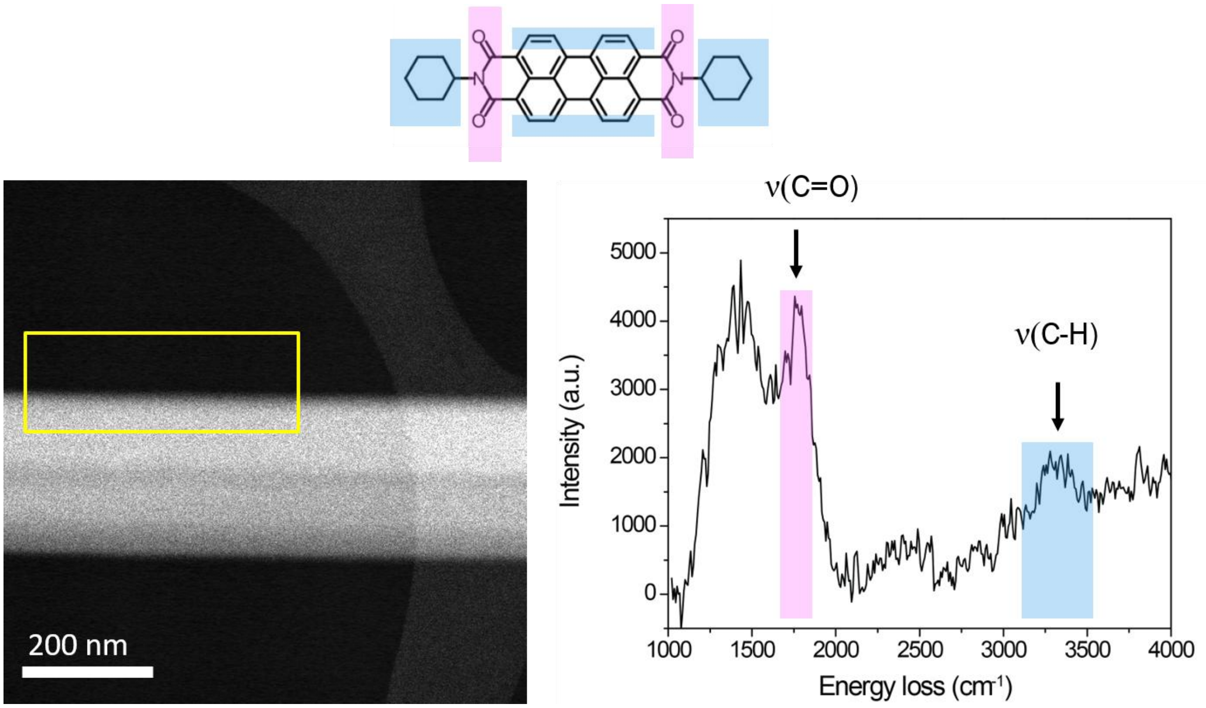

Figure S33. Aloof EELS at vibrational transition energies. The line drawing of the molecule highlights key C=O and C–H stretching moieties in the CH-PDI molecule, (lower right) marked in the spectrum obtained from the indicated spectrum image overlaid on an ADF-STEM image (yellow rectangle, lower left).

S8.3.   Further Examples of Changes in EELS at Defects

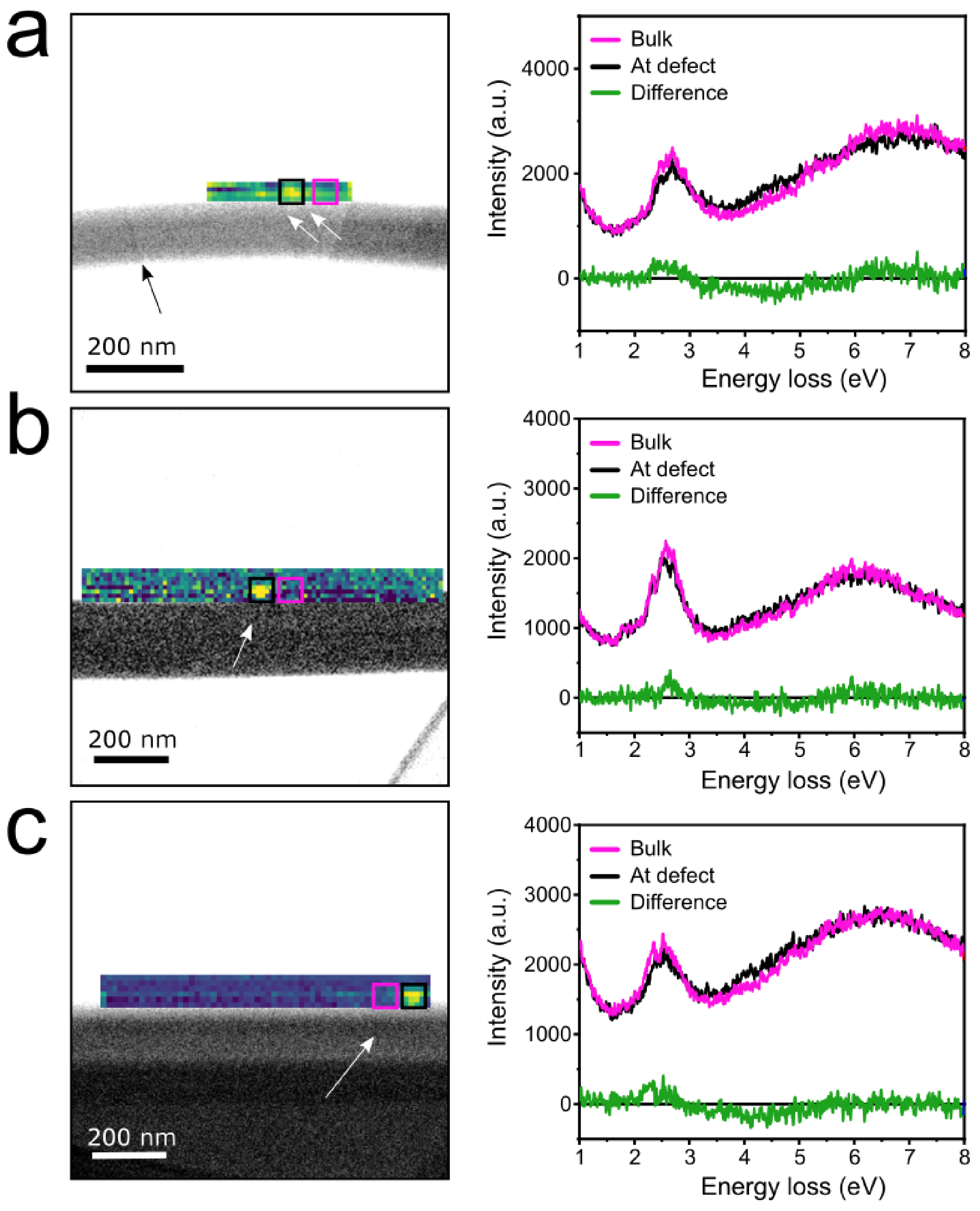

Figure S34. Three further examples of linear defects associated with changes in EEL spectra at defects. In each case, the left presents a BF-STEM image acquired after EELS data were acquired. The EELS acquisition is given by the overlaid *viridis* colourmap representation of an independent component analysis loading map for a localized feature. Arrows mark associated weakly visible lines in the nanobelts in the BF-STEM images. The right-hand column shows the associated spectra away from the defect (magenta squares and spectra), at the defect (black squares and spectra), and difference spectra for each example.

S9. Ab Initio Modeling of CH-PDI and ST-PDI

Table S6. The ten lowest singlet electronic excitations in CH-PDI and ST-PDI computed using TD-DFT. Transitions 2-10 are optically "dark".

Transition	CH-PDI		ST-PDI	
	Energy / eV	Oscillator Strength	Energy / eV	Oscillator Strength
1	2.371	0.756	2.368	0.739
2	3.205	0.000	3.207	0.000
3	3.210	0.000	3.211	0.000
4	3.324	0.000	3.339	0.000
5	3.354	0.000	3.370	0.000
6	3.417	0.000	3.410	0.000
7	3.421	0.000	3.416	0.000
8	3.426	0.000	3.442	0.000
9	3.516	0.000	3.516	0.000
10	3.562	0.000	3.561	0.000

Table S7. Computed Förster couplings between neighboring molecules in nanobelts of CH-PDI and polymorphs I and II of ST-PDI. Stack NN and Stack NNN denote the nearest neighbor (NN) and next-nearest neighbor (NNN) along the $\pi - \pi$ stack, respectively. Inter-stack refers to the strongest inter-stack coupling in the plane perpendicular to the stacking axis.

Position	Förster coupling / meV		
	CH-PDI	Polymorph I of ST-PDI	Polymorph II of ST-PDI
Stack NN	89.1	75.9	73.9
Stack NNN	37.3	31.0	29.2
Inter-stack	11.1	13.8	13.7

S9.1. Charge-transfer (CT)-mediated interaction between exciton states

CT-mediated interaction between PDI molecules has been modelled using a second-order perturbation theory, assuming that the gap between the CT and the molecular Frenkel exciton states is large compared to the electron and hole transfer integrals. In this case the correction to the exciton coupling can be computed as (*80*):

$$J_{\mathrm{CT}} = -\frac{2t_e t_h}{E_{\mathrm{CT}} - E_{\mathrm{F}}} \tag{S1}$$

where t_e and t_h are the electron and hole transfer integrals, E_{CT} is the CT-state energy and E_{F} is the Frenkel exciton energy. **Table S8** summarizes the transfer integrals and the CT-mediated coupling J_{CT}^{+}, assuming a positive splitting $E_{\mathrm{CT}} - E_{\mathrm{F}} = 150$ meV (*80*), and the CT-mediated coupling J_{CT}^{-}, assuming a negative splitting $E_{\mathrm{CT}} - E_{\mathrm{F}} = -390$ meV. The latter value was obtained from DFT calculations at the B3LYP/def2-SVP level.

Table S8. Computed terms for evaluating charge-transfer mediated interactions in CH-PDI and polymorphs I and II of ST-PDI. Electron and hole transfer integrals, $t_e$and t_h, computed for nearest-neighbor stacked molecules from the splitting of the HOMO and LUMO levels in molecular dimers, and the corresponding CT-coupling terms. The signs of the transfer integrals were determined from the symmetry of the contributing orbitals following the procedure of Ref. (*82*) in the main text. The orbitals are visualized in **Figure S35**.

	CH-PDI	Polymorph I of ST-PDI	Polymorph II of ST-PDI
t_e / meV	21	53	52.5
t_h / meV	48	-18.5	-19.5
J_{CT}^{+} / meV	-13.4	13.1	13.6
J_{CT}^{-} / meV	5.2	-5.4	-5.7

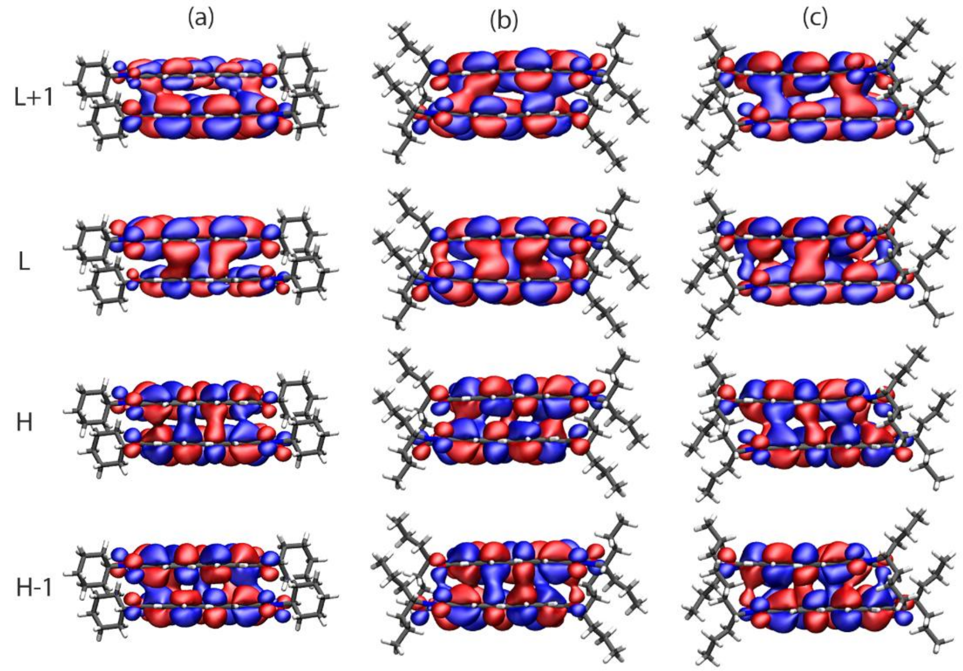

Figure S35. **Visualization of frontier orbitals in CH-PDI and polymorphs I and II of ST-PDI.** Frontier orbitals for dimers of (a) CH-PDI, (b) polymorph I of ST-PDI, and (c) polymorph II of ST-PDI.

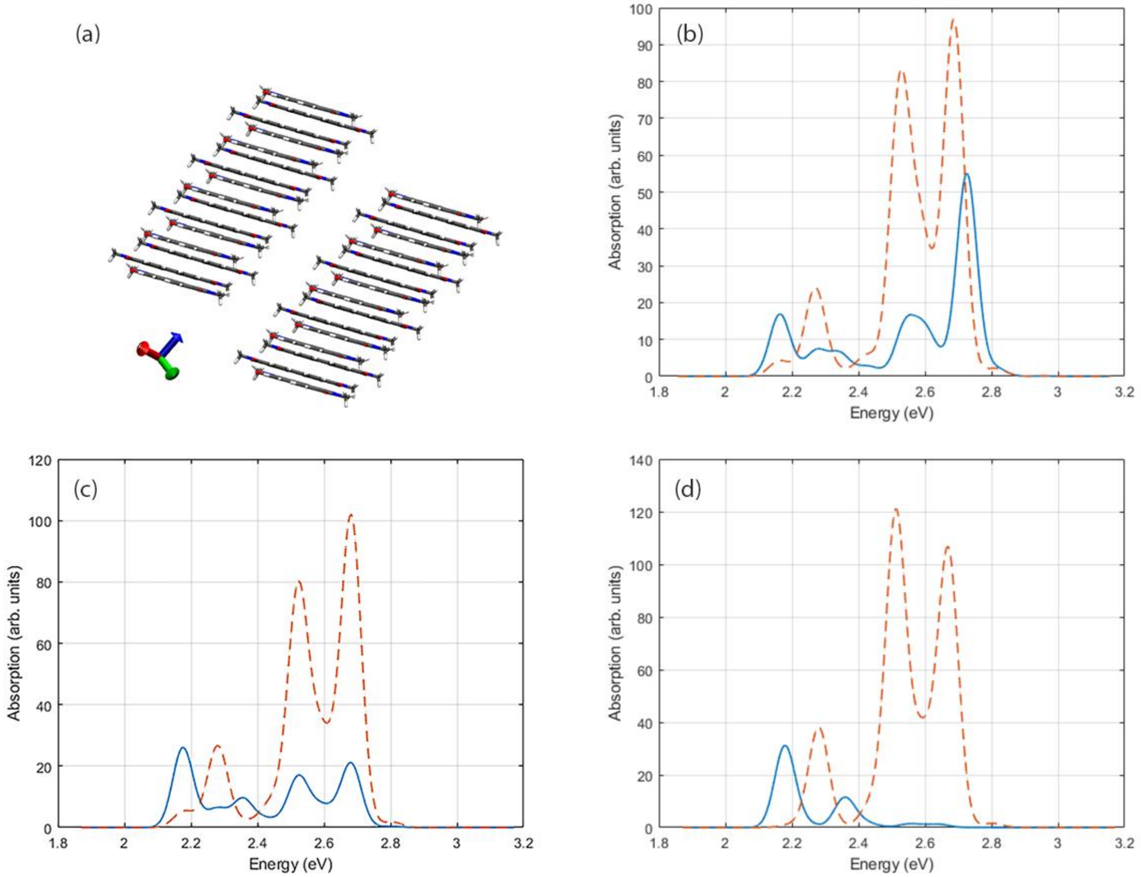

Figure S36. Calculated vibronic spectra for aggregates of CH-PDI and polymorphs I and II of ST-PDI. Polarization-dependent vibronic spectra of small aggregates comprising 32 molecules (2×2 stacks with 8 molecules along the $\pi - \pi$ stacking direction). (a) Relative orientation of the molecular stacks. Two polarization directions, orthogonal to the stack axis (blue), are illustrated in panels (b)-(d) for (b) CH-PDI, (c) polymorph I of ST-PDI, and (d) polymorph II of ST-PDI. The spectra were computed using a two-particle truncation model with displaced-oscillator Franck–Condon factors (Huang–Rhys factor $S = 0.6$) and a single effective vibrational mode $\omega_{\mathrm{vib}} = 1400$ cm^{-1}. The monomer transition energy was set to $E_0 = 2.25$ eV, and up to two vibrational quanta were included in both the ground and excited states. Each spectrum was broadened using a Gaussian with a standard deviation $\sigma = 30$ meV.

Table S9. Computed exciton diffusion coefficients as a function of angular disorder in molecular stacks corresponding to those in the crystal structures of CH-PDI and polymorphs I and II of ST-PDI. The diffusion coefficients are quantified in terms of the parameter $\Delta\theta_{max}$ defined in **Section 2.5** of the paper.

$\Delta\theta_{max}$ / °	Diffusion coefficient / $cm^2 s^{-1}$		
	CH-PDI	Polymorph I of ST-PDI	Polymorph II of ST-PDI
0	45.4	28.2	24.6
10	38.5	25.5	21.6
20	28.0	16.7	16.0
30	18.6	9.1	8.9
40	11.2	6.9	5.1
50	7.6	4.0	4.5
60	5.6	2.6	3.5
70	4.7	2.1	2.3
80	2.7	1.5	1.8
90	2.5	1.1	0.9

S10. Three-dimensional Electron Diffraction – Data Acquisition

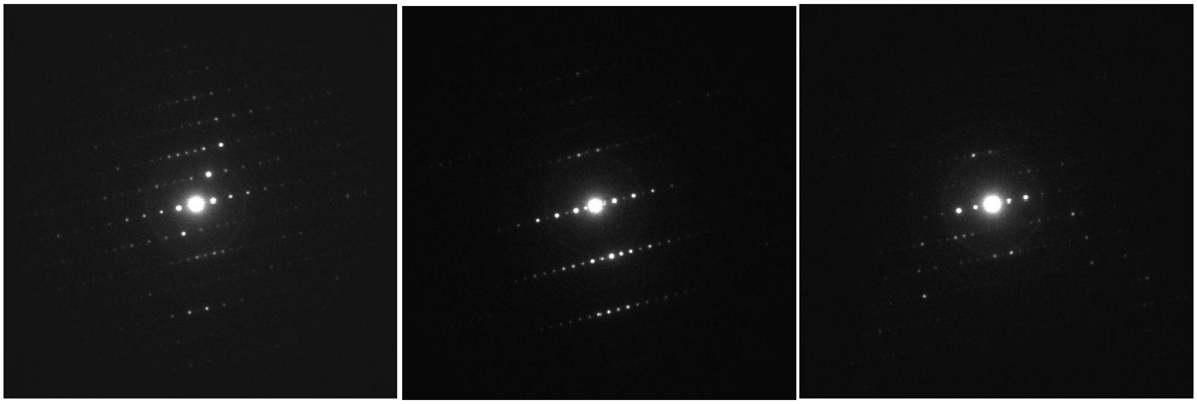

Figure S37. Selected electron diffraction patterns from a 3D-ED dataset recorded for CH-PDI. The selected patterns are from orientations close to crystallographic zone axes.

S11. Crystal Structures After Periodic DFT-D Geometry Optimization

File_S1_CH-PDI_DFT-D_GeometryOptimization.cif
File S1: CIF for the crystal structure generated after subjecting the refined crystal structure of CH-PDI to periodic DFT-D geometry optimization.

File_S2_ST-PDI_Polymorph-I_DFT-D_GeometryOptimization.cif
File S2: CIF for the crystal structure generated after subjecting the refined crystal structure of polymorph I of ST-PDI to periodic DFT-D geometry optimization.

File_S3_ST-PDI_Polymorph-II_DFT-D_GeometryOptimization.cif
File S3: CIF for the crystal structure generated after subjecting the refined crystal structure of polymorph II of ST-PDI to periodic DFT-D geometry optimization.

REFERENCES

1. S. M. Menke, R. J. Holmes, Exciton diffusion in organic photovoltaic cells. *Energ. Environ. Sci.* **7**, 499–512 (2014).

2. O. V. Mikhnenko, P. W. M. Blom, T.-Q. Nguyen, Exciton diffusion in organic semiconductors. *Energ. Environ. Sci.* **8**, 1867–1888 (2015).

3. J. C. Bolinger, M. C. Traub, T. Adachi, P. F. Barbara, Ultralong-range polaron-induced quenching of excitons in isolated conjugated polymers. *Science* **331**, 565–567 (2011).

4. J. Vogelsang, T. Adachi, J. Brazard, D. A. Vanden Bout, P. F. Barbara, Self-assembly of highly ordered conjugated polymer aggregates with long-range energy transfer. *Nat. Mater.* **10**, 942–946 (2011).

5. A. T. Haedler, K. Kreger, A. Issac, B. Wittmann, M. Kivala, N. Hammer, J. Köhler, H.-W. Schmidt, R. Hildner, Long-range energy transport in single supramolecular nanofibres at room temperature. *Nature* **523**, 196–199 (2015).

6. H. Lin, R. Camacho, Y. Tian, T. E. Kaiser, F. Würthner, I. G. Scheblykin, Collective fluorescence blinking in linear J-aggregates assisted by long-distance exciton migration. *Nano Lett.* **10**, 620–626 (2010).

7. X.-H. Jin, M. B. Price, J. R. Finnegan, C. E. Boott, J. M. Richter, A. Rao, S. M. Menke, R. H. Friend, G. R. Whittell, I. Manners, Long-range exciton transport in conjugated polymer nanofibers prepared by seeded growth. *Science* **360**, 897–900 (2018).

8. J. R. Caram, S. Doria, D. M. Eisele, F. S. Freyria, T. S. Sinclair, P. Rebentrost, S. Lloyd, M. G. Bawendi, Room-temperature micron-scale exciton migration in a stabilized emissive molecular aggregate. *Nano Lett.* **16**, 6808–6815 (2016).

9. S. Fratini, M. Nikolka, A. Salleo, G. Schweicher, H. Sirringhaus, Charge transport in high-mobility conjugated polymers and molecular semiconductors. *Nat. Mater.* **19**, 491–502 (2020).

10. P. A. Banks, A. M. Dyer, A. C. Whalley, M. T. Ruggiero, Side-chain torsional dynamics strongly influence charge transport in organic semiconductors. *Chem. Commun.* **58**, 12803–12806 (2022).

11. P. Banks, G. D'Avino, G. Schweicher, J. Armstrong, C. Ruzie, J. W. Chung, C. Sawabe, J.-I. Park, T. Okamoto, J. Takeya, H. Sirringhaus, M. Ruggiero, Untangling the fundamental electronic origins of non-local electron-phonon coupling in organic semiconductors. *Adv. Funct. Mater.* **33**, 2303701 (2023).

12. S. Illig, A. S. Eggeman, A. Troisi, L. Jiang, C. Warwick, M. Nikolka, G. Schweicher, S. G. Yeates, Y. Henri Geerts, J. E. Anthony, H. Sirringhaus, Reducing dynamic disorder in small-molecule organic semiconductors by suppressing large-amplitude thermal motions. *Nat. Commun.* **7**, 10736 (2016).

13. A. S. Eggeman, S. Illig, A. Troisi, H. Sirringhaus, P. A. Midgley, Measurement of molecular motion in organic semiconductors by thermal diffuse electron scattering. *Nat. Mater.* **12**, 1045–1049 (2013).

14. G. Schweicher, G. D'Avino, M. T. Ruggiero, D. J. Harkin, K. Broch, D. Venkateshvaran, G. Liu, A. Richard, C. Ruzié, J. Armstrong, A. R. Kennedy, K. Shankland, K. Takimiya, Y. H. Geerts, J. A. Zeitler, S. Fratini, H. Sirringhaus, Chasing the "killer" phonon mode for the rational design of low-disorder, high-mobility molecular semiconductors. *Adv. Mater.* **31**, 1902407 (2019).

15. M. D. Cohen, Z. Ludmer, J. M. Thomas, J. O. Williams, K. Y. Lonsdale, The role of structural imperfections in the photodimerization of 9-cyanoanthracene. *Proc. A* **324**, 459–468 (1971).

16. J. O. Williams, J. M. Thomas, Photochemical reactions inside the electron microscope: Preferred dimerization of anthracene at dislocations. *Mol. Cryst. Liq. Cryst.* **16**, 371–375 (1972).

17. H. F. Haneef, A. M. Zeidell, O. D. Jurchescu, Charge carrier traps in organic semiconductors: A review on the underlying physics and impact on electronic devices. *J. Mater. Chem. C* **8**, 759–787 (2020).

18. A. J. Sneyd, T. Fukui, D. Paleček, S. Prodhan, I. Wagner, Y. Zhang, J. Sung, S. M. Collins, T. J. A. Slater, Z. Andaji-Garmaroudi, L. R. MacFarlane, J. D. Garcia-Hernandez, L. Wang, G. R. Whittell, J. M. Hodgkiss, K. Chen, D. Beljonne, I. Manners, R. H. Friend, A. Rao, Efficient energy transport in an organic semiconductor mediated by transient exciton delocalization. *Sci. Adv.* **7**, eabh4232 (2021).

19. R. Pandya, R. Y. S. Chen, Q. Gu, J. Sung, C. Schnedermann, O. S. Ojambati, R. Chikkaraddy, J. Gorman, G. Jacucci, O. D. Onelli, T. Willhammar, D. N. Johnstone, S. M. Collins, P. A. Midgley, F. Auras, T. Baikie, R. Jayaprakash, F. Mathevet, R. Soucek, M. Du, A. M. Alvertis, A. Ashoka, S. Vignolini, D. G. Lidzey, J. J. Baumberg, R. H. Friend, T. Barisien, L. Legrand, A. W. Chin, J. Yuen-Zhou, S. K. Saikin, P. Kukura, A. J. Musser, A. Rao, Microcavity-like exciton-polaritons can be the primary photoexcitation in bare organic semiconductors. *Nat. Commun.* **12**, 6519 (2021).

20. C. Schnedermann, J. M. Lim, T. Wende, A. S. Duarte, L. Ni, Q. Gu, A. Sadhanala, A. Rao, P. Kukura, Sub-10 fs time-resolved vibronic optical microscopy. *J. Phys. Chem. Lett.* **7**, 4854–4859 (2016).

21. J. Sung, C. Schnedermann, L. Ni, A. Sadhanala, R. Y. S. Chen, C. Cho, L. Priest, J. M. Lim, H.-K. Kim, B. Monserrat, P. Kukura, A. Rao, Long-range ballistic propagation of carriers in methylammonium lead iodide perovskite thin films. *Nat. Phys.* **16**, 171–176 (2020).

22. M. Ilett, M. S'ari, H. Freeman, Z. Aslam, N. Koniuch, M. Afzali, J. Cattle, R. Hooley, T. Roncal-Herrero, S. M. Collins, N. Hondow, A. Brown, R. Brydson, Analysis of complex, beam-sensitive materials by transmission electron microscopy and associated techniques. *Philos. Trans. A. Math Phys. Eng. Sci.* **378**, 20190601 (2020).

23. C. J. H. Smalley, H. E. Hoskyns, C. E. Hughes, D. N. Johnstone, T. Willhammar, M. T. Young, C. J. Pickard, A. J. Logsdail, P. A. Midgley, K. D. M. Harris, A structure determination protocol based on combined analysis of 3D-ED data, powder XRD data,

solid-state NMR data and DFT-D calculations reveals the structure of a new polymorph of L-tyrosine. *Chem. Sci.* **13**, 5277–5288 (2022).

24. K. D. M. Harris, R. L. Johnston, B. M. Kariuki, The genetic algorithm: Foundations and applications in structure solution from powder diffraction data. *Acta Crystallogr. A* **54**, 632–645 (1998).

25. C. J. H. Smalley, C. E. Hughes, M. Hildebrand, R. Aizen, M. Bauer, A. Yamano, D. Levy, S. K. Mirsky, N. T. Shaked, M. T. Young, U. Kolb, E. Gazit, L. Kronik, K. D. M. Harris, Understanding the solid-state structure of riboflavin through a multitechnique approach. *Cryst. Growth Des.* **24**, 6256–6266 (2024).

26. O. Panova, C. Ophus, C. J. Takacs, K. C. Bustillo, L. Balhorn, A. Salleo, N. Balsara, A. M. Minor, Diffraction imaging of nanocrystalline structures in organic semiconductor molecular thin films. *Nat. Mater.* **18**, 860–865 (2019).

27. K. C. Bustillo, S. E. Zeltmann, M. Chen, J. Donohue, J. Ciston, C. Ophus, A. M. Minor, 4D-STEM of beam-sensitive materials. *Acc. Chem. Res.* **54**, 2543–2551 (2021).

28. J. A. Alexander, F. J. Scheltens, L. F. Drummy, M. F. Durstock, J. B. Gilchrist, S. Heutz, D. W. McComb, Measurement of optical properties in organic photovoltaic materials using monochromated electron energy-loss spectroscopy. *J. Mater. Chem. A* **4**, 13636–13645 (2016).

29. C. Guo, F. I. Allen, Y. Lee, T. P. Le, C. Song, J. Ciston, A. M. Minor, E. D. Gomez, Probing local electronic transitions in organic semiconductors through energy-loss spectrum imaging in the transmission electron microscope. *Adv. Funct. Mater.* **25**, 6071–6076 (2015).

30. P. Rez, T. Aoki, K. March, D. Gur, O. L. Krivanek, N. Dellby, T. C. Lovejoy, S. G. Wolf, H. Cohen, Damage-free vibrational spectroscopy of biological materials in the electron microscope. *Nat. Commun.* **7**, 10945 (2016).

31. P. A. Crozier, Vibrational and valence aloof beam EELS: A potential tool for nondestructive characterization of nanoparticle surfaces. *Ultramicroscopy* **180**, 104–114 (2017).

32. D. Burmeister, A. Eljarrat, M. Guerrini, E. Röck, J. Plaickner, C. T. Koch, N. Banerji, C. Cocchi, E. J. W. List-Kratochvil, M. J. Bojdys, On the non-bonding valence band and the electronic properties of poly(triazine imide), a graphitic carbon nitride. *Chem. Sci.* **14**, 6269–6277 (2023).

33. P. P. Das, A. Mazumder, M. Rajeevan, R. Srinivasamurthy Swathi, M. Hariharan, Energy landscape of perylenediimide chromophoric aggregates. *Phys. Chem. Chem. Phys.* **26**, 2007–2015 (2024).

34. E. Sebastian, A. M. Philip, A. Benny, M. Hariharan, Null exciton splitting in chromophoric Greek cross (+) aggregate. *Angew. Chem. Int. Ed.* **57**, 15696–15701 (2018).

35. C. Huang, S. Barlow, S. R. Marder, Perylene-3,4,9,10-tetracarboxylic acid diimides: Synthesis, physical properties, and use in organic electronics. *J. Org. Chem.* **76**, 2386–2407 (2011).

36. A. Ashoka, N. Gauriot, A. V. Girija, N. Sawhney, A. J. Sneyd, K. Watanabe, T. Taniguchi, J. Sung, C. Schnedermann, A. Rao, Direct observation of ultrafast singlet exciton fission in three dimensions. *Nat. Commun.* **13**, 5963 (2022).

37. A. Le Bail, H. Duroy, J. L. Fourquet, Ab-initio structure determination of $LiSbWO_6$ by x-ray powder diffraction. *Mater. Res. Bull.* **23**, 447–452 (1988).

38. B. M. Kariuki, H. Serrano-González, R. L. Johnston, K. D. M. Harris, The application of a genetic algorithm for solving crystal structures from powder diffraction data. *Chem. Phys. Lett.* **280**, 189–195 (1997).

39. E. Y. Cheung, E. E. McCabe, K. D. M. Harris, R. L. Johnston, E. Tedesco, K. M. P. Raja, P. Balaram, C−H···O hydrogen bond mediated chain reversal in a peptide containing a γ-amino acid residue, determined directly from powder x-ray diffraction data. *Angew. Chem. Int. Ed. Engl.* **41**, 494–496 (2002).

40. S. Habershon, K. D. M. Harris, R. L. Johnston, Development of a multipopulation parallel genetic algorithm for structure solution from powder diffraction data. *J. Comput. Chem.* **24**, 1766–1774 (2003).

41. F. Guo, K. D. M. Harris, Structural understanding of a molecular material that is accessed only by a solid-state desolvation process: The scope of modern powder x-ray diffraction techniques. *J. Am. Chem. Soc.* **127**, 7314–7315 (2005).

42. X. Ma, G. K. Lim, K. D. M. Harris, D. C. Apperley, P. N. Horton, M. B. Hursthouse, S. L. James, Efficient, scalable, and solvent-free mechanochemical synthesis of the OLED material Alq3 (q = 8-hydroxyquinolinate). *Cryst. Growth Des.* **12**, 5869–5872 (2012).

43. P. A. Williams, C. E. Hughes, K. D. M. Harris, L-lysine: Exploiting powder x-ray diffraction to complete the set of crystal structures of the 20 directly encoded proteinogenic amino acids. *Angew. Chem. Int. Ed. Engl.* **54**, 3973–3977 (2015).

44. C. E. Hughes, G. N. M. Reddy, S. Masiero, S. P. Brown, P. A. Williams, K. D. M. Harris, Determination of a complex crystal structure in the absence of single crystals: analysis of powder x-ray diffraction data, guided by solid-state NMR and periodic DFT calculations, reveals a new 2′-deoxyguanosine structural motif. *Chem. Sci.* **8**, 3971–3979 (2017).

45. T. Sun, C. E. Hughes, L. Guo, L. Wei, K. D. M. Harris, Y.-B. Zhang, Y. Ma, Direct-space structure determination of covalent organic frameworks from 3D electron diffraction data. *Angew. Chem. Int. Ed. Engl.* **59**, 22638–22644 (2020).

46. A. Wagner, J. Merkelbach, L. Samperisi, N. Pinsk, B. M. Kariuki, C. E. Hughes, K. D. M. Harris, B. A. Palmer, Structure determination of biogenic crystals directly from 3D electron diffraction data. *Cryst. Growth Des.* **24**, 899–905 (2024).

47. I. Lobato, D. Van Dyck, An accurate parameterization for scattering factors, electron densities and electrostatic potentials for neutral atoms that obey all physical constraints. *Acta Crystallogr. A Found. Adv.* **70**, 636–649 (2014).

48. A. March, Mathematische Theorie der Regelung nach der Korngestah bei affiner Deformation. *Z. Kristallogr. Cryst. Mater.* **81**, 285–297 (1932).

49. W. A. Dollase, Correction of intensities for preferred orientation in powder diffractometry: Application of the March model. *J. Appl. Cryst.* **19**, 267–272 (1986).

50. C. A. Hunter, J. K. M. Sanders, The nature of .pi.-.pi. interactions. *J. Am. Chem. Soc.* **112**, 5525–5534 (1990).

51. C. E. Hughes, B. M. Kariuki, A. Almetahr, J. Saint-Mart, A. Williams, L. Samperisi, K. D. M. Harris, Solid-state structure of xanthine determined by a combination of 3D electron diffraction, powder x-ray diffraction, and DFT-D calculations. *Cryst. Growth Des.* **25**, 895–902 (2025).

52. Y. Yun, X. Zou, S. Hovmöller, W. Wan, Three-dimensional electron diffraction as a complementary technique to powder x-ray diffraction for phase identification and structure solution of powders. *IUCrJ* **2**, 267–282 (2015).

53. A. J. Cruz-Cabeza, S. M. Reutzel-Edens, J. Bernstein, Facts and fictions about polymorphism. *Chem. Soc. Rev.* **44**, 8619–8635 (2015).

54. J. Nyman, G. M. Day, Static and lattice vibrational energy differences between polymorphs. *CrstEngComm* **17**, 5154–5165 (2015).

55. T. E. Gorelik, C. Czech, S. M. Hammer, M. U. Schmidt, Crystal structure of disordered nanocrystalline αII-quinacridone determined by electron diffraction. *CrstEngComm* **18**, 529–535 (2016).

56. S. J. Clark, M. D. Segall, C. J. Pickard, P. J. Hasnip, M. I. J. Probert, K. Refson, M. C. Payne, First principles methods using CASTEP. *Z. Kristallogr. Cryst. Mater.* **220**, 567–570 (2005).

57. C. J. Pickard, F. Mauri, All-electron magnetic response with pseudopotentials: NMR chemical shifts. *Phys. Rev. B* **63**, 245101 (2001).

58. J. R. Yates, C. J. Pickard, F. Mauri, Calculation of NMR chemical shifts for extended systems using ultrasoft pseudopotentials. *Phys. Rev. B* **76**, 024401 (2007).

59. T. Charpentier, The PAW/GIPAW approach for computing NMR parameters: A new dimension added to NMR study of solids. *Solid State Nucl. Magn. Reson.* **40**, 1–20 (2011).

60. S. J. Opella, M. H. Frey, Selection of nonprotonated carbon resonances in solid-state nuclear magnetic resonance. *J. Am. Chem. Soc.* **101**, 5854–5856 (1979).

61. L. B. Alemany, D. M. Grant, T. D. Alger, R. J. Pugmire, Cross polarization and magic angle sample spinning NMR spectra of model organic compounds. 3. Effect of the carbon-13-proton dipolar interaction on cross polarization and carbon-proton dephasing. *J. Am. Chem. Soc.* **105**, 6697–6704 (1983).

62. A. E. Aliev, K. D. M. Harris, P. H. Champkin, Structural and dynamic aspects of hydrogen-bonded complexes and inclusion compounds containing α,ω-dicyanoalkanes and urea, investigated by solid-state ^{13}C and ^{2}H NMR techniques. *J. Phys. Chem. B* **109**, 23342–23350 (2005).

63. A. E. Aliev, Solid-state NMR studies of collagen-based parchments and gelatin. *Biopolymers* **77**, 230–245 (2005).

64. B. A. Palmer, B. M. Kariuki, V. K. Muppidi, C. E. Hughes, K. D. M. Harris, An incommensurate thiourea inclusion compound. *Chem. Commun.* **47**, 3760–3762 (2011).

65. A. E. Aliev, D. Courtier-Murias, Water scaffolding in collagen: Implications on protein dynamics as revealed by solid-state NMR. *Biopolymers* **101**, 246–256 (2014).

66. A. E. Aliev, D. Courtier-Murias, Concise NMR approach for molecular dynamics characterizations in organic solids. *J. Phys. Chem. A* **117**, 7855–7862 (2013).

67. O. Al Rahal, B. M. Kariuki, C. E. Hughes, P. A. Williams, X. Xu, S. Gaisford, D. Iuga, K. D. M. Harris, Unraveling the complex solid-state phase transition behavior of 1-iodoadamantane, a material for which ostensibly identical crystals undergo different transformation pathways. *Cryst. Growth Des.* **23**, 3820–3833 (2023).

68. W. Maniukiewicz, J. Bojarska, A. Olczak, E. Dobruchowska, M. Wiatrowski, 2,9-Di-3-pentylanthra[1,9-def:6,5,10-d′e′f′]diisoquinoline-1,3,8,10-tetrone. *Acta Crystallogr. Sect. E Struct. Rep. Online* **66**, o2570–o2571 (2010).

69. R. Mathew, A. Mazumder, P. Kumar, J. Matula, S. Mohamed, P. Brazda, M. Hariharan, B. Thomas, Unveiling the topology of partially disordered micro-crystalline nitro-perylenediimide with X-aggregate stacking: An integrated approach. *Chem. Sci.* **15**, 490–499 (2024).

70. S. T. Pham, N. Koniuch, E. Wynne, A. Brown, S. M. Collins, Microscopic crystallographic analysis of dislocations in molecular crystals. *Nat. Mater.* **24**, 682–687 (2025).

71. D. N. Johnstone, F. C. N. Firth, C. P. Grey, P. A. Midgley, M. J. Cliffe, S. M. Collins, Direct imaging of correlated defect nanodomains in a metal–organic framework. *J. Am. Chem. Soc.* **142**, 13081–13089 (2020).

72. E. Wynne, S. D. Connell, R. Shinebaum, H. Blade, N. George, A. Brown, S. M. Collins, Grain and domain microstructure in long chain n-alkane and n-alkanol wax crystals. *Cryst. Growth Des.* **24**, 10127–10142 (2024).

73. C. J. Humphreys, A. Howie, G. R. Booker, Some electron diffraction contrast effects at planar defects in crystals. *Philos. Mag.-J. Theor. Exp. Appl. Phys.* **15**, 507–522 (1967).

74. V. F. Holland, Dislocations in polyethylene single crystals. *J. Appl. Phys.* **35**, 3235–3241 (1964).

75. H. Leung, R. Copley, G. Lampronti, S. Day, L. Saunders, D. Johnstone, P. Midgley, Polytypes and planar defects revealed in the purine base xanthine using multi-dimensional electron diffraction. *Commun. Chem.* **8**, 331 (2025).

76. E. Spiecker, W. Jäger, Burgers vector analysis of large area misfit dislocation arrays from bend contour contrast in transmission electron microscope images. *J. Phys. Condens. Matter* **14**, 12767–12776 (2002).

77. I. A. Olson, A. G. Shtukenberg, G. Hakobyan, A. L. Rohl, P. Raiteri, M. D. Ward, B. Kahr, Structure, energetics, and dynamics of screw dislocations in even n-alkane crystals. *J. Phys. Chem. Lett.* **7**, 3112–3117 (2016).

78. N. Ide, I. Okada, K. Kojima, Computer simulation of core structure and Peierls stress of dislocations in anthracene crystals. *J. Phys. Condens. Matter* **5**, 3151–3162 (1993).

79. S. Valleau, S. K. Saikin, M.-H. Yung, A. A. Guzik, Exciton transport in thin-film cyanine dye J-aggregates. *J. Chem. Phys.* **137**, 034109 (2012).

80. H. Yamagata, D. S. Maxwell, J. Fan, K. R. Kittilstved, A. L. Briseno, M. D. Barnes, F. C. Spano, HJ-aggregate behavior of crystalline 7,8,15,16-tetraazaterrylene: Introducing a new design paradigm for organic materials. *J. Phys. Chem. C* **118**, 28842–28854 (2014).

81. W. Liu, V. Settels, P. H. P. Harbach, A. Dreuw, R. F. Fink, B. Engels, Assessment of TD-DFT- and TD-HF-based approaches for the prediction of exciton coupling parameters, potential energy curves, and electronic characters of electronically excited aggregates. *J. Comput. Chem.* **32**, 1971–1981 (2011).

82. L. Gisslén, R. Scholz, Crystallochromy of perylene pigments: Interference between Frenkel excitons and charge-transfer states. *Phys. Rev. B* **80**, 115309 (2009).

83. N. J. Hestand, R. Tempelaar, J. Knoester, T. L. C. Jansen, F. C. Spano, Exciton mobility control through sub−Å packing modifications in molecular crystals. *Phys. Rev. B* **91**, 195315 (2015).

84. A. Segalina, X. Assfeld, A. Monari, M. Pastore, Computational modeling of exciton localization in self-assembled perylene helices: Effects of thermal motion and aggregate size. *J. Phys. Chem. C* **123**, 6427–6437 (2019).

85. J. Sung, P. Kim, B. Fimmel, F. Würthner, D. Kim, Direct observation of ultrafast coherent exciton dynamics in helical π-stacks of self-assembled perylene bisimides. *Nat. Commun.* **6**, 8646 (2015).

86. M. Zubiria-Ulacia, J. M. Matxain, D. Casanova, The role of CT excitations in PDI aggregates. *Phys. Chem. Chem. Phys.* **22**, 15908–15918 (2020).

87. A. L. Bialas, F. C. Spano, A Holstein–Peierls approach to excimer spectra: The evolution from vibronically structured to unstructured emission. *J. Phys. Chem. C* **126**, 4067–4081 (2022).

88. F. Marin, S. Tombolesi, T. Salzillo, O. Yaffe, L. Maini, Thorough investigation on the high-temperature polymorphism of dipentyl-perylenediimide: Thermal expansion vs. polymorphic transition. *J. Mater. Chem. C* **10**, 8089–8100 (2022).

89. Y. Che, X. Yang, K. Balakrishnan, J. Zuo, L. Zang, Highly polarized and self-waveguided emission from single-crystalline organic nanobelts. *Chem. Mater.* **21**, 2930–2934 (2009).

90. H. Horinouchi, H. Sakai, Y. Araki, T. Sakanoue, T. Takenobu, T. Wada, N. V. Tkachenko, T. Hasobe, Controllable electronic structures and photoinduced processes of bay-linked perylenediimide dimers and a ferrocene-linked triad. *Chem. A Eur. J.* **22**, 9631–9641 (2016).

91. A. C. Larson, R. B. Von Dreele, "Los Alamos National Laboratory Report" (2004).

92. M. O. Cichocka, J. Ångström, B. Wang, X. Zou, S. Smeets, High-throughput continuous rotation electron diffraction data acquisition via software automation. *J. Appl. Cryst.* **51**, 1652–1661 (2018).

93. W. Kabsch, XDS. *Acta Crystallogr. D Biol. Crystallogr.* **66**, 125–132 (2010).

94. B. M. Kariuki, K. Psallidas, K. D. M. Harris, R. L. Johnston, R. W. Lancaster, S. E. Staniforth, S. M. Cooper, Structure determination of a steroid directly from powder diffraction data. *Chem. Commun.*, 1677–1678 (1999).

95. G. M. Sheldrick, A short history of SHELX. *Acta Crystallogr. A* **64**, 112–122 (2008).

96. D. Vanderbilt, Soft self-consistent pseudopotentials in a generalized eigenvalue formalism. *Phys. Rev. B* **41**, 7892–7895 (1990).

97. J. P. Perdew, K. Burke, M. Ernzerhof, Generalized gradient approximation made simple. *Phys. Rev. Lett.* **77**, 3865–3868 (1996).

98. A. Tkatchenko, M. Scheffler, Accurate molecular Van Der Waals interactions from ground-state electron density and free-atom reference data. *Phys. Rev. Lett.* **102**, 073005 (2009).

99. H. J. Monkhorst, J. D. Pack, Special points for Brillouin-zone integrations. *Phys. Rev. B* **13**, 5188–5192 (1976).

100. R. K. Harris, P. Hodgkinson, C. J. Pickard, J. R. Yates, V. Zorin, Chemical shift computations on a crystallographic basis: some reflections and comments. *Magn. Reson. Chem.* **45**, S174–S186 (2007).

101. D. N. Johnstone, P. Crout, S. Høgås, T. Bergh, M. Danaie, C. Francis, S. Smeets, D. Weber, pyxem/pyxem-demos: pyxem-demos 0.11.0, Zenodo (2020); https://doi.org/10.5281/zenodo.3831456.

102. R. F. Egerton, *Electron Energy-Loss Spectroscopy in the Electron Microscope* (Springer, New York, ed. 3, 2011).

103. K. Iakoubovskii, K. Mitsuishi, Y. Nakayama, K. Furuya, Thickness measurements with electron energy loss spectroscopy. *Microsc. Res. Tech.* **71**, 626–631 (2008).

104. Francisco de la Peña, Tomas Ostasevicius, Vidar Tonaas Fauske, Pierre Burdet, Eric Prestat, Petras Jokubauskas, Magnus Nord, Mike Sarahan, Katherine E. MacArthur, Duncan N. Johnstone, Joshua Taillon, Jan Caron, Vadim Migunov, Tom Furnival, Alberto Eljarrat, Stefano Mazzucco, Thomas Aarholt, Michael Walls, Tom Slater, Florian Winkler, Ben Martineau, Gaël Donval, Robert McLeod, Eric R. Hoglund, Ivo Alxneit, Ida Hjorth, Trond Henninen, Luiz Fernando Zagonel, Andreas Garmannslund, 5ht2, hyperspy/hyperspy: HyperSpy 1.3.1, Zenodo (2018); https://doi.org/10.5281/zenodo.1221347.

105. S. M. Collins, S. Fernandez-Garcia, J. J. Calvino, P. A. Midgley, Sub-nanometer surface chemistry and orbital hybridization in lanthanum-doped ceria nano-catalysts revealed by 3D electron microscopy. *Sci. Rep.* **7**, 5406 (2017).

106. F. de la Peña, M.-H. Berger, J.-F. Hochepied, F. Dynys, O. Stephan, M. Walls, Mapping titanium and tin oxide phases using EELS: An application of independent component analysis. *Ultramicroscopy* **111**, 169–176 (2011).

107. S. M. Collins, D. M. Kepaptsoglou, J. Hou, C. W. Ashling, G. Radtke, T. D. Bennett, P. A. Midgley, Q. M. Ramasse, Functional group mapping by electron beam vibrational spectroscopy from nanoscale volumes. *Nano Lett.* **20**, 1272–1279 (2020).

108. F. S. Hage, G. Radtke, D. M. Kepaptsoglou, M. Lazzeri, Q. M. Ramasse, Single-atom vibrational spectroscopy in the scanning transmission electron microscope. *Science* **367**, 1124–1127 (2020).

109. R. Ahlrichs, M. Bär, M. Häser, H. Horn, C. Kölmel, Electronic structure calculations on workstation computers: The program system turbomole. *Chem. Phys. Lett.* **162**, 165–169 (1989).

110. F. Weigend, R. Ahlrichs, Balanced basis sets of split valence, triple zeta valence and quadruple zeta valence quality for H to Rn: Design and assessment of accuracy. *Phys. Chem. Chem. Phys.* **7**, 3297–3305 (2005).

111. A. D. Becke, Density-functional thermochemistry. III. The role of exact exchange. *J. Chem. Phys.* **98**, 5648–5652 (1993).

112. T. Förster, in *Modern Quantum Chemistry*, O. Sinanoğlu, Ed. (Academic Press, New York, 1965), vol. 3, pp. 93–137.

113. S. K. Saikin, A. Eisfeld, S. Valleau, A. Aspuru-Guzik, Photonics meets excitonics: Natural and artificial molecular aggregates. *Nanophotonics* **2**, 21–38 (2013).

114. R. Pandya, R. Y. S. Chen, Q. Gu, J. Gorman, F. Auras, J. Sung, R. Friend, P. Kukura, C. Schnedermann, A. Rao, Femtosecond transient absorption microscopy of singlet exciton motion in side-chain engineered perylene-diimide thin films. *J. Phys. Chem. A* **124**, 2721–2730 (2020).

115. A. Ashoka, R. R. Tamming, A. V. Girija, H. Bretscher, S. D. Verma, S.-D. Yang, C.-H. Lu, J. M. Hodgkiss, D. Ritchie, C. Chen, C. G. Smith, C. Schnedermann, M. B. Price, K. Chen, A. Rao, Extracting quantitative dielectric properties from pump-probe spectroscopy. *Nat. Commun.* **13**, 1437 (2022).
